# MORC3 represses a tandem repeat enhancer to regulate interferon

**DOI:** 10.1038/s44318-026-00799-9

**Published:** 2026-06-05

**Authors:** Luisa Krumwiede, David Hollaus, Erika Valeri, Karina Schindler-Schumitsch, Monika A Bazyl, Johanna Schiedlbauer, Nanette N Becht, Markus Jaritz, Bernardo P de Almeida, Siegfried Schloissnig, Dara L Burdette, Jacob Schreiber, Alexander Stark, Moritz M Gaidt

**Affiliations:** 1 Research Institute of Molecular Pathology (IMP), Vienna BioCenter (VBC), Vienna, Austria; 2 Vienna BioCenter PhD Program, Doctoral School of the University of Vienna and Medical University of Vienna, Vienna, Austria; 3 Tempest Therapeutics, Brisbane, CA USA; 4 Department of Genomics and Computational Biology, UMass Chan Medical School, Worcester, MA USA; 5 Medical University of Vienna, Vienna BioCenter (VBC), Vienna, Austria; 6 Present Address: InstaDeep, Paris, France

**Keywords:** Chromatin, Transcription & Genomics, Immunology, Microbiology, Virology & Host Pathogen Interaction

## Abstract

The antiviral protein MORC3 is frequently inhibited by viruses. To counteract viral antagonism, MORC3 represses a noncanonical pathway of type-I-interferon (IFN) such that viral inhibition of MORC3 triggers ( > 10,000-fold) IFN induction. How MORC3 represses this pathway, and why IFN induction upon MORC3 loss is so potent without canonical IRF3/7 transcription factors, is unknown. Here, we show that MORC3 restricts chromatin accessibility at tandem repeat elements harboring up to 61 homotypic transcription factor motifs. One such element becomes a potent enhancer of *IFNB1* upon MORC3 loss. Its motif cluster contains 45 PU.1 binding sites and is necessary and sufficient for MORC3-mediated repression and enhancer activity upon MORC3 loss. PU.1 recruits MORC3 to repress this enhancer by recruiting DAXX and enabling H3.3 incorporation. Upon MORC3 loss, PU.1 drives IRF3/7-independent IFN induction. Other restricted tandem repeats contain homotypic motif clusters of SPI, AP-1, and SP/KLF transcription factors. Our findings uncover a TF motif cluster–driven repression mechanism by MORC3 at tandem repeats, enabling specific repression of an IFNB1 enhancer such that viral antagonism of MORC3 induces interferon.

## Synopsis

MORC3 represses a noncanonical interferon pathway, but the underlying gene regulatory logic and mechanism are unclear. This study characterizes a tandem repeat enhancer containing a dense PU.1 motif cluster that recruits MORC3 for repression and drives strong interferon induction when MORC3 is lost.

- MORC3 restricts chromatin accessibility at tandem repeats containing clusters of homotypic transcription factor motifs.
- The IFNB1-MORC3-repressed element (MRE) is a tandem repeat containing 45 PU.1 motifs that functions as a MORC3-repressed enhancer controlling IFNB1 expression.
- PU.1 motif cluster are necessary and sufficient to create a MORC3-repressed enhancer.
- PU.1 recruits MORC3 and DAXX to deposit H3.3 and maintain the MRE in a repressed chromatin state.
- Upon MORC3 loss, unrestricted PU.1 activity at the MRE drives potent IFNB1 induction.

## Abstract (web summary)

A PU.1 motif cluster within a tandem-repeat enhancer recruits MORC3–DAXX to maintain repressive chromatin and activate interferon upon perturbation of MORC3.

## Graphical abstract

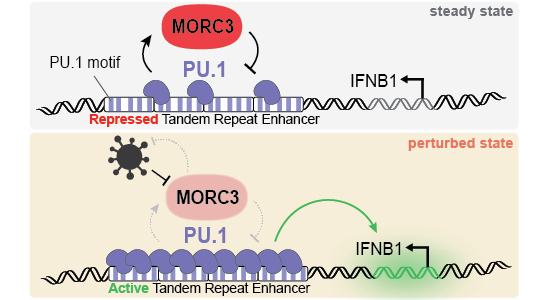

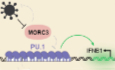

## Introduction

The innate immune system employs restriction factor proteins to antagonize viral replication. In response, viruses deploy virulence factors, also known as effectors, to inhibit restriction factors and allow replication. In an evolutionary arms race between hosts and pathogens, some restriction factors have acquired a secondary function to repress inflammation that serves as an effective “insurance policy”. When a dual-function restriction factor is attacked by virulence factors to overcome its antimicrobial activity, its secondary immunosuppressive function is inadvertently inhibited, thus activating inflammation. This paradigm aligns with the concept of effector-triggered immunity (ETI), where the immune system senses the activities of microbial virulence factors (effectors) (Lopes Fischer et al, 2020; Orzalli and Parameswaran, 2022; Remick et al, 2023).

One of these dual-function restriction factors is MORC3. MORC3 is a gyrase, Hsp90, histidine kinase, MutL (GHKL)-family ATPase, chromatin-binding protein, and transcriptional repressor of endogenous retroviruses (ERVs) and DNA viruses. At ERVs, MORC3 cooperates with DAXX, a chaperone for the histone H3.3, to incorporate H3.3 and H3K9me3, and induce repression (Groh et al, 2021). MORC3 also silences transcription of several DNA viral genomes including HSV-1, CMV and AAV driving viral latency (Champion et al, 2023; Ma et al, 2022; Ngo and Puschnik, 2023; Sloan et al, 2016). The ability of MORC3 to repress viral gene expression may be connected to its localization in ND10 nuclear bodies (Mimura et al, 2010), as these membraneless nuclear organelles have similar antiviral functions (Scherer and Stamminger, 2016). To allow lytic replication, DNA viruses express virulence factors (e.g., HSV-1 ICP0, Adenovirus 5 E4ORF3) that disrupt ND10 bodies and degrade or inhibit MORC3 (Sloan et al, 2015; Ullman and Hearing, 2008). However, as an insured protein, MORC3 has acquired a secondary immunosuppressive function and inhibits a noncanonical pathway of type I interferon (IFN-β) activation, a potent antiviral cytokine (Gaidt et al, 2021b). Viral antagonism of MORC3 inadvertently removes this repression, leading to robust (> 10,000-fold) IFN production. The IFN response operates independently of the canonical IFN transcription factors IRF3/7 and STAT1/2 (Gaidt et al, 2021b), but requires a poorly characterized cis-regulatory element termed the MORC3-regulated element (MRE). However, how MORC3 specifically represses this DNA element, how a single element can induce such a potent IFN response, and the involved transcription factor are unknown.

Here, we show that MORC3 restricts chromatin accessibility at tandem repeat elements that harbor exceptionally dense clusters of homotypic transcription factor binding motifs. The IFNB1-MRE is a tandem repeat containing 45 PU.1 binding sites, and PU.1 mediates IRF3/7-independent *IFNB1* induction through this element. In steady-state conditions, MORC3 represses MRE activity by recruiting DAXX and enabling H3.3 incorporation, thereby maintaining the element in a compacted chromatin state. Notably, PU.1 motif clusters mediate both MORC3 recruitment—leading to repression at steady state—and exceptionally strong, synergistic enhancer activity of *IFNB1* induction upon MORC3 loss. Together, our findings identify homotypic motif clusters as the basis of a repressed enhancer that functions as an immune “insurance mechanism,” enabling robust interferon induction upon perturbation of MORC3.

## Results

### MORC3 limits chromatin accessibility at tandem repeats

**Figure 1:**
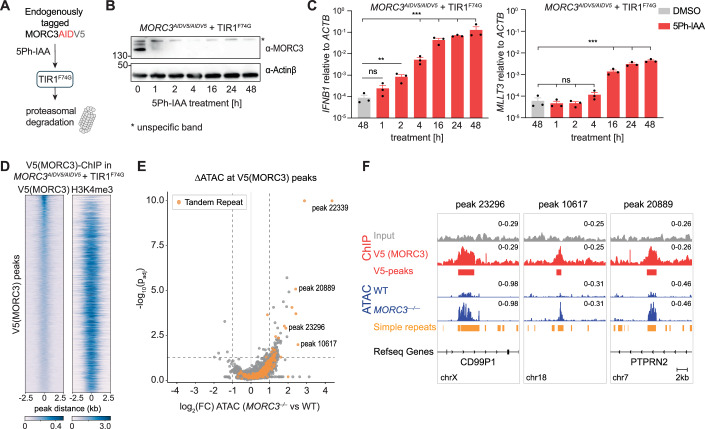
MORC3 limits chromatin accessibility at several tandem repeats. (**A**) Schematic illustration of the degron approach. Endogenously tagged MORC3-AID-V5 is degraded upon 5Ph-IAA treatment in TIR1^F74G^ expressing BLaER1 monocytes. (**B**, **C**) Immunoblot analysis and gene expression in *IFNAR1*^–/–^ *IFNAR2*^–/–^ *MORC3*^*AIDV5/AIDV5*^ TIR1^F74G^ BLaER1 monocytes treated with 5Ph-IAA for the indicated time or DMSO for 48 h. Data represent the mean + SEM from *n* = 3 independent experiments or one representative blot of two. ***P < 0.001; **P < 0.01; ns, not significantly different as determined by one-way ANOVA and Bonferroni’s post hoc test. *IFNB1*: 1 h vs DMSO, *P* = 0.8117; 2 h vs DMSO, *P* = 0.002; all others, *P* < 0.0001. *MLLT3*: 1 h and 2 h vs DMSO, *P* > 0.9999; 4 h vs DMSO, *P* = 0.7873; all others, *P* < 0.0001. (**D**) Read-density heatmap showing the normalized coverage of anti-V5 (MORC3) ChIP-seq and anti-H3K4me3 Cut&Run signal at a 5 kb window centered around MORC3-V5 peaks from *IFNAR1*^–/–^ *IFNAR2*^–/–^ *MORC3*^*AIDV5/AIDV5*^ TIR1^F74G^ BLaER1 monocytes. (**E**) Differential chromatin accessibility determined by ATAC-seq at MORC3-V5 peaks comparing *IFNAR1*^–/–^ *IFNAR2*^–/–^ and *IFNAR1*^–/–^ *IFNAR2*^–/–^ *MORC3*^–/–^ BLaER1 monocytes from *n* = 3 independent experiments. Data is from GSE183011. MORC3 ChIP-seq peaks that overlap with tandem repeats are highlighted in orange. *P* values were calculated as part of DESeq2 using the Wald test and adjusted for multiple testing (*P*_adj_) using the Benjamini–Hochberg method. Adjusted *P* values were capped at 10^−10^ for visualization. (**F**) Genome browser view of MORC3 binding, ATAC-seq signal, and simple repeats track for selected tandem repeat regions. Source data are available online for this figure.

**Figure EV1:**
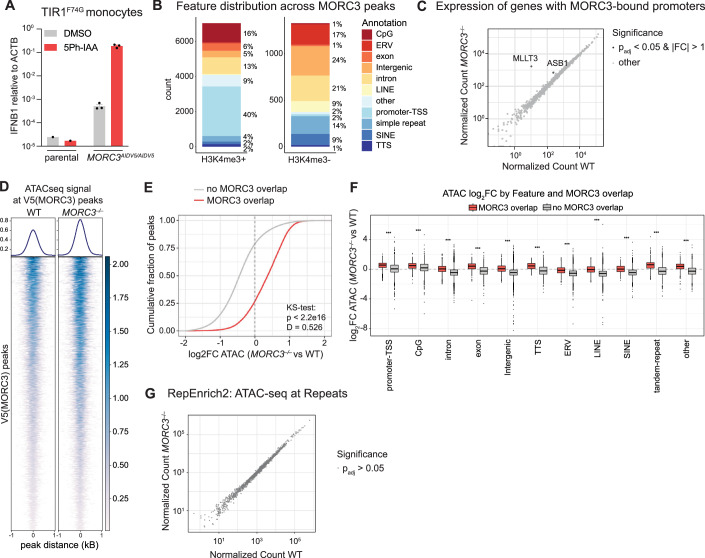
MORC3 does not repress gene expression in monocytes at bound promoters. (**A**) *IFNB1* gene expression in *IFNAR1*^–/–^ *IFNAR2*^–/–^ TIR1^F74G^ or *IFNAR1*^–/–^ *IFNAR2*^–/–^ *MORC3*^*AIDV5/AIDV5*^ TIR1^F74G^ BLaER1 monocytes (parental or *n* = 3 independent monoclones) treated with 5Ph-IAA or DMSO for 24 h. (**B**) Genomic feature annotation at MORC3-V5 peaks stratified into H3K4me3 overlapping and non-overlapping groups. Relative abundance was rounded to the next integer and is shown for features ≥1%. (**C**) Expression of genes with MORC3-bound promoters in BLaER1 monocytes comparing WT and *MORC3*^–/–^ conditions (*n* = 10). Data from GSE183011 included *IFNAR1*^–/–^ *IFNAR2*^–/–^, *IFNB1*^–/–^, Cas9 *STAT1*^–/–^ *STAT2*^–/–^ and Cas9 *STAT1*^–/–^ *STAT2*^–/–^ MRE^–/–^ monocytes, and *MORC3*^–/–^ matched conditions. *MLLT3* is in the vicinity of the IFNB1-MRE, and *ASB1* is in the vicinity of the TWIST2-MRE. *P* values were calculated as part of DESeq2 using the Wald test and adjusted for multiple testing (*P*_adj_) using the Benjamini–Hochberg method. (**D**) Read-density heatmap of ATAC-seq signal at MORC3-V5 peaks comparing *IFNAR1*^–/–^ *IFNAR2*^–/–^ and *IFNAR1*^–/–^ *IFNAR2*^–/–^ *MORC3*^–/–^ BLaER1 monocytes. (**E**, **F**) Cumulative differential analysis and differential analysis at annotated genomic feature regions of ATAC-seq signal at ATAC-seq peaks overlapping or non-overlapping with MORC3-V5 peaks comparing *IFNAR1*^–/–^ *IFNAR2*^–/–^ and *IFNAR1*^–/–^ *IFNAR2*^–/–^ *MORC3*^–/–^ BLaER1 monocytes. Data is from GSE183011. Data were analyzed by the Kolmogorov–Smirnov (KS) test in (**E**) or the Wilcoxon rank-sum test and Bonferroni’s post hoc test in (**F**). Promoter-TSS, *P* = 8.88E-264; CpG, *P* = 8.63E-30, intron, *P* = 0.00E + 00; exon, *P* = 5.29E-70; Intergenic, *P* = 0.00E + 00; TTS, *P* = 4.37E-46; ERV, *P* = 3.45E-78; LINE, *P* = 4.09E-88; SINE, *P* = 4.36E-37; tandem-repeat, *P* = 4.62E-165; other, *P* = 3.68E-170. The center line of the boxplots in (**F**) indicates the median (50th percentile). The box bounds mark the first (Q1, 25th percentile) and third quartiles (Q3, 75th percentile). The whiskers extend to the most extreme data points within 1.5 times the interquartile range (IQR  =  Q3–Q1) from the quartiles. (**G**) Differential analysis of ATAC-seq signal at interspersed repeat elements using Repenrich2 comparing *IFNAR1*^–/–^ *IFNAR2*^–/–^ and *IFNAR1*^–/–^ *IFNAR2*^–/–^ *MORC3*^–/–^ BLaER1 monocytes. Data are from GSE183011. *P* values were calculated as part of DESeq2 using the Wald test and adjusted for multiple testing (*P*_adj_) using the Benjamini–Hochberg method. Unless otherwise indicated, data are from *n* = 3 independent experiments. Source data are available online for this figure.

To investigate how MORC3 specifically represses the noncanonical IFN pathway, we utilized human BLaER1 monocytes, an established in vitro model for dissecting human inflammatory responses (Gaidt et al, 2016; Rapino et al, 2013). To achieve precise temporal control over MORC3 depletion and to generate a tool for MORC3-affinity purification, we endogenously tagged MORC3 with a C-terminal auxin-inducible degron (AID)-V5 sequence (Fig. 1A). This system enables rapid protein degradation upon addition of the auxin derivative 5-phenyl-indole-3-acetic acid (5Ph-IAA), mediated by the transgenic *Oryza sativa*-derived TIR1(F74G) protein (Yesbolatova et al, 2020). Tagging induced a tenfold de-repression of IFN in the absence of 5Ph-IAA, suggesting a hypomorphic activity due to the tag (Fig. EV1A). However, rapid MORC3 depletion upon 5Ph-IAA treatment (Fig. 1B) induced 1000-fold expression of *IFNB1* (Fig. 1C), showing that *MORC3*^*AIDV5/AIDV5*^ BLaER1 monocytes provide a robust model for investigating MORC3’s anti-inflammatory function. Expression of *MLLT3*, another MRE-adjacent gene that is similarly upregulated upon loss of MORC3 (Gaidt et al, 2021b), was also induced, albeit with slightly slower kinetics (Fig. 1C). To identify genomic elements directly bound by MORC3, we performed anti-V5 chromatin immunoprecipitation followed by sequencing (ChIP-seq) in *MORC3*^*AIDV5/AIDV5*^ monocytes and identified 8294 MORC3 peaks consistent across biological replicates. In line with previous reports of MORC3 binding to promoters (Li et al, 2016), 7039 (85%) MORC3 peaks were enriched for H3K4me3 and 40% of these were annotated as promoters (Figs. 1D and EV1B). This binding pattern is consistent with the known interaction between MORC3’s CW-Zinc finger domain and H3K4me3, which facilitates binding of the ATPase domain to DNA (Zhang et al, 2019). Some sites with strong MORC3 binding did not show a strong H3K4me3 signal, suggesting that additional H3K4me3-independent recruitment mechanisms may exist.

**Figure EV2:**
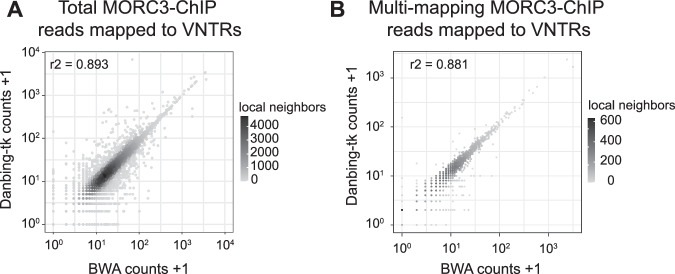
Comparison of BWA and Danbing-tk in mapping short reads to VNTRs. Total **(A)** or multi-mapping **(B)** anti-V5 (MORC3) ChIP-seq reads from a single replicate were mapped to VNTRs using the conventional BWA aligner or Danbing-tk, a computational tool specialized in assigning reads to VNTRs (see “Methods”). Source data are available online for this figure.

To pinpoint genomic sites actively repressed by MORC3, we first examined the expression of genes with MORC3-bound promoters. Transcriptomic analysis of *MORC3*^*–/–*^ monocytes showed no broad de-repression of genes with MORC3-bound promoters (Fig. EV1C), indicating that MORC3 binding is not sufficient to establish transcriptional repression at promoters. We therefore assessed which MORC3-bound sites gained DNA accessibility in *MORC3*^*–/–*^ monocytes. Cumulative analysis showed that MORC3-bound regions exhibit a general increase in accessibility in the absence of MORC3 (Fig. EV1D,E), regardless of genomic feature (Fig. EV1F). However, accessibility changes were mostly subtle, including at ERVs (Fig. EV1G), and only 172 sites displayed a significant gain in accessibility upon MORC3 loss (log_2_FC > 1 and *P*_adj_ < 0.05) (Fig. 1E). Notably, of these 172 sites, 12% were tandem repeats (21 sites) compared to only 5% tandem repeats within other MORC3-bound loci. Additionally, 6/7 of the most strongly affected sites (log_2_FC > 2 and *P*_adj_ < 0.05) were tandem repeats (Fig. 1E,F). Tandem repeats are repetitive genomic segments generated by duplication events, distinct from interspersed repeats of viral or transposable origin (Gemayel et al, 2010; Wells and Feschotte, 2020). The consensus repeat unit of accessibility restricted tandem repeats ranged from 20 to 192 bp, indicating that they are not composed of short tandem repeats (STRs, also called microsatellites) but instead can be classified as variable number tandem repeats (VNTRs, also called mini-satellites). Because the repetitiveness of tandem repeats presents a challenge for standard short-read data, we validated our BWA-mapping strategy using Danbing-tk (Lu et al, 2021), a computational method, which employs a de Bruijn graph-based representation of tandem repeats for faithful read assignment (Fig. EV2; see “Methods”). Collectively, these results demonstrate that MORC3 binds to many genomic sites that are often marked by H3K4me3 but does not generally repress transcription or limit chromatin accessibility strongly at bound sites. Only a small number of loci—enriched for tandem repeats—show pronounced accessibility increase upon MORC3 loss.

### A tandem repeat is a MORC3-repressed enhancer of IFNB1

**Figure 2:**
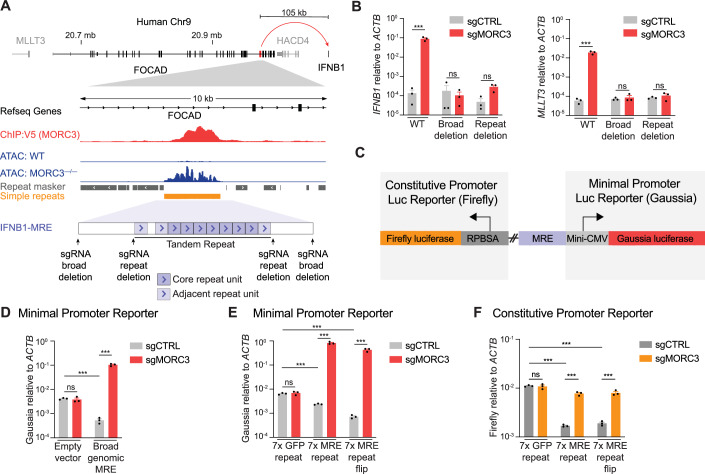
A MORC3-repressed tandem repeat is an enhancer of *IFNB1* expression upon loss of MORC3. (**A**) The human MRE locus lies within an intron of *FOCAD* and induces expression of the *IFNB1* gene upon loss of MORC3. ATAC-seq signal from BLaER1 monocytes (*IFNAR1*^–/–^ *IFNAR2*^–/–^ as WT and *IFNAR1*^–/–^ *IFNAR2*^–/–^ *MORC3*^–/–^; GSE183011), anti-MORC3-V5 ChIP-seq signal (peak 22339 in Fig. 1E) and UCSC browser tracks are shown. sgRNAs used to make the previous broad deletion and targeted repeat deletion are shown. MRE = MORC3-repressed element. (**B**) The remaining MRE allele in Cas9 *STAT1*^–/–^ *STAT2*^–/–^ MRE^–/+^ BLaER1 monocytes was edited to induce a broad deletion or a specific repeat deletion using sgRNAs from (**A**). Gene expression upon lentiviral sgRNA delivery is depicted. *IFNB1*: WT, *P* < 0.0001; Broad deletion, *P* > 0.9999; Repeat deletion, *P* = 0.5297. *MLLT3*: WT, *P* < 0.0001; all others, *P* > 0.9999. (**C**) The dual luciferase MRE reporter is integrated into the genome of monocytes using transposase. The Gaussia luciferase is driven by a minimal CMV promoter and reports activation of the adjacent MRE upon loss of MORC3. The Firefly luciferase is driven by a constitutive RPBSA promoter and reports repression by the adjacent MRE in WT cells. (**D**) The broad MRE sequence was amplified from genomic DNA of BLaER1 monocytes, inserted into the luciferase reporter, and integrated into the genome of Cas9 *IFNAR1*^–/–^ *IFNAR2*^–/–^ MRE^–/–^ BLaER1 cells. Minimal Promoter Gaussia luciferase gene expression upon lentiviral sgRNA delivery is depicted. ns, *P* > 0.9999; ***P < 0.0001. (**E**, **F**) Seven concatenated consensus IFNB1-MRE repeat units, or control GFP-derived repeat units of the same length, were inserted into the luciferase reporter and integrated into the genome of Cas9 *IFNAR1*^–/–^ *IFNAR2*^–/–^ MRE^–/–^ BLaER1 cells. Minimal Promoter Gaussia luciferase (**E**) or Constitutive Promoter Firefly luciferase (**F**) gene expression upon lentiviral sgRNA delivery is depicted. The “flip” condition contains the same IFNB1-MRE consensus repeat sequence in reverse orientation. ns, *P* > 0.99; ***P < 0.0001. Unless otherwise indicated, data represent the mean + SEM from *n* = 3 independent experiments. ***P < 0.001; ns, not significantly different as determined by two-way ANOVA and Bonferroni’s post hoc test. Source data are available online for this figure.

**Figure EV3:**
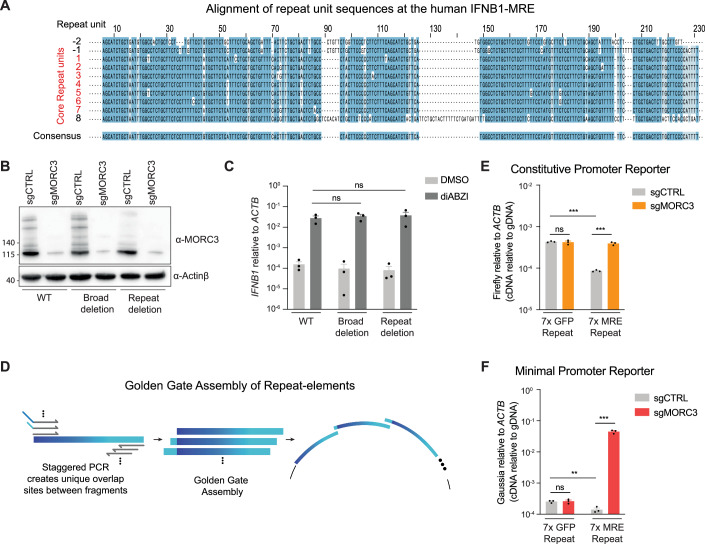
The IFNB1-controlling MRE tandem repeat. (**A**) The consensus sequence of the repeat units at the MRE locus of the hg38 reference genome was determined using the Tandem Repeats Finder algorithm. Sequences of individual repeat units were aligned to the consensus sequence using Clustal Omega. Core repeat units with >95% identity to the consensus sequence are in red. MRE = MORC3-repressed element. (**B**, **C**) The remaining MRE allele in Cas9 *STAT1*^*–/–*^ *STAT2*^*–/–*^ MRE^–/+^ BLaER1 monocytes was edited to induce a broad deletion or a specific repeat deletion. One representative immunoblot of two upon lentiviral sgRNA delivery and *IFNB1* expression after stimulation with the STING agonist diABZI is shown. Ns, *P* > 0.9999. (**D**) Golden Gate repeat element assembly. Staggered PCR strategy using a synthetic consensus/mutant variant as template generates unique sticky ends between fragments. (**E**, **F**) Seven concatenated consensus IFNB1-MRE repeat units, or control GFP-derived repeat units of the same length, were inserted into the luciferase reporter and integrated into the genome of Cas9 *IFNAR1*^–/–^ *IFNAR2*^–/–^ MRE^–/–^ BLaER1 cells. Constitutive Promoter Firefly luciferase and Minimal Promoter Gaussia luciferase gene expression upon lentiviral sgRNA delivery was determined by qPCR on reverse transcribed cDNA and normalized to qPCR on genomic DNA. **P = 0.0079; ***P < 0.0001; ns, *P* > 0.9999. Data are shown as mean + SEM from *n* = 3 independent experiments. Significance of differences was determined by two-way ANOVA and Bonferroni’s post hoc test. Source data are available online for this figure.

Next, we wondered if restricting accessibility at tandem repeats can explain how MORC3 specifically represses the noncanonical IFN-pathway. Previously, we have identified a cis-regulatory element termed the MORC3-regulated element (MRE), located within an intron of the *FOCAD* gene ca. 100 kb downstream of *IFNB1*, which is essential for *IFNB1* induction upon MORC3 loss. This element was initially characterized as MORC3-regulated due to increased chromatin accessibility specifically in *MORC3*^*–/–*^ monocytes (Fig. 2A) (Gaidt et al, 2021b). Intriguingly, increased accessibility upon loss of MORC3 completely overlaps with a MORC3-bound tandem repeat (Fig. 2A). The repeat is composed of seven core direct repeat units, each 192 bp in length and over 95% identical to a consensus sequence, along with three additional repeat units sharing ~80% similarity (Figs. 2A and EV3A, Peak 22339 in Fig. 1E). The consensus sequence itself is an imperfect dimer. The locus is annotated as a simple tandem repeat by the tandem repeat finder algorithm (Benson, 1999) (orange simple repeat track in Fig. 2A) and is not derived from retroviral or transposable elements (Bao et al, 2015) (gray repeat masker track in Fig. 2A). Collectively, the repeat constitutes 58% of the MRE region that we previously defined by the boundaries of its genetic loss-of-function deletion (Fig. 2A). Deletion of the repeat region alone abrogated induction of *IFNB1* and *MLLT3* by loss of MORC3 (Figs. 2B and EV3B) but not by the STING-agonist diABZI (Fig. EV3C), suggesting that the repeat mediates IFN induction specifically in the MORC3-MRE pathway. To test the gene-regulatory functions of the MRE outside of its genomic context, we devised a reporter assay based on a Mini-CMV promoter-driven Gaussia luciferase gene (Minimal Luc) and a RPBSA promoter-driven Firefly luciferase gene (Constitutive Luc) (Fig. 2C). This reporter is integrated into the genome of BLaER1 cells using the Sleeping Beauty transposase system. The broad genomic MRE sequence, amplified from genomic DNA of BLaER1, activated the Minimal Luc reporter upon loss of MORC3 (Fig. 2D), suggesting that the broad MRE is sufficient to drive gene expression in the absence of MORC3 and establishing the MRE as a repressed enhancer that also functions outside of its genomic context. Next, we aimed to establish an in vitro cloning system to dissect the function of the tandem repeat. Repetitive DNA elements are challenging to engineer in vitro because the repetitiveness hinders targeted amplification and manipulation. To overcome this, we developed a Golden Gate strategy to assemble repeat variants from staggered PCR products (Fig. EV3D). As a proof-of-concept, we compared an artificial wild-type MRE, constructed by concatenating seven core consensus repeats, to a control repeat derived from a partial GFP-coding sequence of the same size and repetitiveness. Following MORC3 depletion, only the consensus MRE repeat, but not the control GFP repeat showed enhancer activity toward the Minimal Luc reporter comparable in magnitude to the broad genomic MRE sequence (Fig. 2E). The orientation of the MRE repeat within the reporter had no effect on reporter activation (Fig. 2E). This demonstrates that the repeat is sufficient for driving gene expression in the absence of MORC3. We initially included the Constitutive Luc reporter in our design for normalization purposes; however, we observed MORC3-dependent repression of the Constitutive Luc reporter when positioned adjacent to the MRE repeat, but not when adjacent to the control repeat (Fig. 2F), indicating that MORC3-mediated repression can spread to this adjacent constitutively expressed reporter gene. Normalization by genomic qPCR confirmed that this repression was genuinely mediated by the MRE (Fig. EV3E), ruling out differences in genomic integration efficiency as the cause of the lower Constitutive Luc Reporter signal. Consistent with this repressive activity of the MRE repeat, also the Minimal Promoter Reporter showed slightly less baseline activity if placed next to an MRE repeat (Figs. 2D,E and EV3F). In summary, our results demonstrate MORC3 specifically represses an *IFNB1*-controlling tandem repeat element: under steady-state conditions, the repeat is repressed and upon loss of MORC3 it is converted into a potent enhancer of *IFNB1*.

### MORC3 recruits DAXX for H3.3 incorporation and MRE repression

**Figure EV4:**
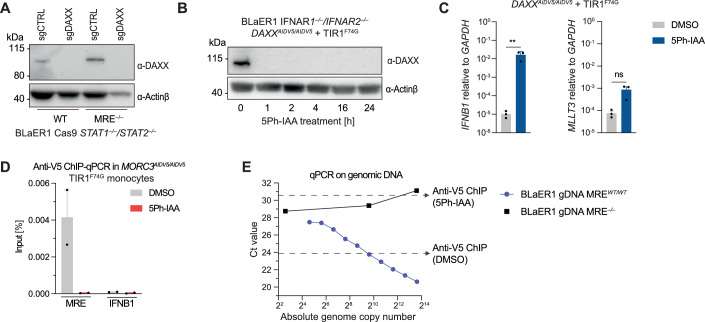
Validation of DAXX deletion and ChIP-qPCR at the MRE. (**A**, **B**) Immunoblot analysis in BLaER1 monocytes of the indicated genotype after lentiviral delivery of sgRNAs or treatment with 5Ph-IAA to degrade DAXX. One representative immunoblot of two is shown. (**C**) Gene expression analysis in *IFNAR1*^–/–^ *IFNAR2*^–/–^ *DAXX*^*AIDV5/AIDV5*^ TIR1^F74G^ BLaER1 monocytes after treatment with 5Ph-IAA for 24 h. Data is shown as mean + SEM from *n* = 3 independent experiments. **P = 0.0062; ns, *P* = 0.0723 as determined by paired, two-sided *t* test. (**D**) Anti-V5 ChIP-qPCR analysis in *IFNAR1*^–/–^ *IFNAR2*^–/–^ *MORC3*^*AIDV5/AIDV5*^ TIR1^F74G^ BLaER1 monocytes that were treated with 5Ph-IAA for 24 h is depicted as mean + SEM of *n* = 2 independent experiments. The IFNB1 locus (gene body) serves as a negative control where MORC3 does not bind. (**E**) Linearity and dynamic range of qPCR-based quantification of MRE-repeat DNA. Ct values from qPCR with purified genomic DNA from WT or MRE^–/–^ BLaER1 cells are depicted from one experiment. Arrows indicate raw Ct values from the anti-V5 ChIP-qPCR experiment in (**D**). Source data are available online for this figure.

**Figure 3:**
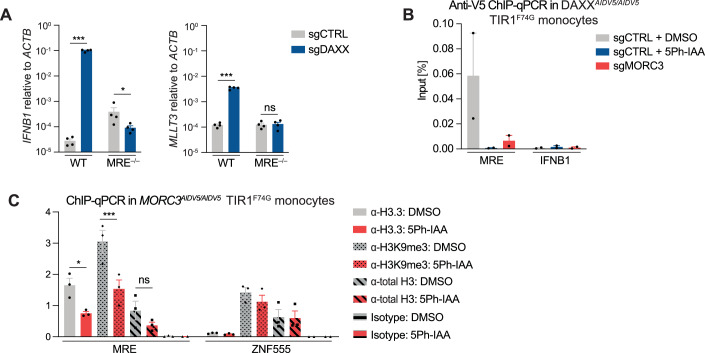
MORC3 recruits DAXX to silence the MRE via incorporation of H3.3. (**A**) Gene expression analysis in Cas9 *STAT1*^–/–^ *STAT2*^–/–^ BLaER1 monocytes of the indicated genotype upon lentiviral sgRNA delivery measured in *n* = 4 independent experiments. ***P < 0.0001; *P = 0.0253; ns, *P* > 0.9999. (**B**) Anti-V5 ChIP-qPCR analysis in *IFNAR1*^–/–^ *IFNAR2*^–/–^ *DAXX*^*AIDV5/AIDV5*^ TIR1^F74G^ BLaER1 monocytes upon lentiviral delivery of sgRNAs and treatment with 5Ph-IAA for 24 h to degrade DAXX measured in *n* = 2 independent experiments. The IFNB1 locus (gene body) serves as a negative control where DAXX does not bind. (**C**) ChIP-qPCR analysis in Cas9 *IFNAR1*^–/–^ *IFNAR2*^–/–^ *MORC3*^*AIDV5/AIDV5*^ TIR1^F74G^ BLaER1 monocytes treated with 5Ph-IAA for 24 h and measured in *n* = 3 independent experiments. The ZNF555 locus serves as a negative control where no MORC3-dependent change is expected. *P = 0.0176; ***P < 0.0001; ns, *P* > 0.9999. Statistical significance was determined by two-way ANOVA and Bonferroni’s post hoc test. Source data are available online for this figure.

To dissect how MORC3 represses the MRE, we explored the involvement of DAXX, a chaperone for the histone variant H3.3. MORC3 is known to recruit DAXX to deposit H3.3 and establish H3K9me3 at ERVs to mediate silencing in mouse embryonic stem cells (Groh et al, 2021). Genetic deletion of *DAXX* in BLaER1 monocytes (Fig. EV4A) phenocopied loss of MORC3, leading to strong induction of *IFNB1* and the adjacent MRE-regulated gene *MLLT3*, and this induction required the MRE (Fig. 3A). We also introduced a C-terminal AID-V5 tag in the endogenous *DAXX* locus to allow rapid protein degradation and affinity purification of endogenous DAXX (Fig. EV4B). Rapid AID-mediated DAXX protein degradation resulted in a similar de-repression of *IFNB1* and *MLLT3* (Fig. EV4C). To test whether MORC3 recruits DAXX to the MRE, we established a ChIP–qPCR assay for this repetitive locus that faithfully detects MORC3 binding (Fig. EV4D) within the linear range of detection (Fig. EV4E). Using this assay, we found that MORC3 recruits DAXX to the MRE (Fig. 3B) and that MORC3 is required for both H3.3 incorporation and H3K9me3 levels at this site (Fig. 3C). The reduced H3K9me3 signal coincided with a decrease in total H3 upon MORC3 loss. Together, these findings demonstrate that in human monocytes, the MRE is repressed through a MORC3–DAXX–H3.3 axis. Having identified genetic components of this repression, we will from now on refer to this locus as the MORC3-repressed element instead of the previously coined term MORC3-regulated element.

### Massive accumulation of SPI-transcription factor motifs at the MRE

**Figure 4:**
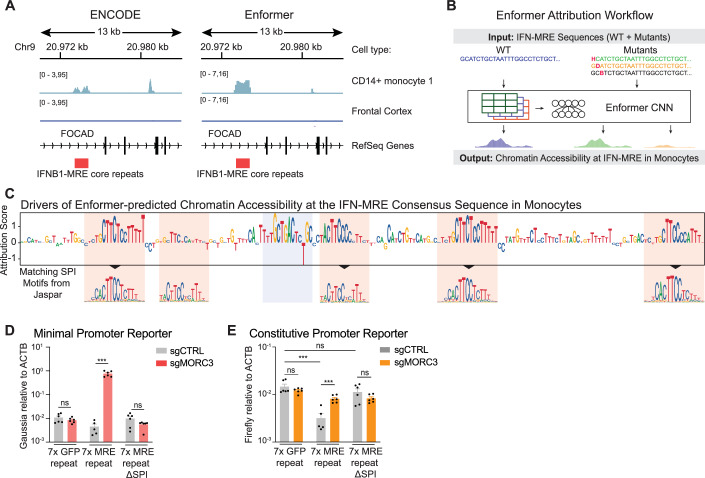
A large SPI motif cluster within the MRE-repeat is necessary for both enhancer function and repression by MORC3. (**A**) Genome browser view of experimental (ENCODE) or Enformer-predicted chromatin accessibility (DNase hypersensitivity) at the MRE in two different cell types. (**B**) Attribution analysis of Enformer predictions reveals which nucleotides drive chromatin accessibility. Every position of the MRE consensus sequence is mutated, and chromatin accessibility is predicted. Decreased chromatin accessibility of the mutant compared to the wild-type sequence indicated positions that mediate accessibility. (**C**) Importance of each nucleotide of the MRE consensus sequence for the Enformer-predicted chromatin accessibility in monocytes. Spans of nucleotides with high attribution scores matching SPI motifs in the Jaspar database are highlighted in red, a MAF motif is highlighted in blue. (**D**, **E**) All SPI motifs in the 7× consensus MRE were replaced with random nucleotides, and the construct was integrated as a luciferase reporter into the genome of Cas9 *IFNAR1*^–/–^ *IFNAR2*^–/–^ MRE^–/–^ BLaER1 cells. Minimal Promoter Gaussia luciferase (**D**) or Constitutive Promoter Firefly luciferase (**E**) gene expression upon lentiviral sgRNA delivery is depicted as mean + SEM from *n* = 6 independent experiments. Missing datapoints are non-detected values in qPCR. ***P ≤ 0.0001; ns, *P* > 0.9999 as determined by two-way ANOVA and Bonferroni’s post hoc test. Source data are available online for this figure.

**Figure EV5:**
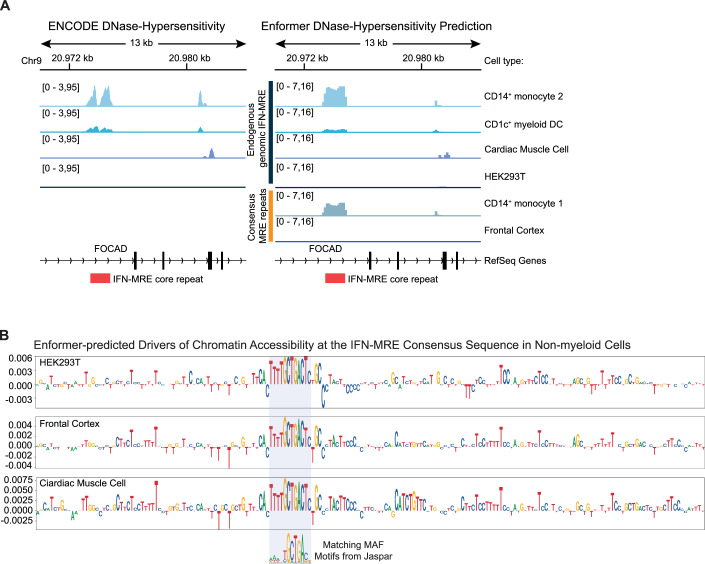
The deep learning sequence-to-function model Enformer predicts cell type-specific accessibility at the MRE in monocytes. (**A**) ENCODE DNase hypersensitivity tracks and Enformer-predicted DNase hypersensitivity tracks of indicated cell types at the human genomic MRE. Additionally, the core tandem repeat units at the human MRE locus were replaced with the 7x consensus repeat sequence, and DNase hypersensitivity in indicated cell types was predicted using Enformer. (**B**) Importance of each nucleotide of the MRE consensus sequence for the Enformer-predicted chromatin accessibility in non-myeloid cells. Spans of nucleotides with high attribution scores matching a MAF motif in the Jaspar database are highlighted in blue. Since there is no chromatin accessibility at this locus in these cells, these predictions can be considered background. Source data are available online for this figure.

We next sought to dissect the enhancer activity of the MRE and employed the deep learning sequence-to-function neural network Enformer (Avsec et al, 2021a) to reveal sequence features that drive enhancer activity. Enformer was trained on ENCODE datasets of different genomics assays (i.e., chromatin accessibility by DNase-hypersensitivity) in various cell types. Consistent with its training data, Enformer predicted cell-type-specific chromatin accessibility at the MRE in monocytes, but not in other cell types such as frontal cortex tissue or HEK293T cells (Figs. 4A and EV5A). This is consistent with our previous observations that MORC3 deletion induces *IFNB1* in monocytes but not in other cell lineages (Gaidt et al, 2021b). To determine the contribution of each nucleotide to this accessibility, we performed an attribution analysis by individually mutating each position of the consensus MRE repeat in silico and evaluating the changes in the chromatin accessibility prediction (Fig. 4B). If mutating a position led to a lower predicted accessibility, this position was considered to be a driver of accessibility. Remarkably, the nucleotides that were predicted to drive chromatin accessibility constituted four erythroblast transformation-specific (ETS) motifs per repeat (Fig. 4C). Manual inspection revealed a fifth occurrence of the same motif, totaling 35 motifs across the seven-repeat sequence. Considering the three adjacent repeat units with varying sequence similarity (Fig. EV3A), the human reference genome contains 45 SPI motifs at the MRE. Specifically, these motifs showed the highest similarity to transcription factor motifs of the spleen focus-forming virus proviral integration oncogene (SPI) subfamily of ETS-transcription factors, and we will therefore refer to these motifs as SPI motifs. A single v-maf avian musculoaponeurotic fibrosarcoma oncogene homolog (MAF)-motif (blue in Fig. 4C) was not considered as the driver of monocyte-specific chromatin accessibility, because it was also predicted in other cell lineages (Fig. EV5B). Mutation of all SPI motifs abolished MRE-driven Minimal Luc reporter expression in MORC3-deleted cells (Fig. 4D), confirming the requirement of these motifs for enhancer activity. Strikingly, these motifs were also required for repeat-mediated repression of the Constitutive Luc reporter (Fig. 4E). Thus, both functions of the MRE, being repressive in wild-type cells and a potent enhancer upon MORC3 loss, are mediated through SPI transcription factor binding motifs, which are massively accumulated at this locus.

### Large SPI motif clusters are sufficient for repressed enhancer activity

**Figure 5:**
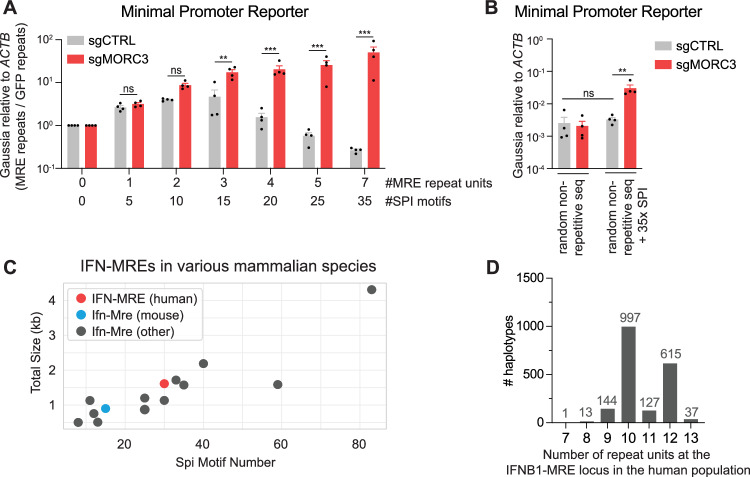
Large SPI motif clusters are sufficient to be repressed by MORC3 and are the evolutionarily conserved features of IFN-MREs. (**A**) Increasing numbers of MRE-repeat units or GFP-repeat units were inserted into the luciferase reporter and integrated into the genome of Cas9 *IFNAR1*^–/–^ *IFNAR2*^–/–^ MRE^–/–^ BLaER1 cells. Minimal Promoter Gaussia luciferase gene expression upon lentiviral sgRNA delivery is depicted. Data is normalized to GFP repeats. 0 represents the empty vector control. ***P < 0.001; ns, *P* > 0.9999 unless 3:sgCTRL vs. 3:sgMORC3, *P* = 0.0011. (**B**) 35 SPI motifs were integrated into a random non-repetitive sequence and integrated as luciferase reporter into the genome of Cas9 *IFNAR1*^–/–^ *IFNAR2*^–/–^MRE^–/–^ BLaER1 cells. Minimal Promoter Gaussia luciferase gene expression upon lentiviral sgRNA delivery is depicted. Ns, *P* > 0.9999; **P = 0.0024. (**C**) Mres in different mammalian species. Mres are tandem repeats with Spi motifs in an intron of the *Focad* gene near an *Ifn* gene. Tandem repeats were identified with the Tandem Repeats Finder algorithm and searched for occurrences of Spi transcription factor motifs from the Jaspar database using FIMO. (**D**) Number of tandem repeat units at the human MRE-VNTR from haplotypes from the 1000 genomes project (VNTR = variable number of tandem repeats). Data represent the mean + SEM from *n* = 4 independent experiments. Statistical significance was determined by two-way ANOVA and Bonferroni’s post hoc test. Source data are available online for this figure.

**Figure EV6:**
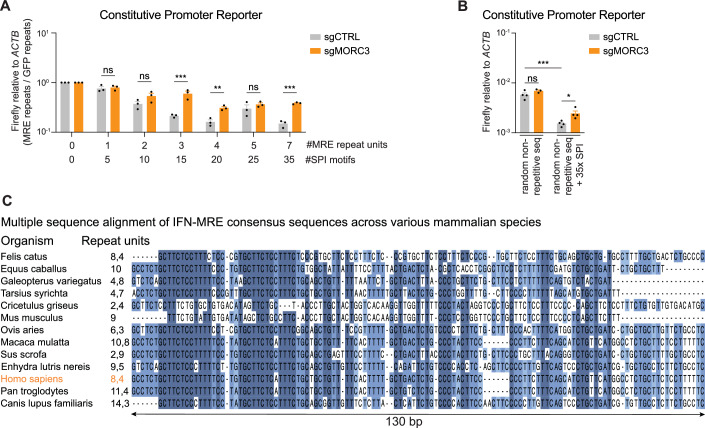
MORC3 represses SPI motif clusters. (**A**) Increasing numbers of MRE-repeat units or GFP-repeat units were inserted into the luciferase reporter and integrated into the genome of Cas9 *IFNAR1*^–/–^ *IFNAR2*^–/–^MRE^–/–^ BLaER1 cells. Constitutive Promoter Firefly luciferase gene expression upon lentiviral sgRNA delivery is depicted. Data is normalized to GFP repeats. 0 represents the empty vector control. Data represent the mean + SEM, *n* = 3 independent experiments. 1, *P* > 0.9999; 2, *P* = 0.1404; 3, *P* < 0.0001; 4, *P* = 0.0011; 5, *P *= 0.8596; 7, *P* < 0.0001. ***P < 0.001; **P < 0.01; ns, not significantly different. (**B**) 35 SPI motifs were integrated into a random non-repetitive sequence and integrated as luciferase reporter into the genome of Cas9 *IFNAR1*^–/–^ *IFNAR2*^–/–^ MRE^–/–^ BLaER1 cells. Constitutive Promoter Firefly luciferase gene expression upon lentiviral sgRNA delivery is shown. Data represent the mean + SEM, *n* = 4 independent experiments. ***P < 0.0001; *P = 0.015; ns, *P* = 0.9562. (**C**) Multiple-sequence alignment of consensus sequences from Mres of various species. Mres are tandem repeats enriched in Spi transcription factor binding sites in an intron of the *Focad* gene near an *Ifn* gene. Significance of differences was determined by two-way ANOVA and Bonferroni’s post hoc test. Source data are available online for this figure.

To dissect repressive and enhancer activities of repeat units containing SPI motif clusters, we systematically assessed how incremental numbers of MRE repeat units corresponding to 5, 10, 15, 20, 25, or 35 SPI motifs impact these gene-regulatory activities. At lower motif numbers, a mild activation was observed in both wild-type and MORC3-deleted cells (Fig. 5A), suggesting that low motif numbers support enhancer activity without effective repression. Notably, MORC3-mediated repression only emerged at motif clusters containing >15 motifs, leading to a decrease of reporter signal in wild-type cells (Fig. 5A). Unabated activation in MORC3-deleted cells increased exponentially with the number of SPI motifs, indicating synergistic enhancer activity at large motif clusters. The Constitutive Luc reporter was repressed by higher numbers of SPI motifs in MRE repeats compared to control repeats of similar length and repetitiveness, in a MORC3-dependent manner (Fig. EV6A). In conclusion, repeat unit/motif accumulation dictates both repressive and enhancer functions of the MRE. High repeat unit numbers giving rise to high motif numbers allow repression during steady state and synergistic activation upon MORC3 deletion, while few repeat units/motifs mediate weak enhancer activation. To directly test if a large SPI motif cluster is sufficient to create a MORC3-repressed enhancer, we randomly inserted 35 SPI motifs from the MRE into a non-repetitive random DNA sequence. This construct specifically activated a Minimal Luc reporter in MORC3-deficient but not in wild-type cells (Fig. 5B) and induced MORC3-mediated repression of the Constitutive Luc reporter (Fig. EV6B). In both cases, the activity was weaker compared to the consensus repeat sequence, suggesting that additional structural or sequence features in the endogenous tandem repeat might enhance these gene-regulatory activities. Together, these findings demonstrate that large SPI motif clusters are sufficient to create a MORC3-repressed enhancer.

### SPI motif accumulation is a conserved feature of IFN-MREs in different species and individuals

Investigating the evolutionary conservation of the MRE locus, we identified tandem repeats with large Spi motif clusters within introns of the *Focad* gene near Ifn loci across diverse mammalian species (Dataset EV1). Although these repeats varied in overall length, repeat unit size, and consensus sequences, they uniformly featured a large cluster of Spi transcription factor binding sites, ranging from 10 to 80 motifs (Figs. 5C and EV6C; Dataset EV1). Thus, the exceptional density of Spi motifs within a large cluster is an evolutionarily conserved characteristic of this genomic locus. Next, we investigated the evolutionary variability of the MRE locus within the human population. Tandem repeats are known to be inherently unstable, frequently expanding or contracting through replication slippage during germline DNA replication and unequal crossing-over events, contributing substantially to inter-individual genetic diversity (Gemayel et al, 2010). Analysis of human MRE variants in the 1000 Genomes dataset showed variability in the number of repeats between individuals, ranging from 7 to 13 repeats (Fig. 5D). This variability in repeat number may contribute to differences in *IFNB1*-induction between individuals because enhancer activity scales exponentially with higher repeat numbers in reporter assays (Fig. 5A). Notably, no allele in the 1000 genomes dataset had less than 7 repeats (Fig. 5D) which is sufficient for MORC3-mediated repression (Figs. 5A and EV6A), supporting the conservation of motif cluster-mediated repression of the MRE. In conclusion, the high-density Spi motif cluster within a tandem repeat is an evolutionarily conserved feature of MREs. However, inter-individual variability in repeat numbers may underpin differences in IFNB1 induction in the human population.

### Motif clusters of several transcription factors at MORC3-restricted tandem repeats

**Figure EV7:**
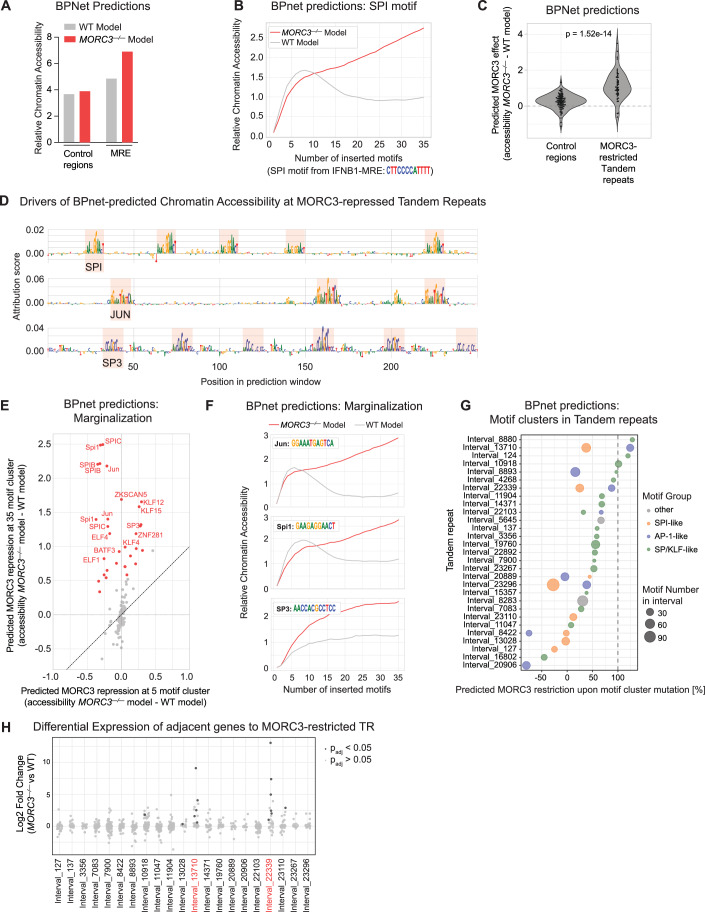
BP-net sequence-to-function deep learning models predict motif clusters of various transcription factors to cause MORC3-limited accessibility at tandem repeats. (**A**) BPNet models were trained on ATAC-seq data from *IFNAR1*^–/–^ *IFNAR2*^–/–^ *MORC3*^–/–^ or *IFNAR1*^–/–^ *IFNAR2*^–/–^ BLaER1 monocytes. Predicted accessibility at control regions or the MRE is shown. Data for control regions represents the average prediction value of 100 random genomic sequences. (**B**) In all, 1–35 SPI motifs were inserted into random genomic sequences, and chromatin accessibility was predicted using BPNet models that were trained on ATAC-seq data from *IFNAR1*^–/–^ *IFNAR2*^–/–^ and *IFNAR1*^–/–^ *IFNAR2*^*–/–*^ *MORC3*^*–/–*^ BLaER1 monocytes. (**C**) MORC3-bound and -restricted tandem repeats were analyzed with the BPNet model trained on ATAC-seq data from *IFNAR1*^–/–^ *IFNAR2*^–/–^ *MORC3*^–/–^ or *IFNAR1*^–/–^ *IFNAR2*^–/–^ BLaER1 monocytes. The MORC3 effect was calculated by subtracting the predicted accessibility of the wild-type model from the prediction of the *MORC3*^–/–^ model and is plotted for 53 tandem repeats and 100 random genomic background sequences. *P* value was determined by the Wilcoxon rank-sum test. (**D**) Importance of each nucleotide for chromatin accessibility at MORC3-restricted tandem repeats predicted by the *MORC3*^–/–^ BPNet model. Spans of nucleotides with high attribution scores matching SPI, JUN or SP3 motifs are highlighted in red. Representative segments of three representative tandem repeats of 23 are shown. (**E**) Motifs from clusters in (**D**) that were predicted to drive chromatin accessibility at tandem repeats in the absence of MORC3 were inserted 5 or 35 times into random sequences. Accessibility was predicted using BPNet models that were trained on ATAC-seq data from *IFNAR1*^–/–^ *IFNAR2*^–/–^ and *IFNAR1*^–/–^ *IFNAR2*^–/–^ MORC3^–/–^BLaER1 monocytes. Annotations are from the Jaspar TF-motif database. (**F**) 1 to 35 SPI1, JUN, or SP3 motifs from (**D**) were inserted into random sequences, and chromatin accessibility was predicted using BPNet models that were trained on ATAC-seq data from *IFNAR1*^–/–^ *IFNAR2*^–/–^ and *IFNAR1*^–/–^ *IFNAR2*^*–/–*^ *MORC3*^*–/–*^ BLaER1 monocytes. (**G**) Indicated motif clusters in tandem repeats were mutated in silico, and MORC3 restriction (accessibility in *MORC3*^*–/–*^ - WT model) was predicted and is depicted normalized to the non-mutated WT sequence. 100% means that MORC3 still restricts this interval if the motif cluster is mutated. 0% means that MORC3 does not restrict this interval upon mutation of the motif cluster. Dot size reflects the number of motifs in the mutated cluster. (**H**) Expression of genes within 2 mb of a MORC3-restricted tandem repeat in BLaER1 monocytes comparing *IFNAR1*^–/–^ *IFNAR2*^–/–^, *IFNB1*^–/–^, Cas9 *STAT1*^–/–^ *STAT2*^–/–^ and Cas9 *STAT1*^–/–^ *STAT2*^–/–^ IFNB1-MRE^–/–^ monocytes, and matched *MORC3*^–/–^ monocytes from GSE183011 (*n* = 10). *P* values were calculated as part of DESeq2 using the Wald test and adjusted for multiple testing (*P*_adj_) using the Benjamini–Hochberg method. Interval_13710 is the TWIST2-MRE, Interval_22339 is the IFNB1-MRE. Source data are available online for this figure.

To test if motif clusters are linked to MORC3-mediated decreased accessibility at other tandem repeats, we employed BPNet, a deep learning sequence-to-function prediction framework (Avsec et al, 2021b). Unlike the Enformer model, which was pre-trained on ENCODE data from wild-type cells, the BPNet approach allowed training of separate models on ATAC-seq data from wild-type and *MORC3*^*–/–*^ monocytes to reveal the contribution of MORC3 to the predicted accessibility. BPNet models correctly predicted higher accessibility at the genomic MRE locus for the *MORC3*^*–/–*^ model compared to the wild-type model (Fig. EV7A). To assess whether these models captured accessibility restriction of motif clusters by MORC3, we performed an in silico marginalization analysis testing if SPI motif clusters are sufficient for MORC3-mediated accessibility restriction (similar to the experimental design in Fig. 5A). Specifically, we inserted the SPI motif from the MRE locus 1 to 35 times in background sequences and predicted chromatin accessibility using wild-type and *MORC3*^*–/–*^ models. Intriguingly, the BPNet models accurately learned that larger SPI motif clusters have reduced accessibility specifically in wild-type but not *MORC3*^*–/–*^ cells, while clusters with few motifs are predicted to be accessible by both models (Fig. EV7B). To determine whether transcription factor motif clusters are predicted to cause MORC3-mediated decreased accessibility at other loci, we analyzed additional MORC3-bound and -restricted tandem repeat regions (Fig. 1E) with BPNet. Most of the MORC3-restricted tandem repeats were correctly predicted to be more accessible by the *MORC3*^*–/–*^ model (Fig. EV7C). Attribution analyses using the *MORC3*^*–/–*^ BPNet model predicted clusters of ETS-, AP-1- and SP/KLF-like motifs, containing up to 61 motifs, as drivers of the accessibility (Fig. EV7D; Dataset EV2). Further marginalization analyses predict that MORC3 specifically limits accessibility when these motifs accumulated in a large (35×) compared to a small (5×) cluster (Fig. EV7E), predicting motif clusters to be sufficient for MORC3 to decrease accessibility. Consistently, the magnitude of MORC3-mediated accessibility reduction correlated with motif number (Fig. EV7F). Thus, BPNet predicts MORC3-mediated decrease of accessibility at tandem repeats to be driven by ETS-, AP-1- or SP/KLF-like transcription factor motif clusters (Fig. EV7G).

We next investigated if these tandem repeats are also repressed enhancers. However, only genes adjacent to one additional MORC3-bound and -accessibility restricted tandem repeat located near the *TWIST2* locus were activated upon MORC3 depletion (Interval 13,710 in Fig. EV7H). Notably, this MRE contained 26 SPI motifs, despite lacking overall sequence similarity to the IFNB1-regulating MRE (Interval 22,339 in Fig. EV7H). We refer to tandem repeats, whose enhancer activity is repressed by MORC3, as MORC3-repressed elements (MREs) and designate these two loci as the *IFNB1*-MRE and *TWIST2*-MRE, respectively. There were no general gene-regulatory activities of MORC3-restricted tandem repeats, suggesting that factors such as promoter compatibility, three-dimensional chromatin architecture, or transcription factor expression levels may modulate the enhancer potential. Collectively, these findings demonstrate that MORC3 restricts chromatin accessibility at tandem repeats harboring large transcription factor motif clusters and predict that motif clustering underlies MORC3-mediated accessibility limitation.

### PU.1 drives enhancer activity and MORC3-repression of the IFNB1-MRE

**Figure 6:**
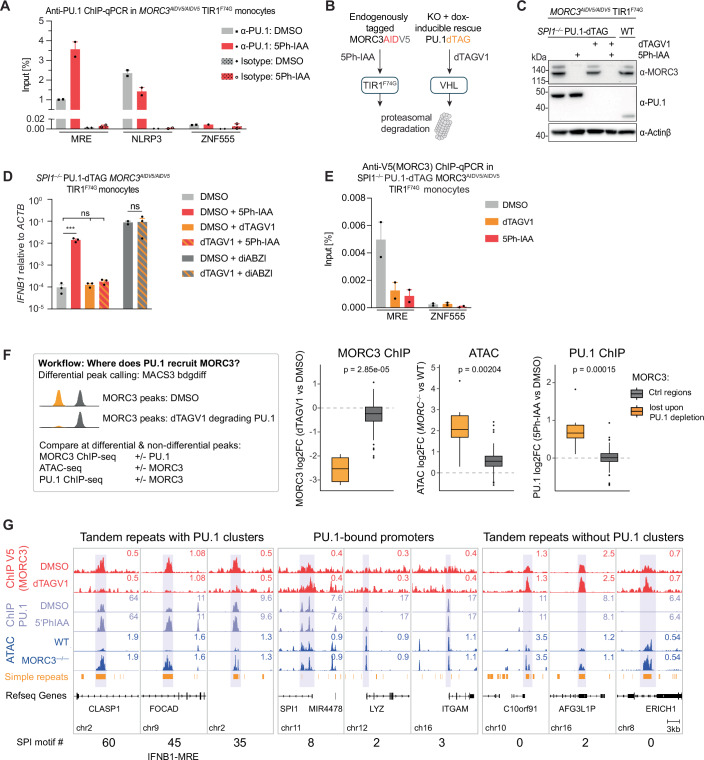
PU.1 activates *IFNB1* in the absence of MORC3. (**A**) ChIP-qPCR analysis of endogenous PU.1 in Cas9 *IFNAR1*^–/–^ *IFNAR2*^–/–^ *MORC3*^*AIDV5/AIDV5*^ TIR1^F74G^ BLaER1 monocytes that were treated as indicated for 24 h. The NLRP3 promoter, which contains two SPI motifs, serves as a positive control for PU.1 binding, the ZNF555 locus as a negative control. Missing datapoints equal non-detected values in qPCR. *n* = 2 independent experiments. (**B**) The dual degron approach. Endogenously tagged MORC3-AID-V5 is degraded upon 5Ph-IAA treatment in TIR1^F74G^ expressing cells, the dox-inducible PU.1-dTAG rescue construct in *SPI1*^–/–^ cells is degraded upon treatment with dTAGV1 via VHL-mediated ubiquitination. (**C**, **D**) Protein levels or *IFNB1* expression in Cas9 *IFNAR1*^–/–^ *IFNAR2*^–/–^ *MORC3*^*AIDV5/AIDV5*^ TIR1^F74G^ BLaER1 cells that are either *SPI1*^*WT/WT*^ or *SPI1*^–/–^ + dox-inducible PU.1-dTAG cDNA-rescue. If indicated, cells were pretreated with dTAGV1 for 1 h before stimulation with 5Ph-IAA or DMSO for 24 h. The STING agonist diABZI was applied for the last 3 h of the experiment. Data is shown as one representative of two immunoblots or represents the mean + SEM from *n* = 2 or 3 independent experiments. ***P < 0.0001; ns, *P* > 0.9999 as determined by one-way ANOVA and Bonferroni’s post hoc test. (**E**) ChIP-qPCR analysis of MORC3-V5 in dox-stimulated Cas9 *IFNAR1*^–/–^ *IFNAR2*^–/–^ *MORC3*^*AIDV5/AIDV5*^ TIR1^F74G^ *SPI1*^–/–^ PU.1-dTAG BLaER1 monocytes that were treated as indicated for 24 h. *n* = 2 independent experiments. (**F**) 6 MORC3-V5 peaks with reduced MORC3 signal upon PU.1 degradation were identified by bdgdiff (see “Methods”; orange). Differential signal for MORC3-V5 binding, accessibility, and PU.1 binding compared to 340 (MORC3), 336 (ATAC), or 334 (PU.1) control MORC3-V5 peaks (gray). The center line indicates the median (50th percentile). The box bounds mark the first (Q1, 25th percentile) and third quartiles (Q3, 75th percentile). The whiskers extend to the most extreme data points within 1.5 times the interquartile range (IQR  =  Q3–Q1) from the quartiles. *P* values were determined by the Wilcoxon rank-sum test. (**G**) Genome browser view of representative loci from (**F**) and control regions. Source data are available online for this figure.

**Figure EV8:**
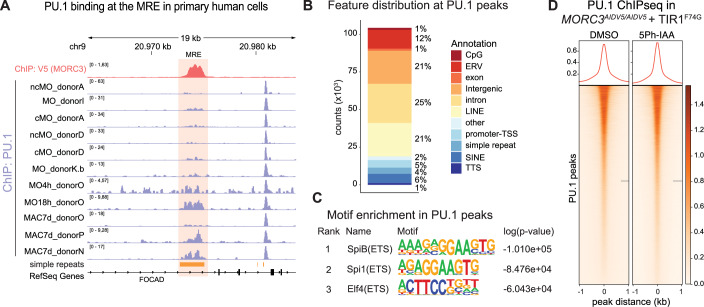
PU.1 binds to the IFNB1-MRE in primary human myeloid cells. (**A**) Genome browser view of PU.1 ChIP-seq coverage in primary human myeloid cells and MORC3-V5 ChIP-seq in BLaER1 monocytes at the human IFNB1-MRE. PU.1 data is from GSE128837. (**B**, **C**) Genomic feature annotation and top 3 enriched motifs across PU.1 ChIP-Seq peaks in Cas9 *IFNAR1*^–/–^ *IFNAR2*^–/–^ *MORC3*^*AIDV5/AIDV5*^ TIR1^F74G^ BLaER1 monocytes. (**D**) Read-density heatmap showing the normalized coverage of PU.1 ChIP-seq signal centered around PU.1 peaks from *IFNAR1*^–/–^ *IFNAR2*^–/–^ *MORC3*^*AIDV5/AIDV5*^ TIR1^F74G^ BLaER1 monocytes treated with DMSO or 5Ph-IAA for 24 h. Source data are available online for this figure.

Next, we set out to identify the transcription factor that mediates IFNB1-MRE activities, with the initial focus on the enhancer activity. We hypothesized that PU.1 (encoded by *SPI1*) may be involved, because PU.1 is the SPI transcription factor family member with the highest expression in monocytes. Monocyte-specific high PU.1 expression could also explain why the MRE-IFNB1 pathway is specifically active in this cell type (Gaidt et al, 2021b) (Fig. 4A). PU.1 bound the IFNB1-MRE in BLaER1 monocytes at steady state, as measured by ChIP–qPCR (Fig. 6A) and in public ChIP–seq datasets from primary human myeloid cells (Fig. EV8A, Data ref:(Minderjahn et al, 2019). Notably, MORC3 deletion increased PU.1 binding at the IFNB1-MRE, but not at the *NLRP3* promoter, which contains two SPI motifs (Fig. 6A). This suggests that MORC3 specifically antagonizes PU.1 binding at the IFNB1-MRE motif cluster. To determine whether MORC3 globally limits PU.1 occupancy, we performed anti-PU.1 ChIP–seq in the presence or absence of MORC3 in BLaER1 monocytes. PU.1 bound broadly to the genome at sites with canonical PU.1 motifs (Fig. EV8B,C). Deletion of MORC3 caused no global change in PU.1 binding (Fig. EV8D), suggesting that MORC3 specifically constrains PU.1 binding at the IFNB1-MRE, rather than broadly limiting PU.1 occupancy across the genome.

**Figure EV9:**
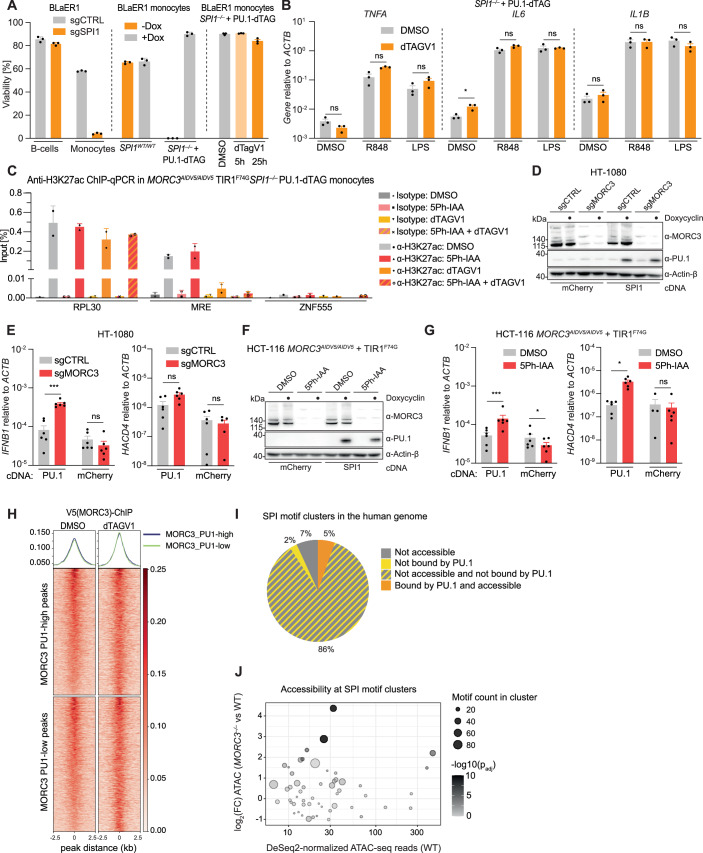
Characterization of PU.1-dTAG BLaER1 monocytes and PU.1 levels are the limiting factor for IFNB1-MRE activation in other cells. (**A**) Cell viability of Cas9 *IFNAR1*^–/–^ *IFNAR2*^–/–^ *MORC3*^*AIDV5/AIDV5*^ TIR1^F74G^ BLaER1 B-cells or monocytes expressing sgCTRL or sgSPI1 (left); Cas9 *IFNAR1*^–/–^ *IFNAR2*^–/–^ *MORC3*^*AIDV5/AIDV5*^ TIR1^F74G^ *SPI1*^*WT/WT*^ or Cas9 *IFNAR1*^–/–^ *IFNAR2*^–/–^ *MORC3*^*AIDV5/AIDV5*^ TIR1^F74G^ *SPI1*^–/–^ PU.1-dTAG-cDNA BLaER1 monocytes stimulated with doxycycline (middle). The PU.1-dTAG rescue construct is under the control of a dox-inducible promoter. And cell viability of dox-stimulated Cas9 *IFNAR1*^–/–^ *IFNAR2*^–/–^ *MORC3*^*AIDV5/AIDV5*^ TIR1^F74G^ *SPI1*^–/–^ PU.1-dTAG-cDNA BLaER1 monocytes stimulated with dTAGV1 for indicated time (right). Data is shown as mean + SEM of *n* = 3 independent experiments. (**B**) Gene expression analysis of dox-stimulated Cas9 *IFNAR1*^–/–^ *IFNAR2*^–/–^ *MORC3*^*AIDV5/AIDV5*^ TIR1^F74G^ *SPI1*^–/–^ PU.1-dTAG-cDNA BLaER1 monocytes stimulated with dTAGV1 for 24 h and with the indicated TLR-ligands for 4 h. Data are shown as mean + SEM of *n* = 3 independent experiments. *TNFA*: DMSO, *P* = 0.3794; R848, *P* = 0.0767; LPS, *P* = 0.2088; *IL6*: DMSO, *P* = 0.0224; R848, *P* = 0.4854; LPS, *P* > 0.9999; *IL1B*: DMSO, *P* > 0.9999; R848, *P* > 0.9999; LPS, *P* = 0.9586 as determined by two-way ANOVA and Bonferroni’s post hoc test. *P < 0.05; ns, not significantly different. (**C**) ChIP-qPCR analysis of H3K27ac in dox-stimulated Cas9 *IFNAR1*^–/–^ *IFNAR2*^–/–^ *MORC3*^*AIDV5/AIDV5*^ TIR1^F74G^ *SPI1*^–/–^ PU.1-dTAG BLaER1 monocytes from *n* = 2 independent experiments. If indicated, cells were pretreated with dTAGV1 for 1 h before stimulation with 5Ph-IAA or DMSO for 24 h. The RPL30 promoter serves as a positive control for H3K27ac, the ZNF555 locus as a negative control. Missing datapoints equal non-detected values in qPCR. (**D**–**G**) Cas9 HT-1080 fibrosarcoma and *MORC3*^*AIDV5/AIDV5*^ TIR1^F74G^ HCT-116 colorectal carcinoma cell lines were transduced with dox-inducible cDNAs encoding PU.1 or mCherry, and MORC3 was deleted with lentivirus expressing anti-MORC3 sgRNAs or degraded with 5Ph-IAA. Gene expression of IFNB1-MRE-regulated genes *IFNB1* and *HACD4* from *n* = 6 independent experiments or one representative immunoblot of two upon treatment with doxycycline for 48 h. HT-1080: *IFNB1*: PU.1, *P* = 0.0001; mCherry, *P* = 0.3647. *HACD4*: PU.1, *P* = 0.3217; mCherry, *P* > 0.9999. HCT-116: *IFNB1*: PU.1, *P* = 0.0002; mCherry, *P* = 0.0414. *HACD4*: PU.1, *P* = 0.0105; mCherry, *P* > 0.9999 as determined by two-way ANOVA and Bonferroni’s post hoc test. ***P < 0.001; *P < 0.05; ns, not significantly different. Missing datapoints equal non-detected values in qPCR. (**H**) Read-density heatmap showing the normalized coverage of anti-V5 (MORC3) ChIP-seq and centered around MORC3-V5 peaks in dox-stimulated Cas9 *IFNAR1*^–/–^ *IFNAR2*^–/–^ *MORC3*^*AIDV5/AIDV5*^ TIR1^F74G^ *SPI1*^–/–^ PU.1-dTAG BLaER1 monocytes treated with DMSO or dTAGV1 for 24 h. MORC3-V5 peaks were grouped by high and low PU.1 signal. (**I**) 992 SPI motif clusters with >15 binding sites in the human genome were grouped according to chromatin accessibility (ATAC-seq) and PU.1 ChIP-seq signal. (**J**) Chromatin accessibility determined by ATAC-seq at accessible and PU.1-bound SPI-motif clusters comparing *IFNAR1*^–/–^ *IFNAR2*^–/–^ and *IFNAR1*^–/–^ *IFNAR2*^–/–^ *MORC3*^–/–^ BLaER1 monocytes (*n* = 3 independent experiments). Data is from GSE183011. *P* values were calculated as part of DESeq2 using the Wald test and adjusted for multiple testing (*P*_adj_) using the Benjamini–Hochberg method. Source data are available online for this figure.

To investigate the function of PU.1 in the MRE-IFNB1 pathway, we employed both loss- and gain-of-function approaches. The essential role of PU.1 in monocytes/macrophages (McKercher et al, 1996), including BLaER1 cells (Fig. EV9A), made constitutive genetic loss-of-function experiments not feasible. We thus turned to a second degron system called dTAG (Nabet et al, 2018) to study the involvement of the essential protein PU.1 by rapidly inducing its loss before loss of cell viability (Fig. 6B). Knockout-rescue experiments using a dox-inducible PU.1-dTAG cDNA in *MORC3*^*AIDV5/AIDV5*^ TIR1^F74G^ *SPI1*^*–/–*^ BlaER1 monocytes showed that the PU.1-dTAG construct rescued cell viability (Fig. EV9A) and enabled rapid PU.1 degradation independently and in parallel to AID-mediated MORC3 degradation (Fig. 6C). Strikingly, PU.1 degradation completely abolished IFN induction upon MORC3 depletion (Fig. 6D). Cell viability decreased only slightly following PU.1 degradation (Fig. EV9A), and PU.1 depletion did not affect IFN induction when the STING agonist diABZI was added during the final three hours of the experiment (Fig. 6D). Similarly, responses to TLR agonists were not impaired upon PU.1 degradation (Fig. EV9B). Therefore, PU.1 is specifically required for MRE-mediated *IFNB1* induction in the absence of MORC3. Consistent with steady state binding of PU.1 to the IFNB1-MRE, PU.1 was necessary for establishing the histone mark H3K27ac, commonly associated with active enhancers, at the IFNB1-MRE in the presence and absence of MORC3 but not at the RPL30 promoter (Fig. EV9C). H3K27ac levels did not further increase upon MORC3 loss, indicating that the IFNB1-MRE-enhancer is pre-activated by PU.1 but repressed by MORC3. As a gain-of-function approach, we overexpressed PU.1 in PU.1-negative HCT-116 colorectal carcinoma cells and the PU.1^low^ fibrosarcoma HT-1080 cell line. In both cases, PU.1 overexpression activated *IFNB1* and the IFNB1-MRE-regulated gene *HACD4* specifically upon loss of MORC3 (Fig. EV9D–G). Thus, PU.1 is sufficient to drive IFNB1-MRE-regulated gene expression when MORC3-mediated repression is relieved.

Since the PU.1 motif cluster mediates both enhancer repression and activation (Figs. 5A and EV6A), we investigated the role of PU.1 in MORC3-mediated repression. Because MORC3 is required for DAXX binding and H3.3 incorporation (Fig. 3), we focused on MORC3 binding as the proximal event of this repression cascade. ChIP-qPCR revealed that MORC3 recruitment to the IFNB1-MRE depended on PU.1 (Fig. 6E). Genome-wide ChIP–seq analysis demonstrated that PU.1 does not generally recruit MORC3, as MORC3 binding was not globally reduced following PU.1 depletion (Fig. EV9H). Despite this global pattern, we identified 6 MORC3 peaks that were significantly reduced upon PU.1 degradation (Fig. 6F). MORC3 reduced accessibility and limited PU.1 binding at these loci that were all tandem repeats with PU.1 motif clusters. In contrast, PU.1 did not recruit MORC3 to motif clusters of other transcription factors (e.g., JUN) in MORC3-restricted tandem repeats or PU.1-bound sites containing fewer PU.1 motifs (Fig. 6G). These findings suggest that PU.1 establishes a self-limiting negative feedback loop at tandem repeats containing dense PU.1 motif clusters: PU.1 recruits MORC3, which recruits DAXX to limit accessibility and PU.1 binding. To understand why PU.1-dependent MORC3 recruitment sites are rare, we computationally identified all sequence-based SPI (PU.1) motif clusters containing more than 15 motifs in the human reference genome. Of these ~1000 clusters, only ~50 were accessible and bound by PU.1 in the absence of MORC3 (Fig. EV9I), indicating that additional heterochromatin pathways restrict accessibility at most sites. Among the accessible, PU.1-bound clusters, several ones with high motif numbers showed a trend toward increased chromatin accessibility upon MORC3 loss (Fig. EV9J). In summary, PU.1 is a bifunctional transcription factor that mediates both activation and repression of the IFNB1-MRE. In wild-type cells, PU.1 specifically recruits MORC3, driving repression, and upon deletion of MORC3, it mediates enhancer function to drive gene expression.

**Figure 7:**
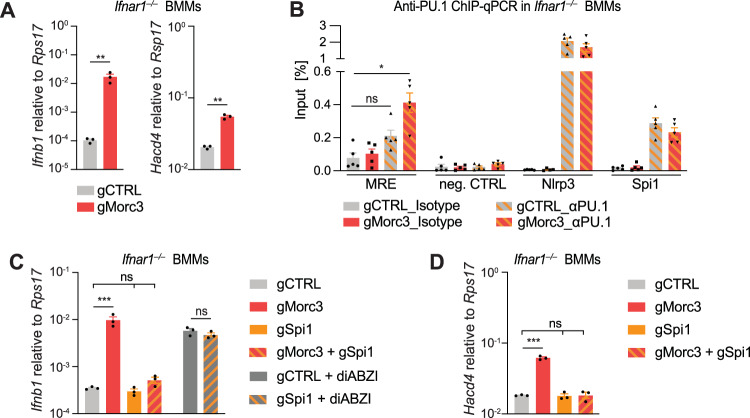
MORC3 represses PU.1 activation of the MRE-IFNB1 axis in primary mouse macrophages. (**A**) *Ifnar1*^*–/–*^ bone-marrow macrophages (BMMs) were nucleofected with Cas9:gRNA-RNPs targeting Morc3 or a control region. Gene expression of IFNB1-MRE-regulated genes at day 2 after nucleofection. *Ifnb1*, *P* = 0.0046; *Hacd4*, *P* = 0.0052 as determined by paired, two-sided *t* test. (**B**) Anti-PU.1 ChIP-qPCR in *Ifnar1*^*–/–*^ BMMs nucleofected with indicated Cas9-RNPs for 5 days. The *Nlrp3* and *Spi1* promoters serve as positive controls for PU.1 binding. Neg. CTRL is a region adjacent to the *Ifnb1*-MRE. Data represent the mean + SEM from *n* = 5 independent experiments. *P = 0.0212; ns, *P* > 0.9999 as determined by two-way ANOVA and Bonferroni’s post hoc test. (**C**, **D**) Gene expression analysis in *Ifnar1*^–/–^ BMMs two days after nucleofection with Cas9:RNPs. When indicated, cells were treated with diABZI during the last 4 h of the experiment. ***P < 0.001; ns, *P* > 0.9999 unless *Ifnb1*: gCTRL vs gSPI1 + gMORC3, *P* = 0.7698 as determined by ordinary one-way ANOVA and Bonferroni’s post hoc test. Unless otherwise indicated data represents the mean + SEM of *n* = 3 independent experiments. Source data are available online for this figure.

**Figure EV10:**
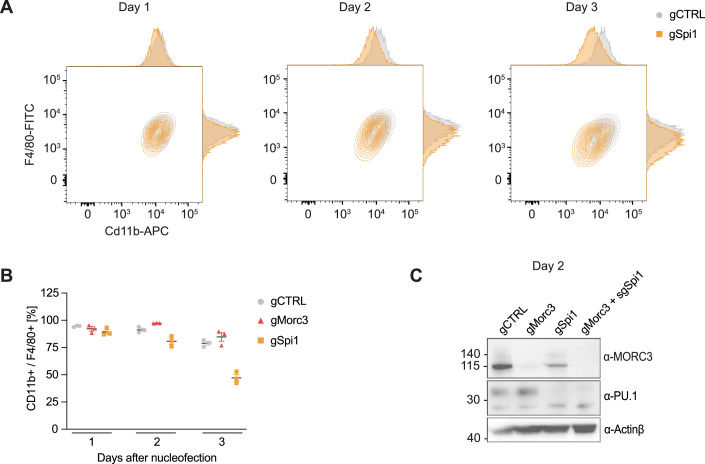
Genetic deletion of the essential factor PU.1 in BMMs. (**A**, **B**) Cd11b and F4/80 FACS-analysis of *Ifnar1*^*–/–*^ BMMs at day 1, 2, or 3 after nucleofection with indicated Cas9:RNPs depicted as one representative experiment (**A**) and mean ± SEM of *n* = 3 independent experiments. (**C**) Immunoblot analysis of *Ifnar1*^*–/–*^ BMMs at day 2 after nucleofection with indicated Cas9:RNPs showing one representative immunoblot of two. Source data are available online for this figure.

To validate this model in primary cells, we turned to bone-marrow-derived macrophages (BMMs). We used *Ifnar1*^−^^/^^−^ mice to prevent secondary IFN effects upon loss of MORC3. The mouse Ifnb1-MRE is a tandem repeat consisting of nine 100 bp repeats harboring 15 PU.1 motifs (Fig. 5C). Cas9:RNP nucleofection-mediated deletion of *Morc3* induced expression of *Ifnb1* and another IFNB1-MRE proximal gene, *Hacd4* (Fig. 7A). PU.1 binding could be robustly detected at the mouse IFNB1-MRE upon *Morc3* deletion but not at steady state (Fig. 7B). *Morc3* deletion had no effect on PU.1 binding at other sites confirming that MORC3 specifically antagonizes PU.1 binding at the IFNB1-MRE. As PU.1 is essential in BMMs, genetic deletion caused loss of myeloid identity around day 2 to 3 after nucleofection, as indicated by reduced CD11b and F4/80 expression (Fig. EV10A,B). However, PU.1 protein was already efficiently depleted by day two after nucleofection (Fig. EV10C), providing a short window for loss-of-function analysis. Within this window, PU.1 was required for *Ifnb1* and *Hacd4* induction upon *Morc3* deletion, but not for *IFNB1* induction by the STING-agonist diABZI (Fig. 7C,D). Thus, in primary macrophages, MORC3 antagonizes PU.1 binding at the IFNB1-MRE to inhibit enhancer activation. In absence of MORC3 PU.1 activates *Ifnb1*.

## Discussion

Here, we show that MORC3 restricts chromatin accessibility at tandem repeat elements that harbor exceptionally dense clusters of homotypic transcription factor binding motifs. The IFNB1-MRE is a tandem repeat containing 45 PU.1 binding sites, and PU.1 mediates IRF3/7-independent *IFNB1* induction through this element. Under steady-state conditions, MORC3 represses MRE activity by recruiting DAXX and enabling H3.3 incorporation, thereby maintaining the element in a compacted chromatin state. Notably, PU.1 motif clusters mediate both MORC3 recruitment—leading to repression at steady state—and exceptionally strong, synergistic enhancer activity leading to *IFNB1* induction upon MORC3 loss. Together, our findings identify homotypic motif clusters as the basis of a repressed enhancer that functions as an immune “insurance mechanism,” enabling robust interferon induction upon perturbation of MORC3. While this manuscript was under review, another study also identified the IFNB1-MRE as a repressed enhancer (Goffeney et al, 2025). Our work extends this notion by identifying PU.1 as the required driver of enhancer activity, elucidating the genetic mechanism underlying IFNB1-MRE repression, and defining the specificity of MORC3 for homotypic transcription factor motif clusters. These mechanistic insights explain the specificity of MORC3-mediated IFN repression in two ways. First, MORC3-dependent repression requires unusually large clusters of homotypic PU.1 binding sites. Such high-density motif clusters are exceedingly rare in the genome and, in most cases, are already inaccessible due to MORC3-independent heterochromatinization. Accordingly, we identified only a small number of sites at which PU.1 can recruit MORC3 and is itself inhibited. In contrast, typical gene-regulatory elements harbor fewer motifs and integrate signals from multiple distinct TFs. Even super-enhancers, clusters of regulatory elements characterized by numerous TF binding sites, typically integrate motifs from diverse TFs and lack intrinsic repressive functionality (Hnisz et al, 2013; Whyte et al, 2013). Thus, the identified bifunctional homotypic motif clusters within tandem repeats constitute a rare class of regulatory elements distinct from super-enhancers. Second, not all TFs are repressed by MORC3. We speculate that this selectivity reflects differences in the ability of transcription factors to recruit MORC3. MORC3 could be recruited through direct interactions with specific transcription factors, as suggested for PU.1 (Goffeney et al, 2025), or via a common recruitment mechanism that operates at motif clusters. We speculate that the ability of TFs to facilitate MORC3 repression at motif clusters may relate to their ability to engage the SUMOylation pathway, as SUMOylation is known to mediate transcriptional repression and is observed among several TFs identified in the MORC3-restricted clusters (Sapetschnig et al, 2002; Tempe et al, 2014). SUMO-mediated repression of these TFs specifically targets synergistic activation facilitated by the presence of multiple homotypic binding motifs (Holmstrom et al, 2003; Holmstrom et al, 2008). Interestingly, SUMOylation is required for IFNB1-MRE repression (Goffeney et al, 2025). However, the underlying mechanism remains unclear, and it is speculative if the role of SUMO in MORC3 repression extends beyond the reported SUMO-dependent MORC3–DAXX interaction (Groh et al, 2021).

While TF cluster-mediated repression by MORC3 explains its remarkable specificity toward tandem repeat enhancers, the mechanisms underlying MORC3 repression of other targets, such as endogenous retroviruses (ERVs) and viral DNA genomes, remain unclear. Given the rarity of dense motif clusters, it is unlikely that TF cluster–based repression also operates at these sites. Instead, viral targets may engage distinct mechanisms of MORC3 recruitment or activation.

Beyond explaining the specificity of the repression, the dense cluster of PU.1 motifs also explains the exceptional strength and speed of IFNB1 induction upon loss of MORC3. This represents a novel mechanism for inflammatory signal amplification, distinct from classical pathways. Conventional pattern recognition receptors (PRRs) achieve rapid pro-inflammatory transcription through signal transduction cascades involving second messengers, enzymatic amplification, supramolecular organizing centers (SMOCs), and stimulus-dependent TF activation. In contrast, MORC3 acts downstream at the transcriptional repression level, independently of these canonical amplification pathways. Consequently, immune activation upon MORC3 inhibition requires an alternative mechanism for swift and robust inflammatory induction, provided here by the exceptionally high density of TF motifs within these clusters.

How are these high-density motif clusters generated? We propose that the tandem repeat architecture of repressed enhancers facilitates the evolutionary accumulation of TF motifs. Tandem repeats are inherently unstable genomic elements, frequently expanding or contracting through replication slippage, thus providing an efficient mechanism for accumulating multiple motif copies. This represents a unique amplification mechanism specific to tandem repeats, expanding their roles in gene regulation. The following observations support the idea of repeat-mediated expansion of TF motif clusters: (1) variability in repeat numbers within the human IFNB1-MRE, classifying it as a variable number tandem repeat (VNTR); (2) limited sequence conservation among different human MREs; and (3) consistent occurrence of high-density SPI motifs within IFN-associated MREs across diverse mammalian species. Future studies should investigate whether polymorphisms in the IFNB1-MRE VNTR region correlate with disease susceptibility, such as increased vulnerability to viral infections or auto-inflammatory conditions.

Notably, TF motif cluster-mediated repression by MORC3 creates gene-regulatory switches, whose broader relevance may extend beyond host-pathogen interactions, where MORC3 degradation triggers immediate and robust IFN induction. It is conceivable that viral inhibition of MORC3 represents only one mechanism triggering the rapid switch from repression to potent enhancer activity at TF motif clusters. Alternative mechanisms, such as modulation of a TF’s ability to recruit MORC3 or post-translational modifications of MORC3 itself, could similarly disrupt repression, facilitating rapid cellular decision-making. Future studies should therefore explore MORC3-mediated repression in other biological contexts, such as developmental pathways, where synergistic TF activation at motif clusters may similarly regulate swift cellular responses. Interestingly, PU.1 also binds cryptic enhancers repressed by SETDB1 in hematopoietic stem cells (Kazerani et al, 2024). Although it remains unclear whether these enhancers are similarly regulated by MORC3, enhancer repression may represent a common regulatory strategy shared between immune sensing and developmental processes, enabling rapid biological decision-making.

## Methods

**Table:** Reagents and tools table

Reagent/resource	Reference or source	Identifier or catalog number
**Experimental models**		
BLaER1	Gift from T. Graf (CRG Barcelona) and Veit Hornung (LMU Munich)	RRID:CVCL_VQ57
HCT-116	UC Berkeley Cell Culture Facility	RRID:CVCL_0291
HT-1080 iCas9	Gift from J. Zuber (IMP Vienna)	RRID:CVCL_0317
Lenti-X 293 T	Gift from J. Zuber (IMP Vienna)	RRID:CVCL_4401
Ifnar1^−/−^ mice: B6(Cg)-Ifnar1tm1.2Ees/J (M. musculus)	Gift from A. Bergthaler (Medical University of Vienna)	JAX#028288
S2 cells (D. melanogaster)	Gift from A. Stark (IMP Vienna)	Thermofisher R69007
**Recombinant DNA**		
MRE/GFP reporter plasmids	This study; based on: Addgene #60523	Dataset EV3
pCMV(CAT)T7-SB100	Addgene	#34879
pVSV-G	Gift from Veit Hornung (LMU Munich)	
pd8.9	Gift from Veit Hornung (LMU Munich)	
hU6-sgRNA1-mU6-sgRNA2-EF1αs-FP-Res	Gift from J. Zuber (IMP Vienna)	
*Endogenous tagging homology donor 1:* pHDR_miniAID-3xV5-P2A-HygroR-MORC3-Homology-Arms	This study; based on pEF-BOS: NovoPro Biosciences (V007643)	
*Endogenous tagging homology donor 2:* pHDR_miniAID-3xV5-P2A-NeoR -MORC3-Homology-Arms	This study; based on pEF-BOS: NovoPro Biosciences (V007643)	
U6-sgRNA-CMV-mCherry-T2A-Cas9	Gift from Veit Hornung (LMU Munich); PMID: 25186908	
pRRL-TIR1F74G-P2A-hCD2	Gift from J. Zuber (IMP Vienna)	
lentiCas9-Blast	Addgene	#52962
pLIP_SPI1	This study. Based on pLIP: PMID: 34759314	
pLIP_mCherry	This study. Based on pLIP: PMID: 34759314	
**Antibodies**		
**ChIP/Cut&Run**		
Anti-PU.1	Cell Signaling Technologies	Clone: 9G7; Cat:2258S
Anti-H3K27ac	Abcam	Clone: polyclonal; Cat:ab4729
Anti-V5	Thermo Fisher	Clone: SV5-Pk1; Cat:781999
Anti-H3K4me3	Cell Signaling Technologies	Clone: C42D8; Cat:9751
Isotype control	Cell Signaling Technologies	Cat:2729
Drosophila spike-in antibody	Active motif	Cat:61686
Anti-H3K9me3	Cell Signaling Technologies	Clone: D4W1U; Cat:1369
Anti-total H3	Abcam	Polyclonal; Cat:AB1791
Anti-H3.3	Abcam	Clone: EPR17899; Cat:AB176840
**Immunoblotting**		
Anti-MORC3	Proteintech	Polyclonal; Cat:24994-1-AP
Anti-PU.1	Cell Signaling Technologies	Clone: 9G7; Cat:2258S
Anti-DAXX	Cell Signaling Technologies	Clone: 25C12; Cat:4533
Anti-beta-Actin-HRP	Sigma-Aldrich	Clone: AC-15; Cat:A3854
Anti-rabbit-IgG-HRP	Cell Signaling Technologies	Clone: Cat:7074P2
**Flow cytometry**		
Mouse anti-human CD11b-BV711	Biolegend	Clone: ICRF44; Cat:301344
Mouse anti-human CD19-APC	Biolegend	Clone: HIB19; Cat:302212
Rat anti-mouse F4/80-FITC	Biolegend	Clone: BM8; Cat:123107
Rat anti-mouse CD11b-APC	Biolegend	Clone: M1/70; Cat:101211
**Oligonucleotides and other sequence-based reagents**		
qPCR Primers	This study	Dataset EV3
Genotyping Primers	This study	Dataset EV3
sgRNA target sites	This study	Dataset EV3
**Chemicals, enzymes, and other reagents**		
DMEM	Thermo Fisher	41965039
RPMI 1640	Gibco	41965039
FCS	Gibco	10500-064; Lot: 2440058H
L-glutamine	Gibco	25030
Sodium Pyruvate	Sigma-Aldrich	S8636
Penicillin/Streptomycin	Sigma-Aldrich	P0781
Human recombinant IL-3	PeproTech	200-03
Human recombinant M-CSF	PeproTech	300-25
Beta-Estradiol	Sigma-Aldrich	E8875
Schneider’s S2 medium	Gibco	21720001
ACK Lysis Buffer	Gibco	A10492-01
L-929 conditioned medium	Gift from P. Kovarik (University of Vienna)	
Alt-R^TM^ tracer RNA	IDT	1072533
Alt-R^TM^ CRISPR RNA	IDT	Dataset EV3
Alt-R^TM^ Sp. Cas9 Nuclease V3	IDT	1081059
Electroporation enhancer	IDT	1075915
5Ph-IAA	Bio-Academia	30-003
dTAGV1	Tocris Bioscience	6914
Doxycycline	Sigma-Aldrich	Sigma-Aldrich
LPS	Invivogen	tlrl-3pelps
R848	Invivogen	tlrl-r848-1
diABZI	Invivogen	tlrl-diabzi
DNase I	New England Biolabs	M0303S
DNA restriction enzymes	Thermo Scientific; FastDigest	
Urea	Sigma-Aldrich	U4884
TRIS	Sigma-Aldrich	T6066
NaCl	Merck	1.06406.5000
EDTA	AppliChem	A1103,1000
EGTA	Carl Roth	3054.2
Na-dodecylsulfate	Thermo Scientific	24730020
DTT	Varl Roth	6908.3
KCl	Sigma-Aldrich	60130
MgCl_2_	Sigma-Aldrich	63065
LiCl	Merck	1.05679.0250
(NH4)2SO4	Sigma-Aldrich	A4418
MnCl_2_	Thermo Scientific	J63150.AD
CaCl_2_	Sigma-Aldrich	31307
NaHCO_3_	Merck	27778.293
Triton-X100	Sigma-Aldrich	RES3103T-A101X
Proteinase K	New England Biolabs	P8107
T4 DNA ligase	Thermo Scientific	EL0011
Puromycin	Invivogen	ant-pr-1
Geneticin (G418)	Gibco	11811-031
Blasticidin S-hydrochloride	Invivogen	Ant-bl-05
Hygromycin B Gold	Invivogen	ant-hg-1
Lipofectamine2000	Invitrogen	1000144469
IGEPAL	Sigma-Aldrich	56741
Complete-Mini EDTA-free Proetase Inhibitor Tablets	Roche	11836170001
Laemmli SDS sample buffer	Thermo Scientific	J61337
Tween-20	Thermo Scientific	515333
Para-formaldehyde	Thermo Scientific	28908
Hepes	Sigma-Aldrich	H4034
Glycerol	AppliChem	A0970
Na-deoxycholate	Sigma-Aldrich	D6750
N-lauroylsarcosine	Thermo Scientific	J60040.18
Spermidine	Sigma-Aldrich	S0266-5G
ConcavalinA-Biotin	Sigma-Aldrich	C2272-10MG
Digitonin	Merck	300410
ProteinA/G MNase	VBC-MBS core facility	
RNase A	Sigma-Aldrich	11119915001
Glycogen	Roche	109011393001
**Software**		
GraphPad Prism v10.2.1	www.graphpad.com	
ImageLab v6.0.1	www.bio-rad.com	
FlowJo v10.10.0	https://www.flowjo.com/	
nf-core/cutandrun pipeline	v3.2.2	10.5281/zenodo.5653535
nf-core/chipseq pipeline	v2.1.0	10.5281/zenodo.3240506
Bowtie2	v2.5.1	PMID: 22388286
Samtools	v1.17	PMID: 33590861
deepTools suite	v3.5.6	PMID: 24799436
HOMER	v6.0	PMID: 20513432
bedtools	2.30.0	PMID: 20110278
MACS3	3.0.1	PMID: 18798982
DESeq2	v1.42.1	PMID: 25516281
nf-core ATAC-seq pipeline	v2.1.2	10.5281/zenodo.2634132
RepEnrich2		PMID: 25012247
Danbing-tk		PMID: 34253730; PMID: 37037626
BWA	0.7.17	PMID: 19451168
featureCounts	v2.0.2	PMID: 24227677
Snakemake	v9.5.1	PMID: 34035898
Enformer		PMID: 34608324
bpnet-lite	v0.8.0	https://github.com/jmschrei/bpnet-lite
tangermeme	v0.4.4	https://github.com/jmschrei/tangermeme
TOMTOM (MEME Suite)	v5.5.9	PMID: 17324271
FIMO (MEME Suite)	v5.5.9	PMID: 21330290
Tandem Repeats Finder		PMID: 9862982
Clustal Omega		PMID: 21988835
Jalview	v2.11.4.1	PMID: 19151095
**Other**		
LSR Fortessa Flow Cytometer	BD	
NovaSeqX	Illumina	
NextSeq2000	Illumina	
4D nucleofector	Lonza	
Gene Pulser Xcell	BioRad	
CFX Touch Real-Time PCR Detection System	BioRad	
ChemiDoc Go	BioRad	
BioRuptor	Diagenode	
RNA-isolation kit	VBC-MTD core facility	
Oligo-dT Beads	VBC-MTD core facility	
Taq-qPCR-MasterMix	VBC-MTD core facility	
Phusion High Fidelity PCR Polymerase	Thermo Fisher	F-530L
NuPAGE^TM^ 4-12% Bis-Tris Gel	Invitrogen	NP0335BOX
Immobilon-FL PVDF membrane	Thermo Fisher	88518
Bradford Assay Kit	Thermo Fisher	23200
ECLTM Prime Reagent Kit	Cytiva	RPN2232
Mouse FC Block	BD	553142
ProteinA-coated magnetic beads	Cytiva	GE28-9670-62
Bovine Serum Albumin (BSA)	Thermo Fisher	BP1600-100
ChIP clean & concentrator kit	Zymo Research	D5205
NEB Next Ultra II Library Prep Kit	New England Biolabs	E7645L
NEB Next Multiplex Oligos	New England Biolabs	E7600
MinElute Kit	Qiagen	28004
Dynabeads^TM^ MyOneTM Streptavidin T1	Thermo Fisher	65601
E.coli DH5alpha	VBC-MBS core facility	

### Cell culture

BLaER1 cells were cultured in RPMI 1640 medium supplemented with L-glutamine, sodium pyruvate, penicillin–streptomycin (Thermo Fisher), and 10% (v/v) FCS (Gibco). Lenti-X 293T, HCT-116, and HT-1080 cells were cultivated in DMEM High Glucose medium (Thermo Fisher) containing the same supplements. A total of 700,000 BLaER1 cells per well of a 12-well plate or 70,000 BLaER1 cells per well of a 96-well plate were trans-differentiated into monocytes for 5–6 days in medium containing 10 ng ml^−1^ human recombinant (hr) IL-3, 10 ng ml^−1^ h-CSF-1 (M-CSF) (both PeproTech), and 100 nM β-estradiol (Sigma-Aldrich). Drosophila melanogaster S2 cells were cultured in Schneider’s S2 cell medium supplemented with 5% (v/v) FCS and penicillin–streptomycin (Thermo Fisher). BLaER1 cells were a gift from T. Graf (CRG, Barcelona, Spain) and V. Hornung (LMU Munich, Germany). HCT-116 cells were from the UC Berkeley Cell Culture Facility. HT-1080 inducible Cas9 (iCas9) cells and Lenti-X 293 T cells were a gift from J. Zuber (IMP Vienna). S2 cells were a gift from A. Stark (IMP Vienna). All human cells were cultured at 37 °C and 5% CO_2_, and S2 cells were cultured at 30 °C and 5% CO_2._ All cells were regularly tested negative for mycoplasma contamination, and cell line identity was validated by STR genotyping at the IMP-MBS Core Facility.

### Isolation and nucleofection of bone-marrow-derived macrophages

All animals were maintained in the specific pathogen-free animal facility of the Research Institute of Molecular Pathology, and all procedures were carried out according to an ethical animal license that is approved and regularly controlled by the Austrian Veterinary Authorities (License Number: GZ: MA 58-527308-2024-7).

Bone marrow-derived macrophages (BMMs) were differentiated from bone marrow isolated from femurs and tibias of 8–12-week-old (B6(Cg)-Ifnar1tm1.2Ees/J (Ifnar1^−/−^, JAX#028288, a kind gift of A. Bergthaler, Medical University of Vienna) mice of both sexes. Femur and tibia marrow were centrifuged, and cells were resuspended in DMEM. Red blood cells were lysed using ACK lysis buffer (Thermo Fisher), and cells were differentiated in BMM medium (DMEM High Glucose medium supplemented with L-glutamine, sodium pyruvate, penicillin–streptomycin (Thermo Fisher), and 10% (v/v) FCS (Gibco) and 30% L-929-conditioned medium (a kind gift from P. Kovarik, University of Vienna). Cells were cultured at 37 °C and 5% CO_2_ in a humidified incubator.

For nucleofection of Cas9 ribonucleoprotein complexes (Cas9:RNPs) Alt-R™ tracerRNA (trRNA), Alt-R™ crisprRNAs (crRNA), Cas9 electroporation enhancer, and Alt-R™ S.p. Cas9 Nuclease V3 were obtained from IDT. For each nucleofection, 400 pmol trRNA and 400 pmol crRNA (2 per gene) were combined and heated to 95 °C for 5 min before slowly cooling to room temperature. Annealed guideRNAs (gRNA) were incubated with 26 µg Cas9 nuclease for 20 min and 1 µl electroporation enhancer was added per reaction. A complete list of crRNAs used in this study can be found in Dataset EV3. Since only one pair of gRNAs was used per gene-targeting approach, off-targets of these gRNAs cannot be excluded.

BMMs were detached on day 5 or 8 of differentiation using cold PBS + 4 mM EDTA. 2 million cells per nucleofection were spun down and resuspended in 20 µl primary cell nucleofector solution P3 supplemented with supplement 1 (Lonza). Cell suspension was combined with Cas9:RNPs and transferred to a nucleofector cassette (Lonza). Cells were nucleofected using the Lonza 4D Nucleofector with the CM-137 program. After nucleofection, cells were resuspended in warm BMM medium and cultured for 1–5 days before analysis.

### Cell stimulation

Individual wells of indicated genotypes were randomized for stimulation. Unless otherwise indicated, MORC3-AID and DAXX-AID were degraded with 1 µM 5Ph-IAA (Bio-Academia) for 24 h, PU.1-dTAG was degraded with 500 nM dTAGV1 (Tocris Bioscience) for 25 h (1 h pre-treatment compared to 5Ph-IAA), 5 µg/ml doxycycline was added to induce PU.1-dTAG expression at day 0 of trans-differentiation, and 10 nM of the STING agonist diABZI (Invivogen) was added for 3 h. Where indicated, BLaER1 cells were stimulated with 200 ng/ml of LPS (Invivogen) or 5 µg/ml of R848 (Invivogen) for 4 h before harvest on the last day of trans-differentiation. 1 µg/ml doxycycline was added for 48 h to induce ectopic PU.1 or mCherry gene expression in HCT-116 and HT-1080 cells. BMMs were stimulated with 100 nM diABZI for 4 h. Researchers were not blinded during data collection; no data were excluded for analysis.

### Quantification of gene expression

Gene expression was analyzed by quantitative PCR with reverse transcription. Total RNA was isolated using an in-house RNA isolation kit based on silica-coated magnetic beads (VBC-MTD core facility). 0.5-1.5 µg RNA was subjected to DNase digestion with DNaseI (NEB) according to the manufacturer’s instructions. For some experiments, mRNA was isolated by lysing cells in lysis buffer (4 M Urea, 0.1 M TRIS (pH 8.0), 0.5 M NaCl, 10 mM EDTA, 1% SDS, 5 mM DTT). Lysate was mixed with 10 µl oligo-dT beads (VBC-MTD core facility) and incubated for 10 min at RT before capturing the beads with a magnet. Beads were washed once while on the magnet with wash buffer (7 mM Tris (pH 8.0), 0.17 M NaCl) and RT buffer (200 mM TRIS pH 8.3, 500 mM KCl, 50 mM MgCl_2_, 200 mM (NH_4_)_2_SO_4_, 1% Triton-X100) and resuspended in RT buffer. DNase-treated total RNA or bead-bound mRNA was used as input for reverse transcription using Superscript III (VBC Protein Technologies core facility). Quantitative PCR was performed with Taq-master mix (VBC MTD core facility) and analyzed with a real-time PCR system (BioRad, CFX Touch). A complete list of primers used for qPCR can be found in Dataset EV3. All gene expression values were normalized to the indicated housekeeping genes and quantified by the 2^-dCt^ method.

### Genomic qPCR

To quantify the integration frequency of luciferase reporter constructs, BLaER1 monocytes were lysed in lysis buffer (0.2 mg/ml proteinase K, 1 mM CaCl_2_, 3 mM MgCl_2_, 1 mM EDTA, 1% Triton X 100, 10 mM Tris pH 7.5) and incubated at 65 °C for 10 min and then at 95 °C for 15 min. The resulting lysate was used to perform quantitative PCR as described for gene expression analysis.

### Generation of MRE reporter cells

DNA sequences for the 1× consensus MRE, control, and mutant repeat units were synthesized by TWIST Bioscience. For multi-repeat cloning, staggered oligos were used to amplify the repeat units via PCR using the Phusion High-Fidelity PCR Kit (Thermo Fisher). PCR products were assembled into a Sleeping Beauty transposase vector upstream of a miniCMV promoter and a Gaussia princeps luciferase gene via golden gate cloning with Esp3I and T4 DNA ligase (both NEB). The vector also contained a puromycin resistance cassette followed by P2A and a *Photinus pyralis*-derived Firefly luciferase. For the SPI motif mutation reporter, all instances of SPI motifs detected by FIMO and Enformer analysis of the MRE repeat consensus sequence (GCTTCTCCTTTT, GCTTCTCATTTC, ACTTCCCCCTTC, GCTTCTCCTTTT, GTTTCTCCTTTC, CTTCCCCATTTT) were mutated by replacing them with different sequences of the same length from an inactive control sequence. For the sufficiency reporter, a random non-repetitive control sequence and the same sequence in which 35x CTTCCCCATTTT motifs were randomly inserted were synthesized by TWIST Bioscience and cloned into the reporter plasmid. All reporters used in this study were sequence verified by long-read sequencing with an external company (Plasmidsaurus). BLaER1 cells were electroporated with the reporter plasmid and pCMV(CAT)T7-SB100 (addgene #34879) and selected with puromycin (Invivogen) 48 h after electroporation. Monoclones or bulk reporter cell populations were transduced with sgRNA-expressing lentiviruses. A complete list of sgRNAs used in this study can be found in Dataset EV3.

### Lentiviral transduction

Lentivirus was produced in LentiX 293T cells. A total of 5 million cells were plated per 10-cm dish and transfected the next day with 5 μg transfer vector, 3.75 μg of packaging vector (pd8.9), and 1.5 μg pVSVG using 30.75 μg PEI-MAX (Polysciences). Twelve hours after transfection, the medium was replaced with DMEM containing 30% (v/v) FCS. After 24–36 h, viral supernatants were collected, centrifuged at 1000× *g* for 10 min, filtered through a 0.45μm filter, and applied to cells. Cells were cultured for 48 h after transduction prior to selection with puromycin, blasticidin S hydrochloride (Invivogen), or geneticin (G418; Gibco).

### CRISPR Cas9-mediated gene targeting using lentiviruses

Unless otherwise indicated, monoclonal gene knockout and MRE deletion BLaER1 cells were generated as described previously (Gaidt et al, 2021b). For polyclonal knockout of genes, cells were transduced with dual-sgRNA cassette containing lentivirus (hU6-sgRNA1-mU6-sgRNA2-EF1αs-FP-Res; FP = gene for a fluorescent protein, Res = antibiotic resistance gene). Control sgRNAs were targeting the AAVS1 locus (intron 1 of *PPP1R12C*). A complete list of sgRNAs used in this study can be found in Dataset EV3.

### Endogenous gene tagging of MORC3

The *MORC3* and *DAXX* genes were targeted in BLaER1 (both) and HCT-116 (only *MORC3*) cells for the integration of the miniAID tag (Kubota et al, 2013) and a triple V5 epitope tag. In all, 2×700 bp homology arms flanking the CRISPR-Cas9 targeting site on the C-terminus were synthesized as gene fragments by TWIST Bioscience and cloned into a modified pEF-BOS backbone (NovoPro Biosciences). The miniAID-3xV5-P2A sequence followed by the hygromycin B or neomycin resistance gene was cloned in between the two homology arms. The sgRNA targeting the C-terminus of each gene was cloned into a minimal plasmid containing U6-sgRNA-CMV-mCherry-T2A-Cas9 (Gaidt et al, 2021b). The two homology donor plasmids, one with hygromycin B and the other with neomycin cassettes, and the sgRNA plasmid were electroporated into BLaER1 cells using a BioRad GenePulser device (265 V; 975 µF; 700 ohm). Cells were sorted for mCherry expression 48 h after electroporation and selected with Geneticin (G418; Gibco) and hygromycin B (Invivogen). For tagging of MORC3 in HCT-116 cells, cells were transfected with the homology donor plasmids and the sgRNA plasmid using Lipofectamine2000 (Invitrogen) according to the manufacturer’s instructions. Cells were transduced with pRRL-TIR1^F74G^-P2A-hCD2 lentivirus and sorted for hCD2 expression. For BLaER1 cells, Cas9 was introduced via transduction with lentiCas9-Blast (Addgene plasmid 52962), and cells were selected with Blasticidin (Invivogen). Finally, clones were genotyped by Sanger sequencing of allele-specific amplicons using hygromycin- or neomycin-specific primers. The primers used for genotyping can be found in Dataset EV3.

### Ectopic gene expression in HCT-116 and HT-1080 cells

PU.1 cDNA, encoded by the *SPI1* gene, was ordered as a gene fragment from TWIST Bioscience. PU.1 and mCherry cDNA were cloned into the doxycycline-inducible lentiviral expression vector pLIP via NheI and BamHI cut sites. HCT-116 MORC3^AIDV5/AIDV5^ and HT-1080 iCas9 cells were transduced with dox-inducible PU.1 or mCherry lentivirus and selected with Puromycin. HT-1080 iCas9 cells were further transduced with sgCTRL- or sgMORC3-expressing lentivirus and selected with Neomycin. 1 µg/ml doxycycline was added for 5 days to induce expression of Cas9 and gene editing. For the experiment HT-1080 iCas9 sgCTRL or sgMORC3 cells were plated in medium with or without 1 µg/ml doxycycline for 2 days, and HCT-116 MORC3^AIDV5/AIDV5^ cells were plated with or without 1 µM 5Ph-IAA and 1 µg/ml doxycycline for 2 days.

### Generation of PU.1-dTAG cells

PU.1 cDNA, encoded by the *SPI1* gene, was fused to the dTAG sequence and cloned into the doxycycline-inducible lentiviral expression vector pLIP (Gaidt et al, 2021b). Cas9 *IFNAR1*^–/–^ *IFNAR2*^–/–^ *MORC3*^*AIDV5/AIDV5*^ TIR1^F74G^ BLaER1 cells were transduced with sgSPI1-expressing lentivirus and dox-inducible PU.1-dTAG lentivirus and dual selected. Monoclones that showed doxycycline-dependent trans-differentiation and absence of endogenous PU.1 signal by Immunoblot were used for experiments.

### Immunoblotting

Whole-cell lysates were prepared by lysing cells in 50 mM Tris pH 7.4, 50 mM NaCl, 2 mM MgCl_2_, 0.5% Igepal, and Complete Mini EDTA-free protease inhibitor (Roche) for 20 min on ice. Lysates were centrifuged at 17,000× *g*, and supernatants were kept. Protein content was determined using a Bradford Assay kit (Thermo Fisher). Laemmli SDS sample buffer (Thermo Fisher) was added to the lysates to a final concentration of 1×, and lysates were boiled at 95 °C for 10 min. Proteins were separated with denaturing PAGE (NuPAGE 4-12% Bis-Tris) and transferred to Immobilon-FL PVDF membranes (Thermo Fisher). Membranes were blocked in blocking buffer (TBS, 4% milk powder, 0,05% Tween-20). Primary antibodies were added, and immunoblots were incubated overnight. Appropriate secondary HRP-conjugated antibodies were used. Immunoblots were incubated with ECL^TM^ Prime Reagent (Cytiva) for 5 min imaged using the BioRad ChemiDoc Go platform. A complete list of antibodies used in this study can be found in Dataset EV3.

### Flow cytometry

For Flow Cytometry analysis, cells were harvested in FACS buffer (PBS, 5% FCS, 1 mM EDTA). Live/dead determination was performed by GFP exclusion in BLaER1 as GFP^+^ BLaER1 cells lose GFP expression upon death.

Trans-differentiation efficiency in BLaER1 was assessed by CD11b staining. Differentiation efficiency in bone-marrow-derived macrophages was assessed by Cd11b, F4/80 staining (dilution 1:500 for both antibodies), including Fc-Block (dilution: 1:100). Cells were analyzed using a BD LSRFortessa Flow Cytometer, and analysis was conducted using FlowJo software (v.10.10.0). A complete list of antibodies used in this study can be found in the Reagents and Tools Table.

### Chromatin immunoprecipitation and qPCR/sequencing

BLaER1 monocytes or bone marrow-derived macrophages (BMMs) were washed with PBS and fixed with 1% formaldehyde for 10 min at RT before quenching the reaction with glycine to a final concentration of 0.6 M. BLaER1 cells were lysed directly in 50 mM Tris-HCl pH 8, 10 mM EDTA, and 1% SDS. BMMs were sequentially lysed at 4 °C using LB1 (50 mM Hepes-KOH pH 7.5, 140 mM NaCl, 1 mM EDTA, 10% glycerol, 0.5% IGEPAL, 0.25% Triton X-100), LB2 (10 mM Tris-HCl pH 8.0, 100 mM NaCl, 1 mM EDTA, 0.5 mM EGTA), and LB3 (10 mM Tris-HCl pH 8.0, 100 mM NaCl, 1 mM EDTA, 0.5 mM EGTA, 0.1% sodium deoxycholate, 0.5% N-lauroylsarcosine), with 10 min incubation per buffer under rotation and centrifugation at 1300×*g* between steps. Lysates were sonicated using the Bioruptor (Diagenode) with cycles of 30 s ON/30 s OFF in the high-power mode until a fragment length of 200–500 bp was achieved. Crude chromatin content was determined by UV absorbance at 260 nm. One ChIP in BLaER1 contained 200 µg chromatin for anti-histone ChIP, 400 µg for anti-PU.1 ChIP, and 10 mg for anti-V5 ChIP, whereas 25 µg chromatin was used per ChIP in BMM. Lysates were mixed with dilution buffer to a final concentration of 20 mM TRIS pH 8, 150 mM NaCl, 2 mM EDTA, 1% Triton-X100, and 0.1% SDS. 2.5% Drosophila melanogaster S2 cell-derived chromatin (prepared similarly) was spiked into the samples. Lysates were pre-incubated at 4 °C for 2 h with ProteinA-coated magnetic beads (G&E) previously blocked in TE with 1% BSA (15 µl per reaction). After removal of the beads, target and spike-in antibodies were added to the lysates and incubated at 4 °C overnight (BLaER1: anti-PU.1 antibody: 5 µl/ChIP ( = 40 ng), anti-H3K27ac antibody: 2 µl/ChIP, anti-V5 antibody: 0.5 µg/ChIP, Drosophila spike-in antibody: 0.1 µl/ChIP, Isotype control: 0.5 and 2 µg/ChIP; BMM: anti-PU.1 antibody: 2 µl/ChIP ( = 16 ng), Isotype control: 1 µg/ChIP). In all, 15 µl per fresh blocked beads per reaction were added and incubated with the lysates for 2 h at 4 °C. Beads were washed with RIPA buffer (150 mM NaCl, 50 mM TRIS pH 8, 0.1% SDS, 0.5% Na-deoxycholate, 1% Igepal), high salt buffer (500 mM NaCl, 50 mM TRIS pH 8, 0.1% SDS, 1% Igepal), LiCl buffer (250 mM LiCl, 50 mM TRIS pH 8, 0.5% Na-deoxycholate, 1% Igepal), and TE wash buffer (10 mM TRIS-HCl, 1 mM EDTA, 50 mM NaCl), and protein–DNA complexes were eluted with 100 mM NaHCO_3_ + 1% SDS. ChIP and input samples were incubated overnight at 55 °C with 200 mM NaCl, 10 mM EDTA, 20 mM Tris-HCl pH 6.5, and 300 µg/ml Proteinase K (Thermo Fisher). DNA was isolated using the ChIP DNA Clean & Concentrator kit (Zymo Research). Quantitative PCR was performed with Taq-master mix (VBC MTD core facility) and analyzed with a real-time PCR system (BioRad, CFX Touch). A complete list of primers used for qPCR can be found in Dataset EV3. C_t_ values were normalized to input as 2^^(Ct(input)- Ct(sample))^, and statistical analysis was performed on these normalized data. The dynamic range and linearity of the IFNB1-MRE ChIP-qPCR assay were confirmed by titrating genomic DNA from WT and IFNB1-MRE^–/–^ (no template control) BLaER1 cells in the qPCR. For sequencing, libraries were prepared with the NEBNext Ultra II Library Prep Kit and NEBNext Multiplex Oligos (New England BioLabs) according to the manufacturer’s instructions. Libraries were sequenced on an Illumina NovaSeqX instrument with read-mode PE150.

### Cut&Run-Seq

Anti-H3K4me3 Cut&Run experiment was adapted from (Skene et al, 2018). Briefly, 500000 *IFNAR1*^–/–^ *IFNAR2*^–/–^ *MORC3*^*AIDV5/AIDV5*^ TIR1^F74G^ BLaER1 monocytes per reaction were washed with and resuspended in Wash Buffer (20 mM HEPES pH 7.5, 150 mM NaCl, 0.5 mM Spermidine, Complete Mini EDTA-free Protease inhibitor (Roche)). Bead preparation: 20 µl Streptavidin conjugated magnetic beads per reaction (Dynabeads T1 Streptavidin, Thermo Fisher) were incubated with in ConcavalinA solution (2.3 mg/ml ConcavalinA-Biotin conjugate (Merck) in PBS pH 6.8 + 0.01% Tween-20) for 30 min. Beads were washed with PBS pH 6.8 + 0.01% Tween-20 and then activated with binding buffer (200 mM Hepes pH 7.5, 100 mM KCl, 10 mM CaCl_2_, 10 mM MnCl_2_). 20 µl prepared beads per reaction were incubated with cells for 5 min before removing the buffer and resuspending the bead-bound cells in DigWash Buffer (Wash Buffer + 0.01% Digitonin) containing 2 µl anti-H3K4me3 antibody (C42D8; Cell Signaling Technology) or Isotype control (DA1E; Cell Signaling Technology). Incubation for 2 h at 4 °C. Buffer was removed, and cells were washed three times with DigWash Buffer. Cells were incubated in 150 µl pAG-MNase solution (DigWash buffer + 700 ng/ml protein-AG-MNase, produced in-house by IMP-MBS core-facility) for 1 h at 4 °C. Cells were washed three times with DigWash Buffer and incubated in 100 µl DigWash Buffer containing 2 mM CaCl_2_ for 30 min at 4 °C. 100 µl Stop Buffer (170 mM NaCl, 20 mM EGTA, 0.01% Digitonin, 50 µg/ml RNaseA, 25 µg/ml Glycogen) was added and the mix was incubated for 30 min at 37 °C. Supernatant was transferred to a fresh tube, and DNA was isolated with MinElute spin columns (Qiagen). Libraries were prepared with the NEBNext Ultra II Library Prep Kit and NEBNext Multiplex Oligos (New England BioLabs) according to the manufacturer’s instructions. Libraries were sequenced on an Illumina NextSeq2000 instrument with read-mode PE50.

### Cut&Run-seq and ChIP-seq analysis

All datasets were aligned to GRCh38. Cut&Run data was analyzed with the nf-core/cutandrun pipeline (v3.2.2), with --peakcaller option set to macs2. ChIP data was analyzed with the nf-core/chipseq pipeline (v2.1.0) with default parameters unless otherwise specified. For PU.1 ChIP-seq, counts and coverage data were spike-in normalized due to large variability between replicates. For spike-in normalization, reads were aligned to the Drosophila melanogaster genome (NCBI build 5.41) using Bowtie2 (v2.5.1) and deduplicated using a paired-end–aware Samtools workflow (fixmate and markdup) (v1.17). Deduplicated mapped Drosophila reads were counted for each sample, and a scaling factor was calculated as the ratio of the median Drosophila read count across all samples to the Drosophila read count of each individual sample. These scaling factors were used to normalize target read counts and coverage. Heatmaps for target coverage across indicated regions (features, peaks) were plotted as read-depth normalized read-coverage using the DeepTools suite (v 3.5.6) (Ramirez et al, 2014).

Motif enrichment analysis for motifs within PU.1 ChIP-seq peaks were performed using HOMER findMotifsGenome.pl (Heinz et al, 2010).

Association of genomic regions (e.g., ChIP-seq or ATAC-seq peaks) with genomic features was initially assessed using HOMER annotatePeaks.pl (hg38 v6.0; Heinz et al, 2010). However, Homer annotations for simple repeats (tandem repeats) contain only shorter microsatellites and not the larger minisatellites (VNTRs). To more accurately identify all simple repeat–overlapping regions, we intersected peak coordinates with the UCSC Genome Browser Simple Repeats track using bedtools intersect (v2.30.0). Peaks were classified as simple repeat–overlapping if more than 10% of their length overlapped an annotated simple repeat. This annotation was merged with the Homer annotation for other genomic features for the feature distribution analysis, assigning tandem repeats the highest priority.

Overlaps between peak sets from different conditions (e.g., ChIP-seq for different targets, ChIP-seq versus ATAC-seq, or the same ChIP-seq target ± treatment) were determined using bedtools intersect (v2.30.0) (Quinlan and Hall, 2010). To determine which peaks change upon treatment (e.g., MORC3 peaks lost upon PU.1 depletion), we used the MACS3 bdgdiff command (v3.0.1) (Zhang et al, 2008) with a log10 likelihood ratio (LR) cutoff of 3. For the two conditions DMSO and dTAGV1, a peak was defined as DMSO-specific if it met both of the following criteria: (i) the read count in DMSO was significantly higher than in dTAGV1 (differential binding between conditions), and (ii) the read count in DMSO was significantly enriched over the corresponding input control, with log2FC > 2. Log2 fold changes between ChIP-seq conditions (DMSO vs dTAGV1 for MORC3, and DMSO vs 5-Ph-IAA for PU.1) were additionally calculated using DESeq2 (v1.42.1; Love et al, 2014). Briefly, consensus peak sets (for MORC3 or PU.1) were first filtered to remove low-signal peaks (cutoff = 2% quantile of the count distribution; peaks were removed if the total read count was <2× this cutoff). Differential analysis was then performed with DESeq2, using size-factor estimation based on all peaks.

### ATAC-seq

All datasets were aligned to GRCh38. ATAC-seq data from *IFNAR1*^–/–^ *IFNAR2*^–/–^ and *IFNAR1*^–/–^ *IFNAR2*^–/–^ *MORC3*^–/–^ BLaER1 monocytes, Data ref (Gaidt et al, 2021a), was re-analyzed using the nf-core ATAC-seq pipeline (v2.1.2) with default parameters. The obtained ATAC-Seq consensus peaks were filtered for low signals (read count <10 in more than two samples per condition), and differential analysis was performed with DESeq2 (v1.42.1) (Love et al, 2014), with sample size factors estimated using the top 70% of signals. ATAC-seq peaks overlapping with MORC3 ChIP-Seq peaks were determined using bedtools intersect. Association of ATAC-seq peaks with genomic features was assessed as described earlier for MORC3 peaks.

### RNA-Seq

Differential analysis of RNA-Seq data, Data ref (Gaidt et al, 2021a), was performed with DESeq2 (1.44.0).

### Repenrich2

RepEnrich2 (Criscione et al, 2014) was used to quantify accessibility at ERVs. The MAPQ threshold for subsetting uniquely mapping and multimapping reads in RepEnrich2 was set to 30 as recommended for alignment with bowtie2. We use pre-built repeat annotations for RepEnrich2, available through the RepEnrich2 GitHub (https://github.com/nerettilab/RepEnrich2). We analyzed differential enrichment of ERV counts from RepEnrich2 using DeSeq2 version 1.44.0. No ERVs were found to be significantly differentially accessible based on FDR < 0.05.

### Danbing-tk analysis of VNTR-associated ChIP-seq reads

Anti-V5 (MORC3) ChIP-seq reads (150PE) were analyzed with Danbing-tk (Lu et al, 2021; Lu et al, 2023) with the parameters -qc qc.bool.txt -b -qs pan, and the provided repeat-pan genome graphs (https://github.com/ChaissonLab/danbing-tk). Danbing-tk employs de Bruijn graph-based representation of VTNRs for faithful read assignment. To benchmark conventional short-read mapping against Danbing-tk, the same dataset was processed using the nf-core ChIP-seq pipeline (v2.1.0), which uses the BWA aligner (0.7.17). In this analysis, duplicated reads and multimappers were retained. Read counts over danbing-tk-defined VNTR regions were generated using featureCounts (subread version 2.0.2) (Liao et al, 2014). All analyses were implemented using Snakemake version 9.5.1 (Molder et al, 2021). For a direct comparison, reads that failed Danbing-tk’s internal quality control were excluded from both pipelines. The resulting read counts showed good agreement between Danbing-tk and BWA-based mapping (Fig. EV2A), indicating that BWA can reliably assign 150 bp paired-end reads to VNTR regions, likely owing to the increased mappability conferred by read length. Because Danbing-tk does not perform duplicate removal, BWA outputs without deduplication were used for comparison. Multimapping reads were initially retained, as BWA’s random assignment of multimappers may be particularly suitable for VNTRs, specifically in cases where ambiguity arises from similarity between repeat units that belong to the same VNTR rather than from distinct genomic locations. Consistent with this, direct comparison of multimapping reads showed that both BWA and Danbing-tk assigned them to the same VNTR (Fig. EV2B), indicating that these reads contain biologically meaningful information. Nevertheless, inclusion of multimappers in downstream analyses would also introduce noise from reads originating outside VNTRs. Therefore, although we consider VNTR-associated multimappers to be informative, we excluded multimapping reads from downstream analyses to minimize potential confounding effects.

### DNase hypersensitivity prediction using Enformer

DNase hypersensitivity predictions were performed using the Enformer model (Avsec et al, 2021a) following the implementation available at: https://github.com/bernardo-de-almeida/Enformer_predictions. The Enformer model predicts DNase-seq signal for any given input DNA sequence in user-specified cell types. The following cell types were utilized: ID 1—ENCODE: ENCFF110QGM —Cell type: frontal cortex; ID 23—ENCODE: ENCFF148BGE—Cell type: HEK293T; ID 85—ENCODE: ENCFF484UXW—Cell type: cardiac muscle cell; ID 131—ENCODE: ENCFF659BVQ—Cell type: CD14+ monocyte 1; ID 353—ENCODE: ENCFF259NLK—Cell type: CD1c+ myeloid dendritic cell; ID 392— ENCODE: ENCFF724HAH—Cell type: CD14+ monocyte 2. Specifically, a 196,608 bp sequence corresponding to chr9:20875792 -21072401 of the human hg38 reference genome was used as input to Enformer. Predictions generated DNase-seq values across the central 896 bins, each 128 bp in size, out of the total of 1536 bins, producing one numeric value per bin. Predicted DNase-seq values obtained for each cell type were transformed into bedgraph files and are visualized in Figs. 4A and EV5A.

To identify nucleotide positions critical for DNase hypersensitivity at the IFNB1-MRE, the seven core repeats within the IFNB1-MRE region (chr9:20973425-20974769) were replaced by a sevenfold concatenation of their consensus sequence, preserving the original genomic context. This replacement did not alter Enformer predictions, maintaining monocyte-specific DNase hypersensitivity (Fig. EV5A). Subsequently, in silico saturation mutagenesis was performed, where each nucleotide position within the 192 bp consensus sequence was individually mutated to each of the three alternative nucleotides. Each mutant sequence was concatenated seven times and placed into the original genomic context, generating a total of 576 unique mutants (192 positions × 3 alternative bases per position). DNase-seq signals were predicted for all mutants and the WT sequence. To quantitatively compare DNase-seq signals at the IFN-MRE between WT and mutants, signals were averaged across the 18 bins overlapping this region. At each nucleotide position within the 192 bp consensus sequence, the maximum difference in DNase-seq signal between WT and the three mutants was determined. These maximum differences were visualized as a sequence logo displaying the WT nucleotide at each respective position.

### BPNet model-based analysis

BPNet models were trained using the bpnet-lite GitHub repository (v0.8.0) (https://github.com/jmschrei/bpnet-lite) and ATAC-seq data from *IFNAR1*^–/–^ *IFNAR2*^–/–^ and *IFNAR1*^–/–^ *IFNAR2*^–/–^ *MORC3*^–/–^ BLaER1 monocytes, Data ref (Gaidt et al, 2021a). To capture the full length of the repeat elements, these models had an expanded input window size of 4228 bp (double the standard 2114 bp) and output window size of 2000 bp and, accordingly, used 9 dilated residual layers instead of 8. Each convolution layer in the model had 256 filters. They were trained on the concatenation of peak and GC-matched negative regions in both the wild-type and knockout settings. When training, we used a batch size of 32, an alpha of 100, a maximum jitter of 128 bp, and trained at half precision with bfloat16 with the standard learning rate of 0.001 for an AdamW optimizer. During training, a random 50% of sequences were reverse-complemented, and their corresponding bp-resolution profiles were reversed. These models were trained for 50 epochs, and the model with the best validation set performance was kept.

Post-training analysis of the model predictions was performed using the tangermeme Python library (v0.4.4).

#### Attribution analysis

The contribution of individual nucleotides to the overall accessibility prediction for each analyzed region was determined using DeepLIFT/SHAP. In contrast to the in silico saturation mutagenesis approach used for Enformer, this method explains a prediction by comparing the input sequence to reference sequences. We used 20 dinucleotide-shuffled versions of an input sequence as its references to disrupt motif content while maintaining dinucleotide composition. The difference between the model’s output for the input and the reference sequence is backpropagated through the neural network. This process assigns attribution scores to each input nucleotide based on how much it contributed to the difference, such that the sum of these scores across nucleotides is equal to the difference in predictions between the input and reference sequence. Sequence segments with high attribution (seqlets) were determined using the tangermeme TF-MoDisco-based seqlet-calling function (parameters: window_size=10, flank=1, target_fdr=0.2, min_passing_frac=0.00, max_passing_frac=1.0). 12 bp seqlets were annotated with transcription factor binding motifs using the TOMTOM-based annotate seqlets function (tangermeme) with the JASPAR2024_CORE_vertebrates_non-redundant motif database and a motif *P* value threshold of 0.01. Only motifs with a minimal length of seven nucleotides were considered for the annotation. Motifs were further filtered for a high attribution score ( > 0.01). Motif abundance was determined by counting the number of non-overlapping matches in the respective MORC3 peak. Counts from different motifs of the same transcription factor were summarized. Only motifs that occurred ≥5 times in a peak were considered for the marginalization analysis.

#### Marginalization analysis

Marginalization analysis was performed by substituting 1–35 copies of a motif of interest into 20 randomly sampled genomic background sequences (derived from chr 12) with a motif spacing of 20 nucleotides and predicting accessibility for the WT and *MORC3*^*–/–*^ model. For each motif and copy number predictions over all 20 sequences were averaged and the mean of the predictions for the background sequences was subtracted. To estimate the impact of a motif cluster (Fig. EV7G), all motifs in a tandem repeat were mutated using the ersatz.shuffle command from tangermeme with *n* = 1 shuffle per motif. This command substitutes each motif occurrence with a shuffled version of that motif to maintain nucleotide content. Accessibility was predicted for each mutated sequence in the *MORC3*^–/–^ and wild-type model. The difference in accessibility between the *MORC3*^–/–^ and wild-type models (MORC3-mediated restriction) for each mutant sequence was calculated as the percentage of the restriction of the wild-type sequence.

### Analysis of the repeat number distribution across the human population

Multiallelic genotyped structural variations (Schloissnig et al, 2025) were used to reconstruct the IFNB1-MRE haplotypes of 967 individuals. These variations either added or removed full copies of the repeat unit. The number of repeat units observed in each haplotype was then compared to the reference sequence (CHM13 T2T assembly) to determine the deviation in unit count.

### Public datasets

ATAC-seq data from *IFNAR1*^–/–^ *IFNAR2*^–/–^ and *IFNAR1*^–/–^ *IFNAR2*^–/–^ *MORC3*^–/–^ BLaER1 monocytes, Data ref (Gaidt et al, 2021a), and RNA-seq counts from *IFNAR1*^–/–^ *IFNAR2*^–/–^, *IFNB1*^–/–^, Cas9 *STAT1*^–/–^ *STAT2*^–/–^, Cas9 *STAT1*^–/–^ *STAT2*^–/–^ IFN-MRE^–/–^, *IFNAR1*^–/–^ *IFNAR2*^–/–^ *MORC3*^–/–^, *IFNB1*^–/–^ *MORC3*^–/–^, Cas9 *STAT1*^–/–^ *STAT2*^–/–^ *MORC3*^–/–^ and Cas9 *STAT1*^–/–^ *STAT2*^–/–^ IFN-MRE^–/–^ *MORC3*^–/–^ BLaER1 monocytes, Data ref (Gaidt et al, 2021a), were analyzed. Repeat Masker and simple repeat tracks were downloaded from the UCSC genome browser for the GRCh38 genome assembly. The following ENCODE datasets were used: ENCFF110QGM, ENCFF148BGE, ENCFF484UXW, ENCFF659BVQ, ENCFF259NLK, ENCFF724HAH. ChIP-Seq data for PU.1 in primary human monocytes were from GSE128837, Data ref: (Minderjahn et al, 2019), (datasets: GSM3686920-GSM3686923; GSM3686932-GSM3686933; GSM3686949-GSM3686955).

### Motif comparison and motif scanning

For identification of transcription factor binding sites within MREs, the FIMO tool (MEME Suite) was used with the default parameters and the JASPAR2022_CORE_redundant_v2 database (Grant et al, 2011). For motif comparison of Enformer predicted important nucleotides against known transcription factor binding motifs, the TOMTOM tool (MEME Suite) was used with the default parameters and using the Jaspar_Vertebrates and Uniprobe_mouse database (Gupta et al, 2007).

### Statistical analysis

Data were analyzed for statistically significant differences using GraphPad Prism 10 and R. Gene expression values were log2-transformed for statistical analysis. Statistical tests are indicated in the figure legends and were repeated measures one-way or two-way ANOVA and Dunnett’s or Bonferroni’s post hoc test, paired, two-sided *t* test, unpaired, two-sided Wilcoxon rank-sum (Mann–Whitney *U*) test, Kolmogorov–Smirnov test, or Wald test as part of the DeSeq2 package. Normality of gene expression data was tested using a D’Agostino and Pearson test. Group sizes were estimated using published data from comparable assays (Gaidt et al, 2021b) to ensure sufficient power to detect statistically significant differences. *P < 0.05; **P < 0.01; ***P < 0.001. The factors for the two-way ANOVA were the two variables of the experimental conditions, e.g., sgRNA and reporter construct.

### Identification of IFN-MRE sequences and determination of consensus sequences

To identify Ifn-Mre loci in other species, the adjacent 3 kb regions from hg38 were blasted against the respective genomes. The top blast hit was manually checked to lie within the *Focad* gene near an *Ifn* locus, and then analyzed with the Tandem repeats finder algorithm (Benson, 1999). The identified genomic repeat region was analyzed for occurrences of Spi transcription factor binding motifs.

### Multiple sequence alignment of the MRE consensus across species

Tandem repeats finder derived consensus sequences for the MRE from different species were aligned using Clustal Omega (Sievers et al, 2011) with default parameters and visualized with Jalview (version 2.11.4.1)(Waterhouse et al, 2009).

### Analysis of SPI-motif clusters in the human genome

Motif occurrences from the JASPAR TFBS genomic tracks for matrix profiles MA0080.7, MA0081.3, and MA0687.2 were grouped into clusters if neighboring motifs were separated by a maximum distance of 200 bp. Motifs overlapping by >= 4 bp were merged into one motif. Only clusters containing more than 15 non-overlapping motifs were retained for downstream analysis. Chromatin accessibility and PU.1 binding at these clusters were assessed using ATAC-seq and anti-PU.1 ChIP-seq data from wild-type and MORC3-depleted BLaER1 cells. Regions were classified as closed chromatin if ATAC-seq signal was below a basemean of 10 (mean of normalized counts across all samples) and as PU.1-bound if ChIP-seq signal exceeded a fivefold enrichment over input at the overlapping PU.1 peak.

### Use of large language models in writing

ChatGPT and Claude were used for language and spelling checking, and to make the manuscript more concise. The wording was reviewed and edited.

## Supplementary information

Peer Review File

Dataset EV1

Dataset EV2

Dataset EV3

Source data Fig. 1

Source data Fig. 2

Source data Fig. 3

Source data Fig. 4

Source data Fig. 5

Source data Fig. 6

Source data Fig. 7

EV Figure Source Data

Expanded View Figures

## Data Availability

Material is available upon request from the corresponding author. All datasets are included in this manuscript or available in the following databases: ChIP-seq and Cut&Run-seq data: Gene Expression Omnibus GSE301805. BPNet models: Zenodo (10.5281/zenodo.18922628). The source data of this paper are collected in the following database record: biostudies:S-SCDT-10_1038-S44318-026-00799-9.
