# Supplementary material for: MORC3 represses a tandem repeat enhancer to regulate interferon
Source: EMBO J. 2026 Jun 5;45(14):5130–63. doi: 10.1038/s44318-026-00799-9 (PMC13373173; doi:10.1038/s44318-026-00799-9)
Supplement: Supplementary file 13 — Expanded View Figures [file 44318_2026_799_MOESM13_ESM.pdf]

Expanded View Figures

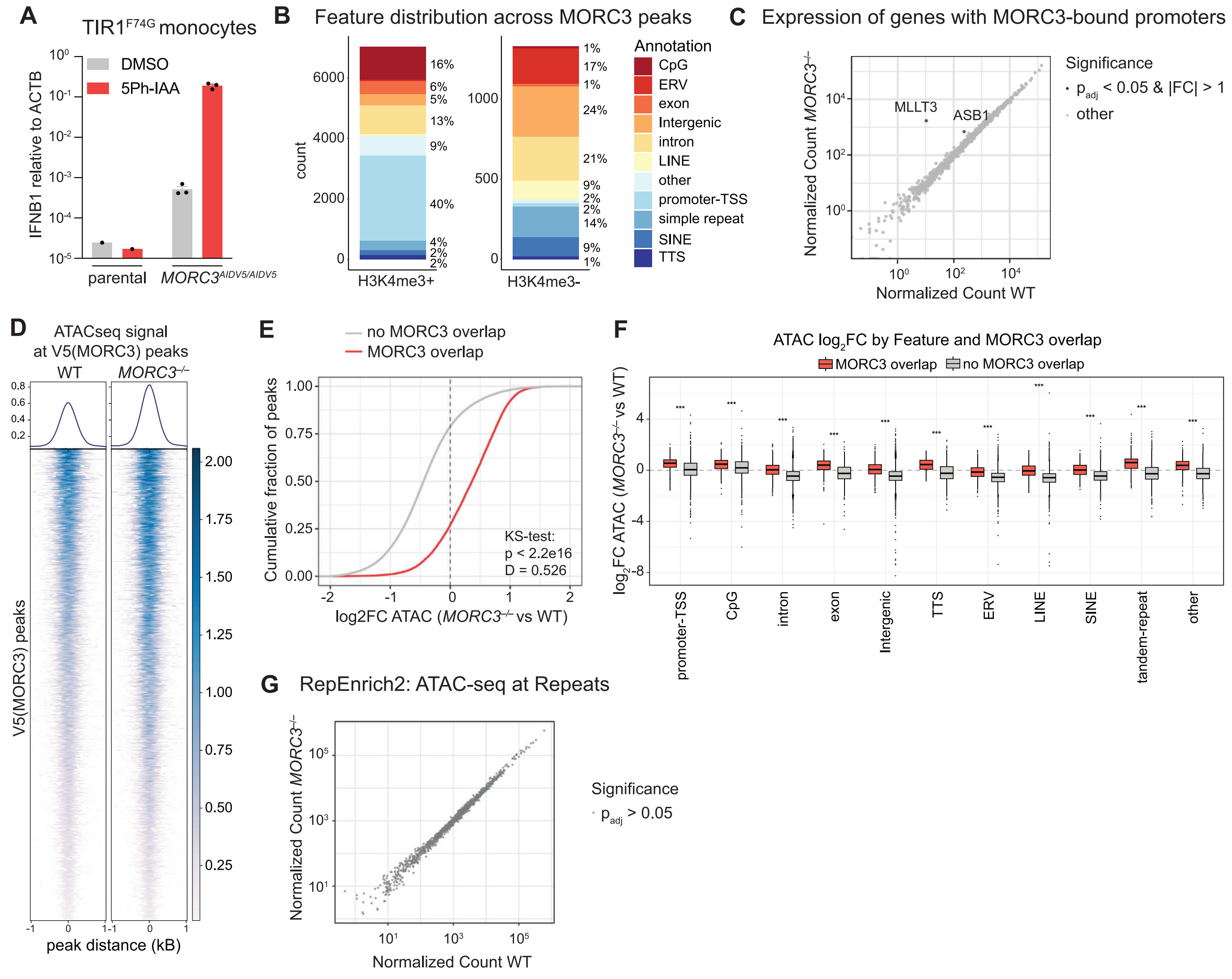

Figure EV1. MORC3 does not repress gene expression in monocytes at bound promoters.

(**A**) *IFNB1* gene expression in $IFNAR1^{-/-}$ $IFNAR2^{-/-}$ TIR1^{F74G} or $IFNAR1^{-/-}$ $IFNAR2^{-/-}$ $MORC3^{AIDV5/AIDV5}$ TIR1^{F74G} BLaER1 monocytes (parental or $n = 3$ independent monoclones) treated with 5Ph-IAA or DMSO for 24 h. (**B**) Genomic feature annotation at MORC3-V5 peaks stratified into H3K4me3 overlapping and non-overlapping groups. Relative abundance was rounded to the next integer and is shown for features ≥1%. (**C**) Expression of genes with MORC3-bound promoters in BLaER1 monocytes comparing WT and $MORC3^{-/-}$ conditions ($n = 10$). Data from GSE183011 included $IFNAR1^{-/-}$ $IFNAR2^{-/-}$, $IFNB1^{-/-}$, Cas9 $STAT1^{-/-}$ $STAT2^{-/-}$ and Cas9 $STAT1^{-/-}$ $STAT2^{-/-}$ MRE$^{-/-}$ monocytes, and $MORC3^{-/-}$ matched conditions. *MLLT3* is in the vicinity of the IFNB1-MRE, and *ASB1* is in the vicinity of the TWIST2-MRE. *P* values were calculated as part of DESeq2 using the Wald test and adjusted for multiple testing (P_{adj}) using the Benjamini–Hochberg method. (**D**) Read-density heatmap of ATAC-seq signal at MORC3-V5 peaks comparing $IFNAR1^{-/-}$ $IFNAR2^{-/-}$ and $IFNAR1^{-/-}$ $IFNAR2^{-/-}$ $MORC3^{-/-}$ BLaER1 monocytes. (**E, F**) Cumulative differential analysis and differential analysis at annotated genomic feature regions of ATAC-seq signal at ATAC-seq peaks overlapping or non-overlapping with MORC3-V5 peaks comparing $IFNAR1^{-/-}$ $IFNAR2^{-/-}$ and $IFNAR1^{-/-}$ $IFNAR2^{-/-}$ $MORC3^{-/-}$ BLaER1 monocytes. Data is from GSE183011. Data were analyzed by the Kolmogorov–Smirnov (KS) test in (**E**) or the Wilcoxon rank-sum test and Bonferroni's post hoc test in (**F**). Promoter-TSS, $P = 8.88E-264$; CpG, $P = 8.63E-30$, intron, $P = 0.00E+00$; exon, $P = 5.29E-70$; Intergenic, $P = 0.00E+00$; TTS, $P = 4.37E-46$; ERV, $P = 3.45E-78$; LINE, $P = 4.09E-88$; SINE, $P = 4.36E-37$; tandem-repeat, $P = 4.62E-165$; other, $P = 3.68E-170$. The center line of the boxplots in (**F**) indicates the median (50th percentile). The box bounds mark the first (Q1, 25th percentile) and third quartiles (Q3, 75th percentile). The whiskers extend to the most extreme data points within 1.5 times the interquartile range (IQR = Q3–Q1) from the quartiles. (**G**) Differential analysis of ATAC-seq signal at interspersed repeat elements using Repenrich2 comparing $IFNAR1^{-/-}$ $IFNAR2^{-/-}$ and $IFNAR1^{-/-}$ $IFNAR2^{-/-}$ $MORC3^{-/-}$ BLaER1 monocytes. Data are from GSE183011. *P* values were calculated as part of DESeq2 using the Wald test and adjusted for multiple testing (P_{adj}) using the Benjamini–Hochberg method. Unless otherwise indicated, data are from $n = 3$ independent experiments. Source data are available online for this figure.

© The Author(s)

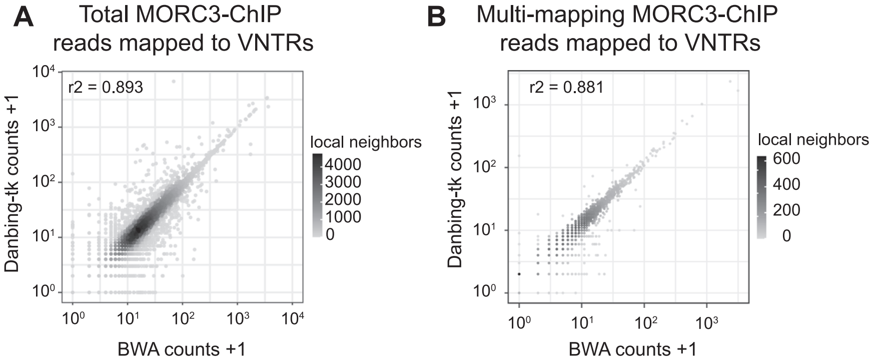

Figure EV2. Comparison of BWA and Danbing-tk in mapping short reads to VNTRs.

Total **(A)** or multi-mapping **(B)** anti-V5 (MORC3) ChIP-seq reads from a single replicate were mapped to VNTRs using the conventional BWA aligner or Danbing-tk, a computational tool specialized in assigning reads to VNTRs (see "Methods"). Source data are available online for this figure.

 © The Author(s)

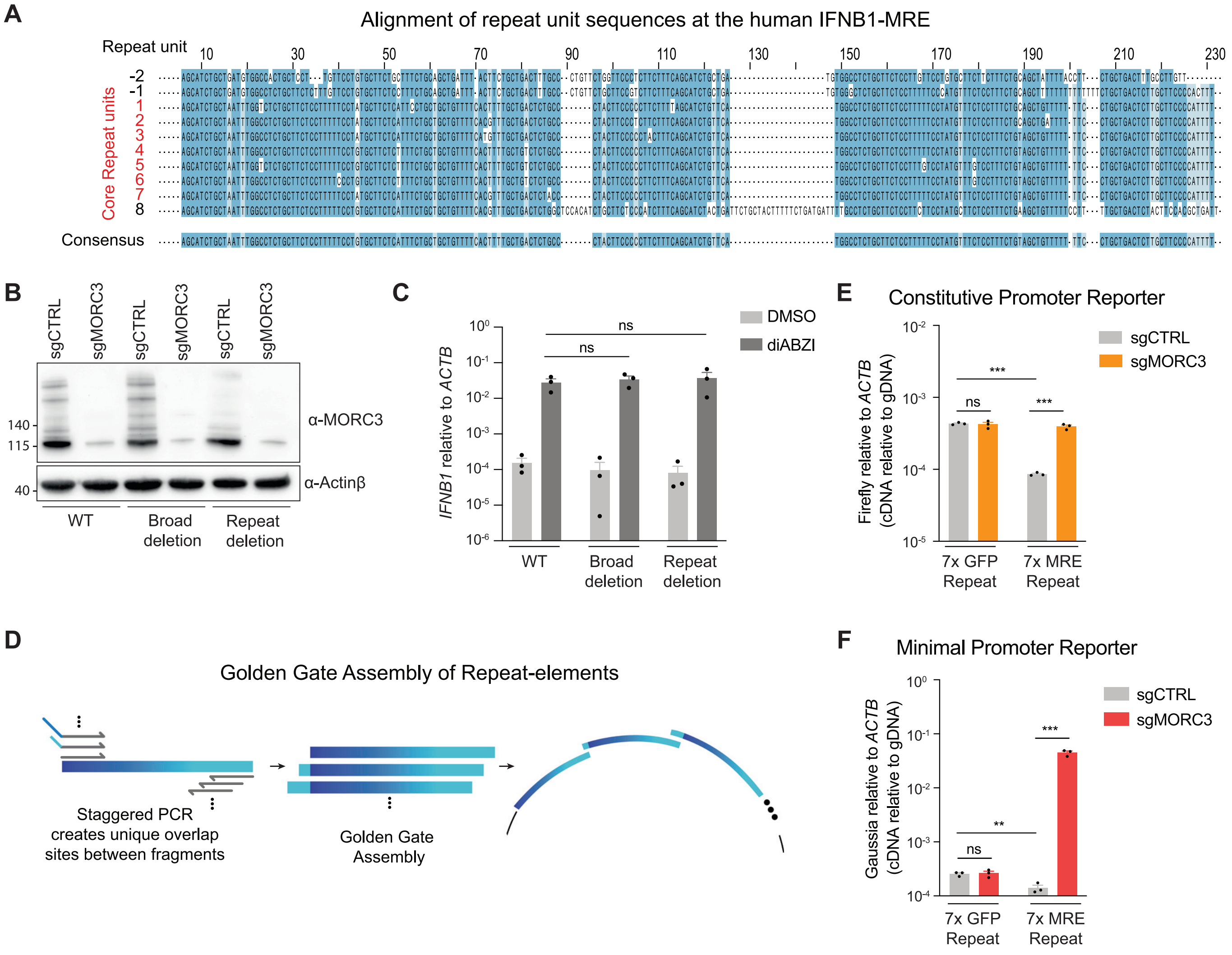

Figure EV3. The IFNB1-controlling MRE tandem repeat.

(**A**) The consensus sequence of the repeat units at the MRE locus of the hg38 reference genome was determined using the Tandem Repeats Finder algorithm. Sequences of individual repeat units were aligned to the consensus sequence using Clustal Omega. Core repeat units with >95% identity to the consensus sequence are in red. MRE = MORC3-repressed element. (**B**, **C**) The remaining MRE allele in Cas9 *STAT1*$^{-/-}$ *STAT2*$^{-/-}$ MRE$^{-/+}$ BLaER1 monocytes was edited to induce a broad deletion or a specific repeat deletion. One representative immunoblot of two upon lentiviral sgRNA delivery and *IFNB1* expression after stimulation with the STING agonist diABZI is shown. Ns, $P > 0.9999$. (**D**) Golden Gate repeat element assembly. Staggered PCR strategy using a synthetic consensus/mutant variant as template generates unique sticky ends between fragments. (**E**, **F**) Seven concatenated consensus IFNB1-MRE repeat units, or control GFP-derived repeat units of the same length, were inserted into the luciferase reporter and integrated into the genome of Cas9 *IFNAR1*$^{-/-}$ *IFNAR2*$^{-/-}$ MRE$^{-/-}$ BLaER1 cells. Constitutive Promoter Firefly luciferase and Minimal Promoter Gaussia luciferase gene expression upon lentiviral sgRNA delivery was determined by qPCR on reverse transcribed cDNA and normalized to qPCR on genomic DNA. $^{**}P = 0.0079$; $^{***}P < 0.0001$; ns, $P > 0.9999$. Data are shown as mean + SEM from $n = 3$ independent experiments. Significance of differences was determined by two-way ANOVA and Bonferroni's post hoc test. Source data are available online for this figure.

© The Author(s)

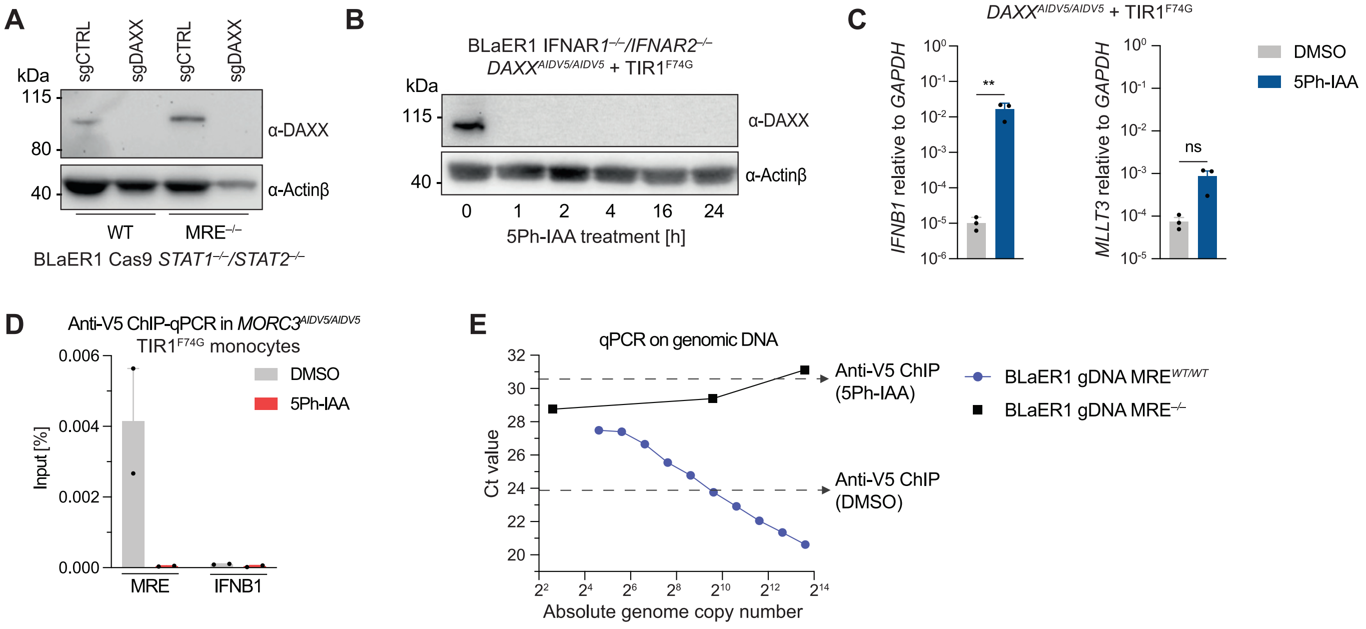

Figure EV4. Validation of DAXX deletion and ChIP-qPCR at the MRE.

(**A**, **B**) Immunoblot analysis in BLaER1 monocytes of the indicated genotype after lentiviral delivery of sgRNAs or treatment with 5Ph-IAA to degrade DAXX. One representative immunoblot of two is shown. (**C**) Gene expression analysis in *IFNAR1*$^{-/-}$ *IFNAR2*$^{-/-}$ *DAXX*$^{AIDV5/AIDV5}$ TIR1^{F74G} BLaER1 monocytes after treatment with 5Ph-IAA for 24 h. Data is shown as mean + SEM from $n = 3$ independent experiments. $^{**}P = 0.0062$; ns, $P = 0.0723$ as determined by paired, two-sided *t* test. (**D**) Anti-V5 ChIP-qPCR analysis in *IFNAR1*$^{-/-}$ *IFNAR2*$^{-/-}$ *MORC3*$^{AIDV5/AIDV5}$ TIR1^{F74G} BLaER1 monocytes that were treated with 5Ph-IAA for 24 h is depicted as mean + SEM of $n = 2$ independent experiments. The IFNB1 locus (gene body) serves as a negative control where MORC3 does not bind. (**E**) Linearity and dynamic range of qPCR-based quantification of MRE-repeat DNA. Ct values from qPCR with purified genomic DNA from WT or MRE$^{-/-}$ BLaER1 cells are depicted from one experiment. Arrows indicate raw Ct values from the anti-V5 ChIP-qPCR experiment in (**D**). Source data are available online for this figure.

 © The Author(s)

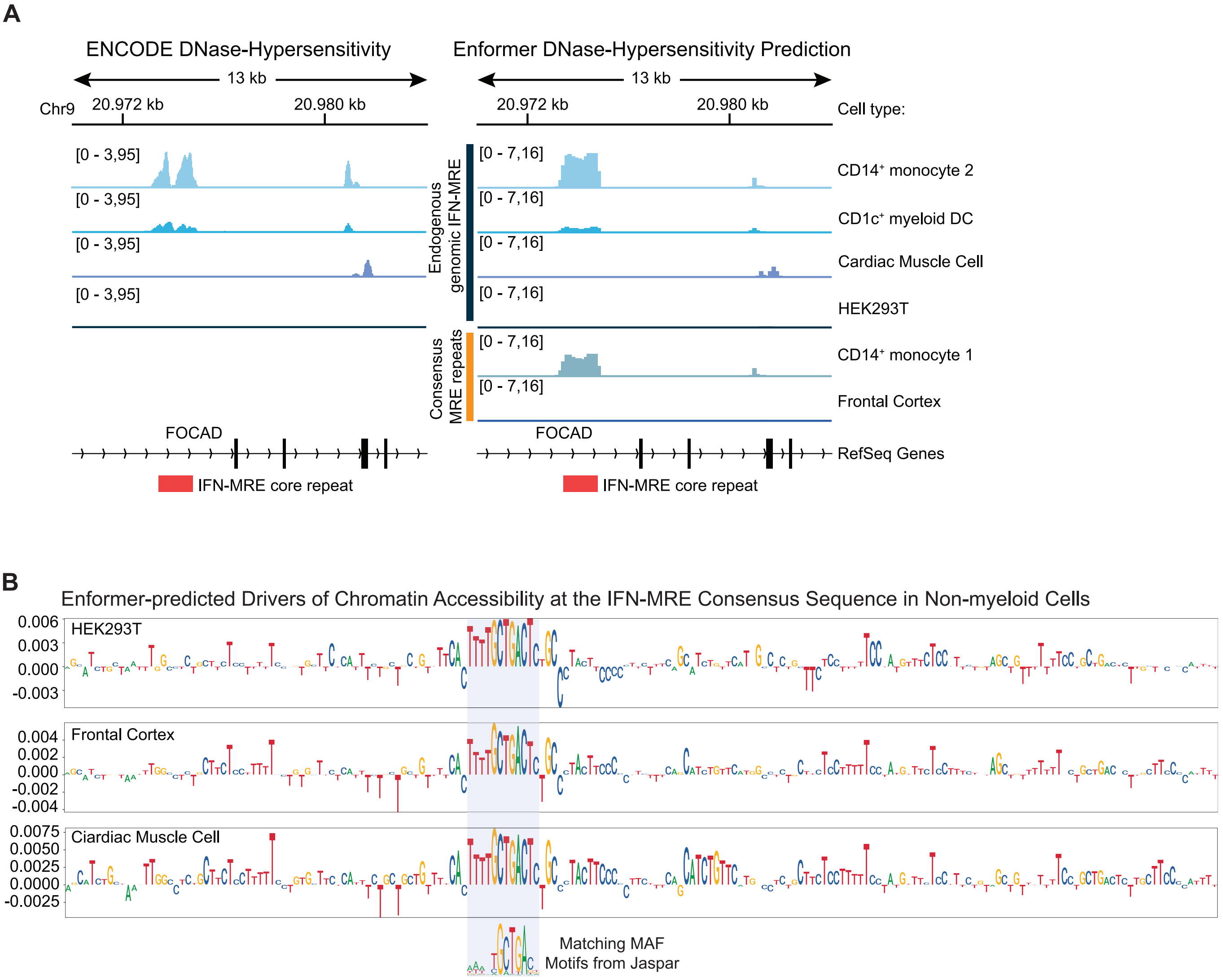

Figure EV5. The deep learning sequence-to-function model Enformer predicts cell type-specific accessibility at the MRE in monocytes.

(**A**) ENCODE DNase hypersensitivity tracks and Enformer-predicted DNase hypersensitivity tracks of indicated cell types at the human genomic MRE. Additionally, the core tandem repeat units at the human MRE locus were replaced with the 7x consensus repeat sequence, and DNase hypersensitivity in indicated cell types was predicted using Enformer. (**B**) Importance of each nucleotide of the MRE consensus sequence for the Enformer-predicted chromatin accessibility in non-myeloid cells. Spans of nucleotides with high attribution scores matching a MAF motif in the Jaspar database are highlighted in blue. Since there is no chromatin accessibility at this locus in these cells, these predictions can be considered background. Source data are available online for this figure.

© The Author(s)

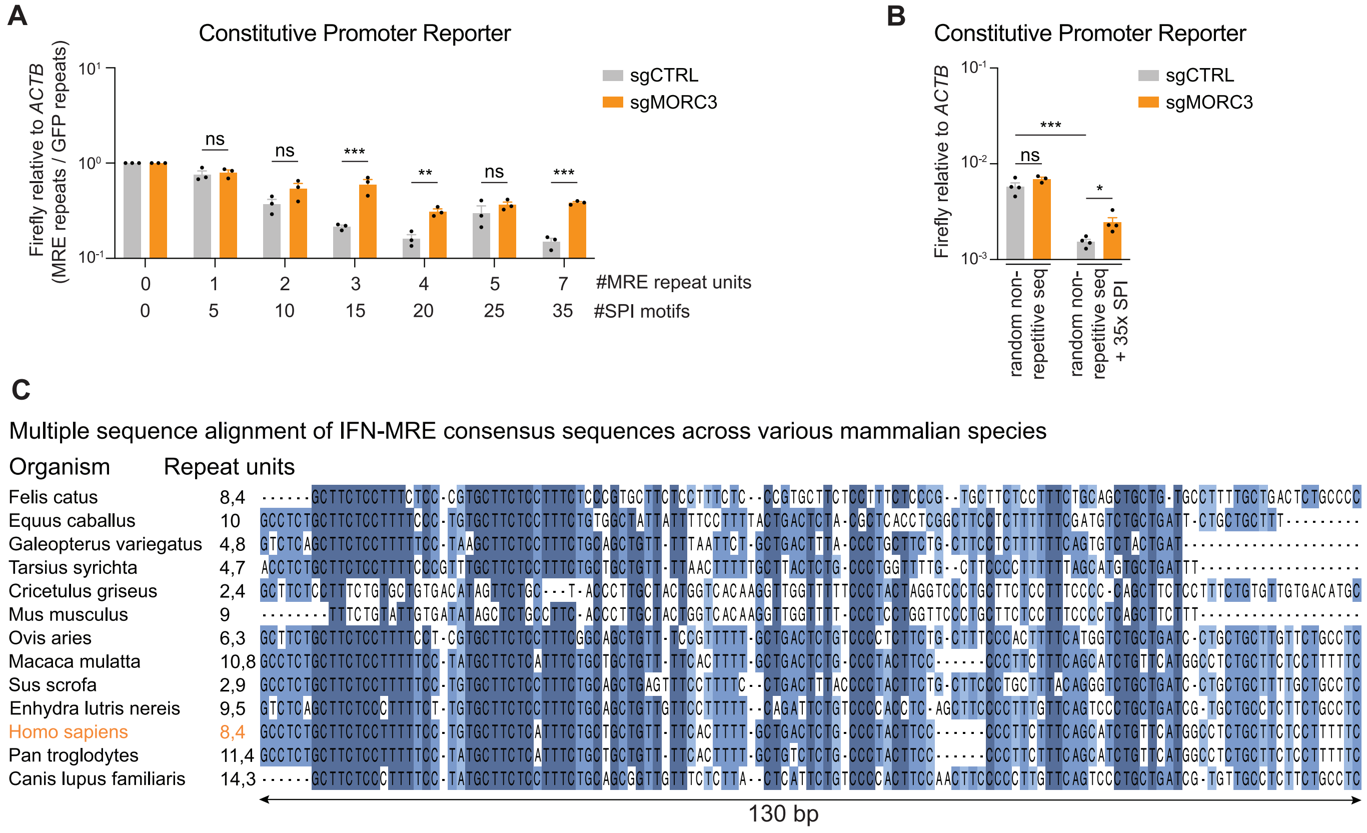

Figure EV6. MORC3 represses SPI motif clusters.

(**A**) Increasing numbers of MRE-repeat units or GFP-repeat units were inserted into the luciferase reporter and integrated into the genome of Cas9 *IFNAR1*$^{-/-}$ *IFNAR2*$^{-/-}$MRE$^{-/-}$ BLaER1 cells. Constitutive Promoter Firefly luciferase gene expression upon lentiviral sgRNA delivery is depicted. Data is normalized to GFP repeats. 0 represents the empty vector control. Data represent the mean + SEM, $n = 3$ independent experiments. 1, $P > 0.9999$; 2, $P = 0.1404$; 3, $P < 0.0001$; 4, $P = 0.0011$; 5, $P = 0.8596$; 7, $P < 0.0001$. ***$P < 0.001$; **$P < 0.01$; ns, not significantly different. (**B**) 35 SPI motifs were integrated into a random non-repetitive sequence and integrated as luciferase reporter into the genome of Cas9 *IFNAR1*$^{-/-}$ *IFNAR2*$^{-/-}$ MRE$^{-/-}$ BLaER1 cells. Constitutive Promoter Firefly luciferase gene expression upon lentiviral sgRNA delivery is shown. Data represent the mean + SEM, $n = 4$ independent experiments. ***$P < 0.0001$; *$P = 0.015$; ns, $P = 0.9562$. (**C**) Multiple-sequence alignment of consensus sequences from Mres of various species. Mres are tandem repeats enriched in Spi transcription factor binding sites in an intron of the *Focad* gene near an *Ifn* gene. Significance of differences was determined by two-way ANOVA and Bonferroni's post hoc test. Source data are available online for this figure.

 © The Author(s)

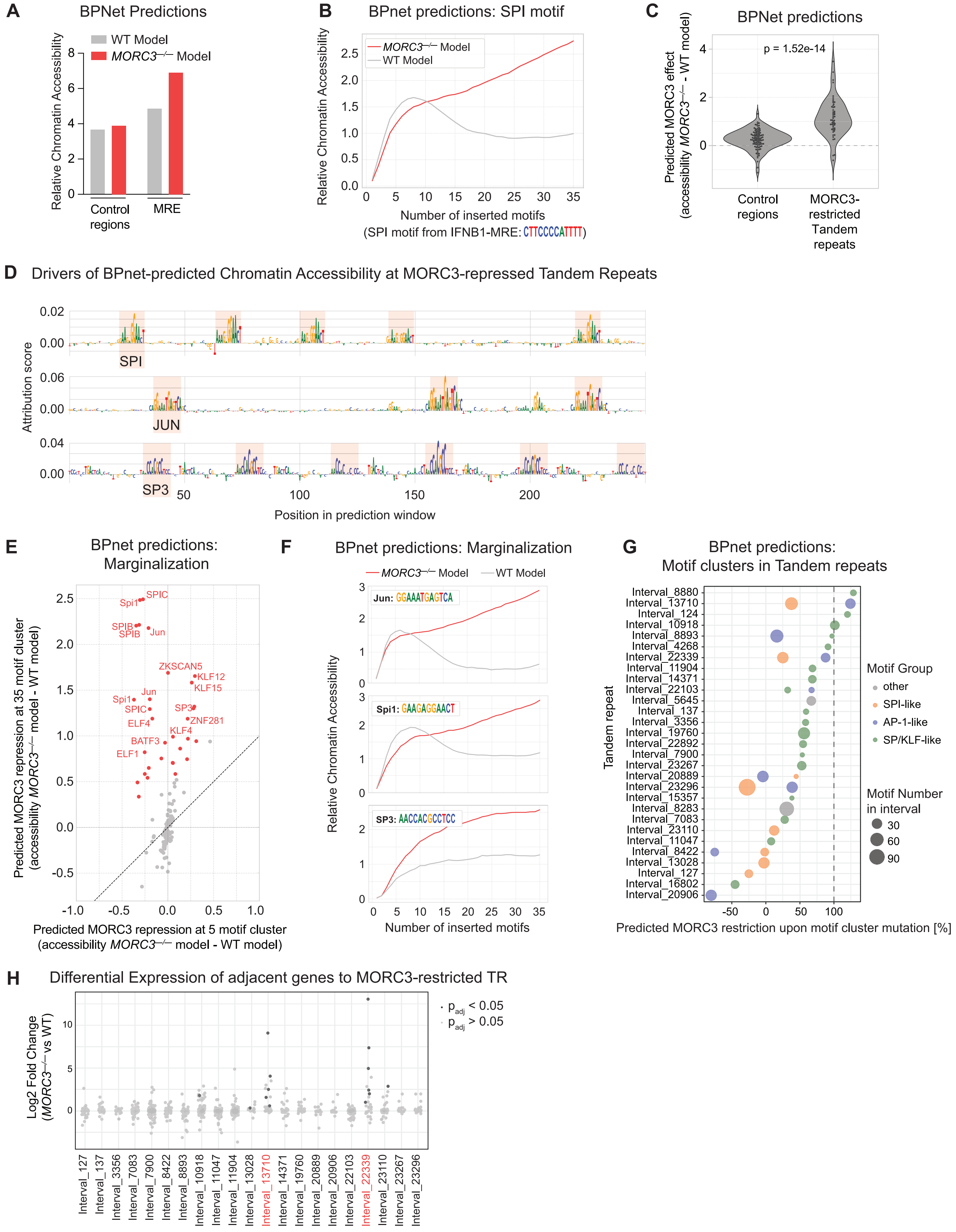

A
BPNet Predictions
WT Model
MORC3−/− Model
Relative Chromatin Accessibility
Control regions
MRE
B
BPnet predictions: SPI motif
MORC3−/− Model
WT Model
Relative Chromatin Accessibility
Number of inserted motifs
(SPI motif from IFNB1-MRE: CTTCCCCATTTT)
C
BPNet predictions
p = 1.52e-14
Predicted MORC3 effect (accessibility MORC3−/− - WT model)
Control regions
MORC3-restricted Tandem repeats
D
Drivers of BPnet-predicted Chromatin Accessibility at MORC3-repressed Tandem Repeats
Attribution score
SPI
JUN
SP3
Position in prediction window
E
BPnet predictions: Marginalization
Predicted MORC3 repression at 35 motif cluster (accessibility MORC3−/− model - WT model)
Predicted MORC3 repression at 5 motif cluster (accessibility MORC3−/− model - WT model)
F
BPnet predictions: Marginalization
MORC3−/− Model
WT Model
Jun: GGAAATGAGTCA
Spi1: GAAGAGGAACT
SP3: AACCACGCCTCC
Relative Chromatin Accessibility
Number of inserted motifs
G
BPnet predictions: Motif clusters in Tandem repeats
Tandem repeat
Motif Group
other
SPI-like
AP-1-like
SP/KLF-like
Motif Number in interval
Predicted MORC3 restriction upon motif cluster mutation [%]
H
Differential Expression of adjacent genes to MORC3-restricted TR
Log2 Fold Change (MORC3−/− vs WT)
p adj < 0.05
p adj > 0.05

Figure EV7. BP-net sequence-to-function deep learning models predict motif clusters of various transcription factors to cause MORC3-limited accessibility at tandem repeats.

(**A**) BPNet models were trained on ATAC-seq data from *IFNAR1*$^{-/-}$ *IFNAR2*$^{-/-}$ *MORC3*$^{-/-}$ or *IFNAR1*$^{-/-}$ *IFNAR2*$^{-/-}$ BLaER1 monocytes. Predicted accessibility at control regions or the MRE is shown. Data for control regions represents the average prediction value of 100 random genomic sequences. (**B**) In all, 1–35 SPI motifs were inserted into random genomic sequences, and chromatin accessibility was predicted using BPNet models that were trained on ATAC-seq data from *IFNAR1*$^{-/-}$ *IFNAR2*$^{-/-}$ and *IFNAR1*$^{-/-}$ *IFNAR2*$^{-/-}$ *MORC3*$^{-/-}$ BLaER1 monocytes. (**C**) MORC3-bound and -restricted tandem repeats were analyzed with the BPNet model trained on ATAC-seq data from *IFNAR1*$^{-/-}$ *IFNAR2*$^{-/-}$ *MORC3*$^{-/-}$ or *IFNAR1*$^{-/-}$ *IFNAR2*$^{-/-}$ BLaER1 monocytes. The MORC3 effect was calculated by subtracting the predicted accessibility of the wild-type model from the prediction of the *MORC3*$^{-/-}$ model and is plotted for 53 tandem repeats and 100 random genomic background sequences. *P* value was determined by the Wilcoxon rank-sum test. (**D**) Importance of each nucleotide for chromatin accessibility at MORC3-restricted tandem repeats predicted by the *MORC3*$^{-/-}$ BPNet model. Spans of nucleotides with high attribution scores matching SPI, JUN or SP3 motifs are highlighted in red. Representative segments of three representative tandem repeats of 23 are shown. (**E**) Motifs from clusters in (**D**) that were predicted to drive chromatin accessibility at tandem repeats in the absence of MORC3 were inserted 5 or 35 times into random sequences. Accessibility was predicted using BPNet models that were trained on ATAC-seq data from *IFNAR1*$^{-/-}$ *IFNAR2*$^{-/-}$ and *IFNAR1*$^{-/-}$ *IFNAR2*$^{-/-}$ MORC3$^{-/-}$BLaER1 monocytes. Annotations are from the Jaspar TF-motif database. (**F**) 1 to 35 SPI1, JUN, or SP3 motifs from (**D**) were inserted into random sequences, and chromatin accessibility was predicted using BPNet models that were trained on ATAC-seq data from *IFNAR1*$^{-/-}$ *IFNAR2*$^{-/-}$ and *IFNAR1*$^{-/-}$ *IFNAR2*$^{-/-}$ *MORC3*$^{-/-}$ BLaER1 monocytes. (**G**) Indicated motif clusters in tandem repeats were mutated in silico, and MORC3 restriction (accessibility in *MORC3*$^{-/-}$ - WT model) was predicted and is depicted normalized to the non-mutated WT sequence. 100% means that MORC3 still restricts this interval if the motif cluster is mutated. 0% means that MORC3 does not restrict this interval upon mutation of the motif cluster. Dot size reflects the number of motifs in the mutated cluster. (**H**) Expression of genes within 2 mb of a MORC3-restricted tandem repeat in BLaER1 monocytes comparing *IFNAR1*$^{-/-}$ *IFNAR2*$^{-/-}$, *IFNB1*$^{-/-}$, Cas9 *STAT1*$^{-/-}$ *STAT2*$^{-/-}$ and Cas9 *STAT1*$^{-/-}$ *STAT2*$^{-/-}$ IFNB1-MRE$^{-/-}$ monocytes, and matched *MORC3*$^{-/-}$ monocytes from GSE183011 ($n = 10$). *P* values were calculated as part of DESeq2 using the Wald test and adjusted for multiple testing (P_{adj}) using the Benjamini–Hochberg method. Interval_13710 is the TWIST2-MRE, Interval_22339 is the IFNB1-MRE. Source data are available online for this figure.

 © The Author(s)

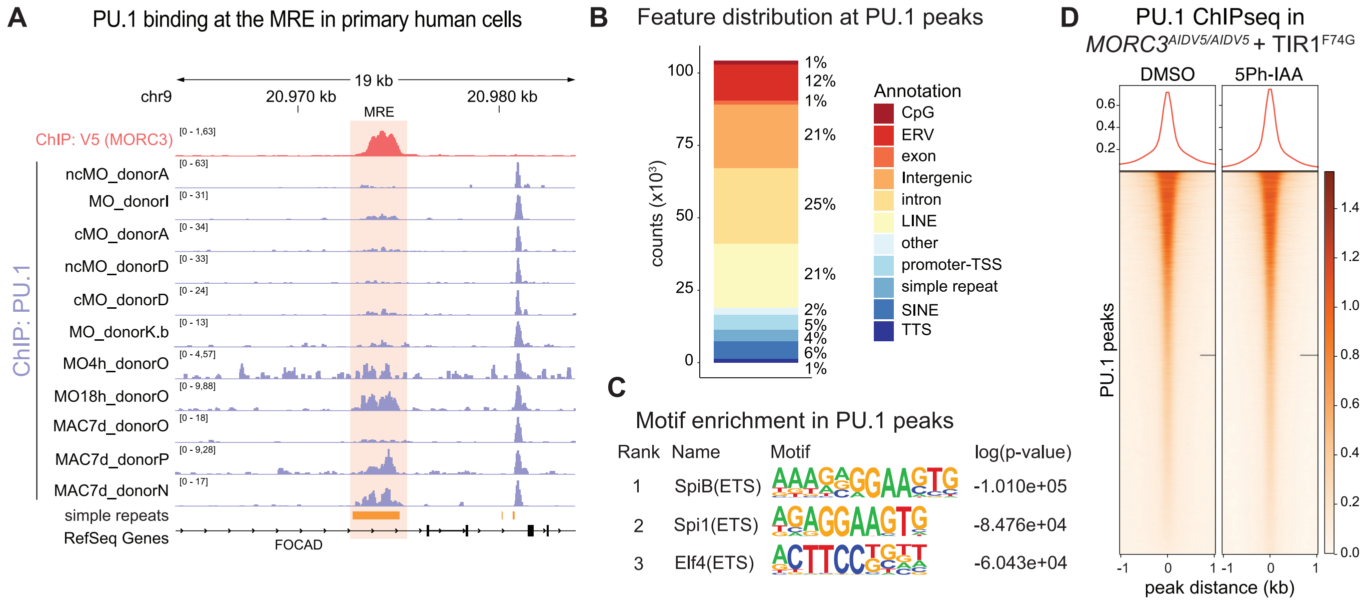

Figure EV8. PU.1 binds to the IFNB1-MRE in primary human myeloid cells.

(**A**) Genome browser view of PU.1 ChIP-seq coverage in primary human myeloid cells and MORC3-V5 ChIP-seq in BLaER1 monocytes at the human IFNB1-MRE. PU.1 data is from GSE128837. (**B**, **C**) Genomic feature annotation and top 3 enriched motifs across PU.1 ChIP-Seq peaks in Cas9 *IFNAR1*$^{-/-}$ *IFNAR2*$^{-/-}$ *MORC3*$^{AIDV5/AIDV5}$ TIR1^{F74G} BLaER1 monocytes. (**D**) Read-density heatmap showing the normalized coverage of PU.1 ChIP-seq signal centered around PU.1 peaks from *IFNAR1*$^{-/-}$ *IFNAR2*$^{-/-}$ *MORC3*$^{AIDV5/AIDV5}$ TIR1^{F74G} BLaER1 monocytes treated with DMSO or 5Ph-IAA for 24 h. Source data are available online for this figure.

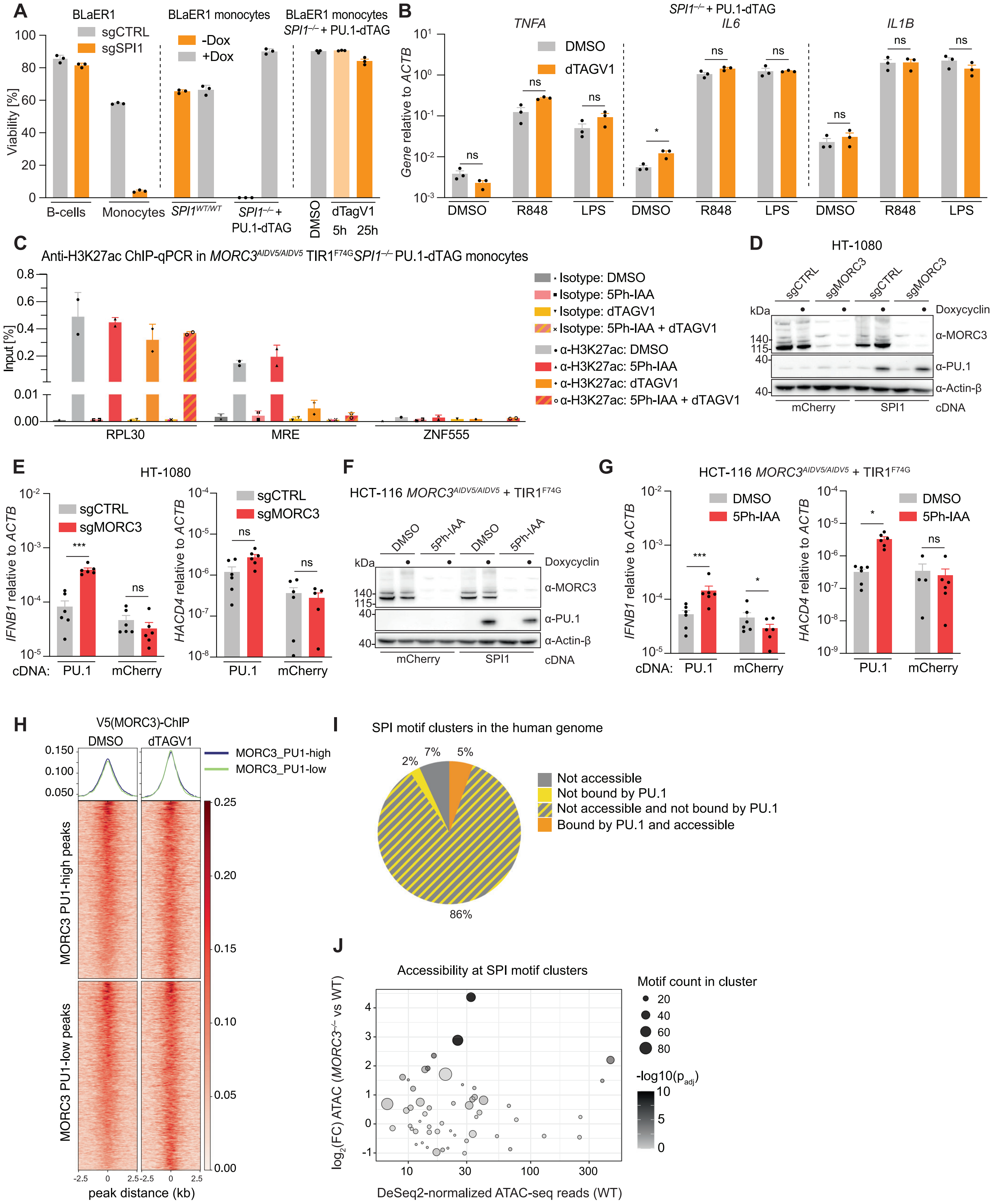
A
BLaER1
sgCTRL
sgSPI1
BLaER1 monocytes
-Dox
+Dox
BLaER1 monocytes
SPI1−/− + PU.1-dTAG
Viability [%]
B-cells
Monocytes
SPI1WT/WT
SPI1−/− + PU.1-dTAG
DMSO
dTagV1
5h 25h
B
SPI1−/− + PU.1-dTAG
TNFA
IL6
IL1B
DMSO
dTAGV1
Gene relative to ACTB
ns
DMSO
R848
LPS
C
Anti-H3K27ac ChIP-qPCR in MORC3AIDV5/AIDV5 TIR1F74G SPI1−/− PU.1-dTAG monocytes
Input [%]
Isotype: DMSO
Isotype: 5Ph-IAA
Isotype: dTAGV1
Isotype: 5Ph-IAA + dTAGV1
α-H3K27ac: DMSO
α-H3K27ac: 5Ph-IAA
α-H3K27ac: dTAGV1
α-H3K27ac: 5Ph-IAA + dTAGV1
RPL30
MRE
ZNF555
D
HT-1080
sgCTRL
sgMORC3
kDa
Doxycyclin
α-MORC3
α-PU.1
α-Actin-β
mCherry
SPI1
cDNA
E
HT-1080
IFNB1 relative to ACTB
HACD4 relative to ACTB
sgCTRL
sgMORC3
cDNA:
PU.1
mCherry
F
HCT-116 MORC3AIDV5/AIDV5 + TIR1F74G
DMSO
5Ph-IAA
G
HCT-116 MORC3AIDV5/AIDV5 + TIR1F74G
DMSO
5Ph-IAA
H
V5(MORC3)-ChIP
DMSO
dTAGV1
MORC3_PU1-high
MORC3_PU1-low
MORC3 PU1-high peaks
MORC3 PU1-low peaks
peak distance (kb)
I
SPI motif clusters in the human genome
Not accessible
Not bound by PU.1
Not accessible and not bound by PU.1
Bound by PU.1 and accessible
2%
7%
5%
86%
J
Accessibility at SPI motif clusters
Motif count in cluster
-log10(padj)
log2(FC) ATAC (MORC3−/− vs WT)
DeSeq2-normalized ATAC-seq reads (WT)

Figure EV9. Characterization of PU.1-dTAG BLaER1 monocytes and PU.1 levels are the limiting factor for IFNB1-MRE activation in other cells.

(**A**) Cell viability of Cas9 *IFNAR1*$^{-/-}$ *IFNAR2*$^{-/-}$ *MORC3*$^{AIDV5/AIDV5}$ TIR1^{F74G} BLaER1 B-cells or monocytes expressing sgCTRL or sgSPI1 (left); Cas9 *IFNAR1*$^{-/-}$ *IFNAR2*$^{-/-}$ *MORC3*$^{AIDV5/AIDV5}$ TIR1^{F74G} *SPI1*$^{WT/WT}$ or Cas9 *IFNAR1*$^{-/-}$ *IFNAR2*$^{-/-}$ *MORC3*$^{AIDV5/AIDV5}$ TIR1^{F74G} *SPI1*$^{-/-}$ PU.1-dTAG-cDNA BLaER1 monocytes stimulated with doxycycline (middle). The PU.1-dTAG rescue construct is under the control of a dox-inducible promoter. And cell viability of dox-stimulated Cas9 *IFNAR1*$^{-/-}$ *IFNAR2*$^{-/-}$ *MORC3*$^{AIDV5/AIDV5}$ TIR1^{F74G} *SPI1*$^{-/-}$ PU.1-dTAG-cDNA BLaER1 monocytes stimulated with dTAGV1 for indicated time (right). Data is shown as mean + SEM of $n = 3$ independent experiments. (**B**) Gene expression analysis of dox-stimulated Cas9 *IFNAR1*$^{-/-}$ *IFNAR2*$^{-/-}$ *MORC3*$^{AIDV5/AIDV5}$ TIR1^{F74G} *SPI1*$^{-/-}$ PU.1-dTAG-cDNA BLaER1 monocytes stimulated with dTAGV1 for 24 h and with the indicated TLR-ligands for 4 h. Data are shown as mean + SEM of $n = 3$ independent experiments. *TNFA*: DMSO, $P = 0.3794$; R848, $P = 0.0767$; LPS, $P = 0.2088$; *IL6*: DMSO, $P = 0.0224$; R848, $P = 0.4854$; LPS, $P > 0.9999$; *IL1B*: DMSO, $P > 0.9999$; R848, $P > 0.9999$; LPS, $P = 0.9586$ as determined by two-way ANOVA and Bonferroni's post hoc test. *$P < 0.05$; ns, not significantly different. (**C**) ChIP-qPCR analysis of H3K27ac in dox-stimulated Cas9 *IFNAR1*$^{-/-}$ *IFNAR2*$^{-/-}$ *MORC3*$^{AIDV5/AIDV5}$ TIR1^{F74G} *SPI1*$^{-/-}$ PU.1-dTAG BLaER1 monocytes from $n = 2$ independent experiments. If indicated, cells were pretreated with dTAGV1 for 1 h before stimulation with 5Ph-IAA or DMSO for 24 h. The RPL30 promoter serves as a positive control for H3K27ac, the ZNF555 locus as a negative control. Missing datapoints equal non-detected values in qPCR. (**D–G**) Cas9 HT-1080 fibrosarcoma and *MORC3*$^{AIDV5/AIDV5}$ TIR1^{F74G} HCT-116 colorectal carcinoma cell lines were transduced with dox-inducible cDNAs encoding PU.1 or mCherry, and MORC3 was deleted with lentivirus expressing anti-MORC3 sgRNAs or degraded with 5Ph-IAA. Gene expression of IFNB1-MRE-regulated genes *IFNB1* and *HACD4* from $n = 6$ independent experiments or one representative immunoblot of two upon treatment with doxycycline for 48 h. HT-1080: *IFNB1*: PU.1, $P = 0.0001$; mCherry, $P = 0.3647$. *HACD4*: PU.1, $P = 0.3217$; mCherry, $P > 0.9999$. HCT-116: *IFNB1*: PU.1, $P = 0.0002$; mCherry, $P = 0.0414$. *HACD4*: PU.1, $P = 0.0105$; mCherry, $P > 0.9999$ as determined by two-way ANOVA and Bonferroni's post hoc test. ***$P < 0.001$; *$P < 0.05$; ns, not significantly different. Missing datapoints equal non-detected values in qPCR. (**H**) Read-density heatmap showing the normalized coverage of anti-V5 (MORC3) ChIP-seq and centered around MORC3-V5 peaks in dox-stimulated Cas9 *IFNAR1*$^{-/-}$ *IFNAR2*$^{-/-}$ *MORC3*$^{AIDV5/AIDV5}$ TIR1^{F74G} *SPI1*$^{-/-}$ PU.1-dTAG BLaER1 monocytes treated with DMSO or dTAGV1 for 24 h. MORC3-V5 peaks were grouped by high and low PU.1 signal. (**I**) 992 SPI motif clusters with >15 binding sites in the human genome were grouped according to chromatin accessibility (ATAC-seq) and PU.1 ChIP-seq signal. (**J**) Chromatin accessibility determined by ATAC-seq at accessible and PU.1-bound SPI-motif clusters comparing *IFNAR1*$^{-/-}$ *IFNAR2*$^{-/-}$ and *IFNAR1*$^{-/-}$ *IFNAR2*$^{-/-}$ *MORC3*$^{-/-}$ BLaER1 monocytes ($n = 3$ independent experiments). Data is from GSE183011. P values were calculated as part of DESeq2 using the Wald test and adjusted for multiple testing (P_{adj}) using the Benjamini–Hochberg method. Source data are available online for this figure.

© The Author(s)

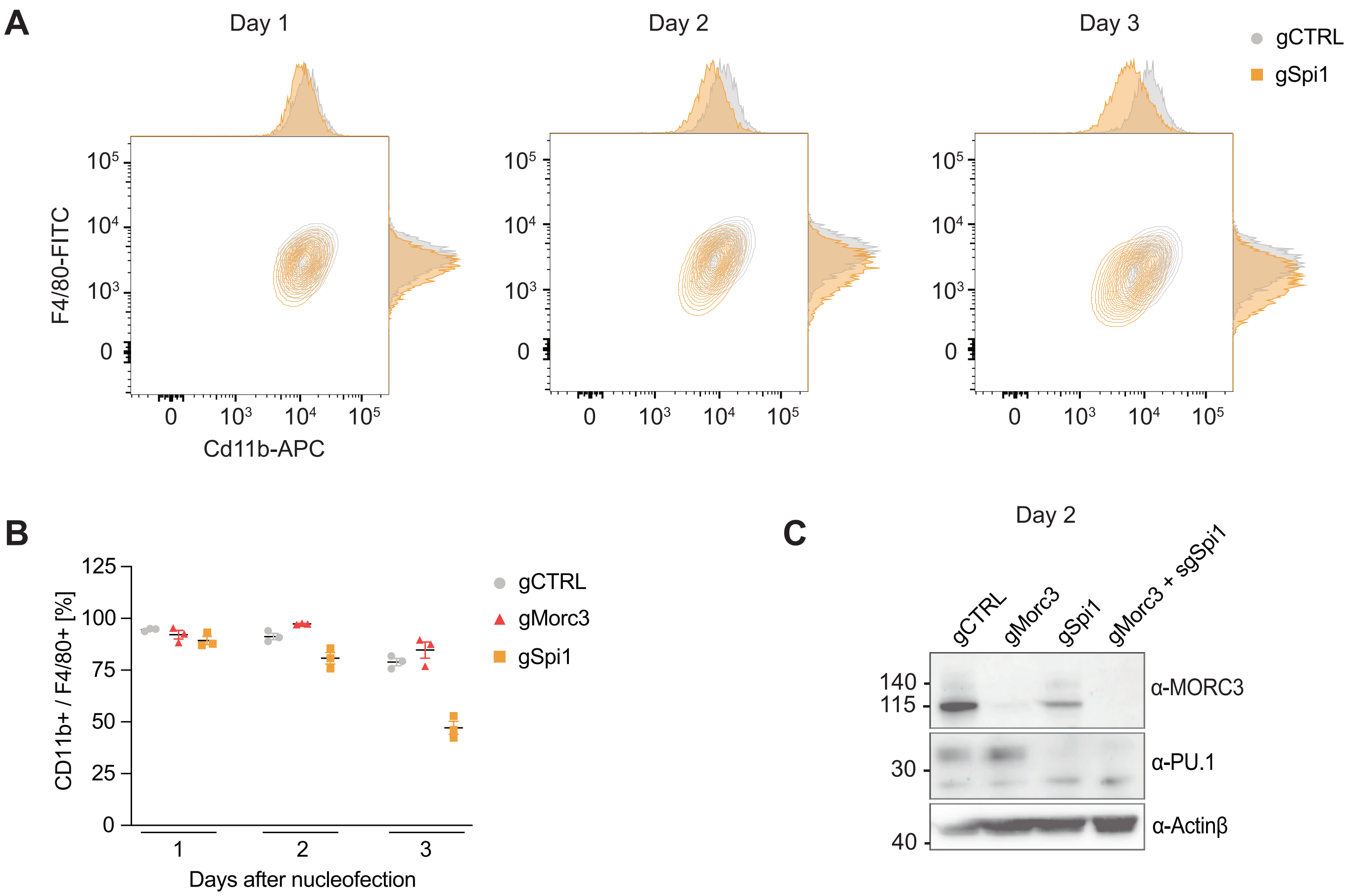

Figure EV10. Genetic deletion of the essential factor PU.1 in BMMs.

(**A**, **B**) Cd11b and F4/80 FACS-analysis of *Ifnar1*$^{-/-}$ BMMs at day 1, 2, or 3 after nucleofection with indicated Cas9:RNPs depicted as one representative experiment (**A**) and mean ± SEM of $n = 3$ independent experiments. (**C**) Immunoblot analysis of *Ifnar1*$^{-/-}$ BMMs at day 2 after nucleofection with indicated Cas9:RNPs showing one representative immunoblot of two. Source data are available online for this figure.

 © The Author(s)
