# Supplementary material for: MORC3 represses a tandem repeat enhancer to regulate interferon
Source: EMBO J. 2026 Jun 5;45(14):5130–63. doi: 10.1038/s44318-026-00799-9 (PMC13373173; doi:10.1038/s44318-026-00799-9)
Supplement: Supplementary file 1 — Peer Review File [file 44318_2026_799_MOESM1_ESM.pdf]

THE EMBO JOURNAL

MORC3 represses a tandem repeat enhancer to regulate interferon

Luisa Krumwiede, David Hollaus, Erika Valeri, Karina Schindler-Schumitsch, Monika Bazyl, Johanna Schiedlbauer, Nanette Becht, Markus Jaritz, Bernardo de Almeida, Siegfried Schloissnig, Dara Burdette, Jacob Schreiber, Alexander Stark, and Moritz Gaidt

Corresponding author(s): Moritz Gaidt (moritz.gaidt@imp.ac.at)

Review Timeline:

Submission Date:	12th Jul 25
Editorial Decision:	9th Sep 25
Revision Received:	26th Dec 25
Editorial Decision:	26th Feb 26
Revision Received:	13th Mar 26
Accepted:	7th Apr 26

Editor: Cornelius Schneider

Transaction Report:

(Note: With the exception of the correction of typographical or spelling errors that could be a source of ambiguity, letters and reports are not edited. Depending on transfer agreements, referee reports obtained elsewhere may or may not be included in this compilation. Referee reports are anonymous unless the Referee chooses to sign their reports.)

Dear Dr. Gaidt,

Thank you for submitting your manuscript for consideration by the EMBO Journal. It has now been seen by three referees whose comments are shown below.

As you can see from the reports, the referees think that the findings are potentially interesting but voice multiple concerns which on one hand question the physiological significance of the findings and also include requests which would require multiple experiments to provide more mechanistic insight.

Given these overall critical opinions and the fact that the EMBO Journal can only afford to accept papers which receive enthusiastic support from a majority of referees, I am afraid we can not offer to publish it here.

While we cannot continue with this manuscript at our journal, we have discussed the study with our colleagues at EMBO reports. The editorial team at EMBO reports is interested in these findings, and would like to publish this manuscript at EMBO Reports pending the following revisions: - key findings need to be verified/tested in a more physiologically relevant cell line (ref #1, ref #3) - ATAC-seq data should be complemented by RNA-seq (ref #2, #3) - The specificity or generalizability of the proposed repression of tandem repeat enhancers by MORC3 and the regulation of IFN-b by PU.1 should be further investigated (ref #1, Fig 4D, ref #2, major comment 1, ref #3, major issues). - All other concerns from the referees should be addressed, but it will not be required to provide and in-depth mechanistic understanding (ref #1, major comment 2). If you are interested in this option, please submit the manuscript to EMBO Reports via the transfer link below. Please feel free to contact the handling editor Esther Schnapp at e.schnapp@emboreports.org if you have further questions regarding the transfer and revision progress. Please note that no reformatting of the manuscript is necessary for the transfer.

Thank you in any case for the opportunity to consider this manuscript. I am sorry we cannot be more positive on this occasion, but we hope nevertheless that you will find our referees' comments helpful.

Yours sincerely,

Cornelius Schneider

Cornelius Schneider, PhD
Editor
The EMBO Journal
c.schneider@embojournal.org

****

Referee #1:

The manuscript by Krumwiede et al. describes new research findings in the field of antiviral immunity. The authors investigated how the human immune restriction factor MORC3 suppresses the expression of type 1 IFN by binding to a distant regulatory cis-element. They found that this element, referred to as MRE, consists of repetitive DNA sequences that contain a unique arrangement of multiple consensus binding sites for SPI transcription factors. They further report that these sites are bound by PU.1, an SPI transcription factor highly expressed in monocytes, and that PU.1 recruits MORC3 to form a repressor complex at the MRE.
Overall, this is an interesting study on an important topic. However, in my opinion, the study in its current form has some shortcomings that dampen my enthusiasm. The strongest criticism is that the conclusions are drawn almost entirely from analyses in a single cell line in vitro. Moreover, this cell line (BLaER1) is somewhat artificial, as it originates from a B-leukemia line (with an oncogenic Myc fusion) that was reprogrammed into a monocyte-like phenotype through ectopic C/EBP expression. As such, these cells are (epigenetically) distant from naturally developed monocytes. Therefore, in my opinion, it remains unclear to what extent the reported results actually reflect a physiological situation or physiological mechanism. The results would be more meaningful if the authors could verify their findings (at least the most important ones) in normal (non-cancerous) monocytes, preferably also in vivo.
Furthermore:
Fig. 1: What was used as background control cells in ChIP-seq experiments? Optimally, BLaER1 cells treated with 5Ph-IAA carrying the empty degron construct (= without MORC3 cDNA) would be used.

Fig. 2: Why were IFNAR1/2-/- cells used? Moreover, I assume that "sgMORC3" means that the authors deleted MORC3 using CRISPR/Cas? If so, they should provide Western blotting data to confirm the absence of the MORC3 protein. Furthermore, were the constructs used in Fig. 2E (and those used in similar experiments, e.g., in Fig. 2D) sequence verified?
Fig. S2C and related: Did the authors normalize the copy numbers of the genome-integrated luciferase construct in each experiment using qPCR? Would it not be much easier to insert a genomic isolator sequence between the luciferase cassettes to protect the firefly luciferase from the MRE effect?
Fig. 2D: Deleting 35 SPI sites may generate a sequence forming an aberrant spatial structure that is different from the original, non-mutated sequence stretch. It would be better to replace base pairs with ones that specifically abolish ETS/SPI1 binding (as for example doi.org/10.1074/jbc.274.8.4939).
Fig. 4A: Different numbers of complete MRE repeat units were used here. How can the authors conclude from this experiment that the number of SPI1 motifs, but not other sequences in the MRE repeats, determine the specified enhancer/repressor activities?
Fig. 4B: I cannot see any repressive effect mediated by the SPI1 motifs (compare the two gray bars). Therefore, I cannot agree with the authors' conclusion that "large SPI motif clusters are sufficient for MORC3-mediated repression" (lines 216/7).
Fig. 4D: Does the number of MRE repeats and/or the number of SPI motifs within the MRE correlate with differences in IFNB1 expression in the human population?
Fig. 5/Chapter titled "MORC3 suppressed motif clusters of multiple transcription factors." This chapter contains, as I can see, no experimentally verified functional data. All conclusions are based solely on in silico predictions and correlative data. Therefore, the statements are far too strong. The authors should provide experimental validation or tone down their conclusions.
Fig. 6: The authors should measure the expression status of MORC3 in PU.1-downregulated cells. In addition, they should also check publicly available ChIP-seq data for PU.1 binding to the MRE in normal macrophages/monocytes.
Minor:
The authors claim that MORC3 tagging resulted in "mild" hypomorphic activity, leading to "slight" derepression of IFN in the absence of the induction compound. However, as I see in Fig. S1A, this derepression is >10-fold. I do not believe this is "minor" and as such suggest (although it is not critical to the overall conclusions) that the authors describe this effect more realistically.

Referee #2:

In "MORC3 represses tandem repeat enhancers to regulate interferon", Krumwiede et al. investigated the mechanism for the MORC3-mediated de-repression of Ifnb1 gene transcription. The authors systematically defined the "IFNB1-MRE" as a region within an intron of the gene Focad that is characterized by several tandem repeats containing dozens of interspersed SPI motifs. The SPI motifs are required and sufficient to recruit MORC3 to the IFNB1-MRE. Through the clever use of degrons linked to MORC3 and the transcription factor PU.1 (knockins and overexpression, respectively), the authors demonstrated that binding of MORC3 to the IFNB1-MRE represses Ifnb1 transcription by presumably preventing accessibility of SPI motifs to PU.1. While the mechanism of repression of IFNB1-MRE by MORC3 is generally well-defined by the authors, many important questions remain, especially with respect to the generality of the MORC3-mediated de-repression of gene expression.

Major Comment 1. The authors make two interconnected claims that are inadequately substantiated by the present data:
(1) "motif clusters are sufficient for MORC3-mediated repression and enhancer activity upon MORC3 loss" (line 26 and others).
(2) a large set of genes are negatively regulated (i.e., repressed) by MORC3 via tandem repeats (lines 104-106 and others "86% of the strongly MORC3-repressed loci...were tandem repeats").
Both of these claims presume that MORC3 broadly represses gene transcription, and MORC3 repression occurs at bound sites containing tandem repeats. However, according to their own gene expression analysis and a previous report in IFN signaling-null cells (Gaidt et al., 2021), very few genes display transcriptional changes upon loss of MORC3 (Fig. S1C), even though MORC3 is found bound to DNA at several locations in the genome (Line 91, >6000 MORC3 ChIP-seq peaks for MORC3). Furthermore, while the authors assert that there are 7 strongly MORC3-repressed loci and 147 total repressed sites (lines 104-105), the referenced data to corroborate that statement is ATACseq (Fig. 1E, F). ATACseq measures chromatin openness, not direct transcriptional activities per se. Since MORC3 is found at promoters with active transcription (i.e., H3K4me3, Li et al., 2016) (Fig. 1D), MORC3 binding to DNA does not automatically mean a repressive function (which is also clear through their transcriptional analysis). The authors concede that MORC3 binding to DNA, in general, or specifically to tandem repeats does not automatically equate to transcriptional repression (as briefly commented on in lines 101-102 and lines 276-278). Yet throughout this study, MORC3 is framed to have a repressive mechanism that is much more substantial/universal than is believable with the current data as presented.
To address the question of actual MORC3 repressive abilities in the context of tandem repeats (beyond the repression of Ifnb1, which is not a novel finding), the authors examined hypothetical/synthetic in silico gene repression by MORC3 (Fig. 5A). However, this data is not convincing. The only in cellulo data provided involves somewhat weak transcriptional changes upon loss of MORC3 for two "strongly MORC3-repressed loci" (Fig. S5C). To help generalize the MORC3 repressive capabilities experimentally, the authors should more deeply examine the evolutionary implications. For example, the authors could use their Minimal Luc reporter with the MRE and MORC3s from different species. A murine monocyte model could also be explored. In addition, the authors could examine sufficiency by overexpressing PU.1 in other MORC3-deficient cell types that do not otherwise have MORC3-dependent repression of Ifnb1 (see line 28-287).
Major Comment 2. Mode of MORC3-repression of PU.1-mediated Ifnb1 transcription is not explained. MORC3 repression of the IFNB1-MRE could occur in a variety of ways to ultimately reduce accessibility of PU.1 to chromatin. What is happening to the

IFNB1-MRE by MORC3 that prevents PU.1 accessibility? How can PU.1 be involved in establishing H3K27ac histone marks at the IFNB1-MRE in the presence of MORC3 if MORC3 also limits PU.1 accessibility? These questions need to be answered in order to move this study beyond its current descriptive nature.

Minor Comments.
1. Line 57. "MORC3...inhibits the production of the type I interferon IFN-b" is slightly misleading as MORC3 actually inhibits an enhancer element that can drive Ifnb1 transcription independently of PRR activation. The statement, as is, implies that any pathway to Ifnb1 or IFN-b signaling, itself, is repressed by MORC3.
2. Line 102. Based on the presented data, MORC3 is not required for repression (the authors say sufficiency here).
3. Line 330-338. For clarity, I suggest revising to "...MORC3-mediated IFN repression in two ways. First, MORC3 repression requires... ...Second, it is likely that not all TFs..."
4. Fig 2A. The Human Chr9 schematic could be reworked to be more clear. For example, nucleotide distance to Ifnb1 should be noted.
5. Multiple figures (3D, 3E, 6C, 6D, S6F, etc.) have an "ND" or "not detected" notation on the graph over top of sometimes quite large bars. Are these really values that are not detected? If they are truly not detected, then it would be clearer to have no bars shown on the graph and just ND over a blank spot (and the limit of detection can be noted somewhere else, if need be).

Referee #3:

In this manuscript, the authors explore the mechanisms through which MORC3 regulates IFNB transcription through negative repression. The senior author has previously described a so-called MORC3-regulated element (MRE) that is situated adjacent to the IFNB locus, and that upon inhibition of MORC3 (by viral virulence factors such as ICP0), IFNB transcription is rapidly upregulated. Interestingly, this transcription is independent of canonical IFN-inducing transcription factors (IRF3/7), while MORC3 is dispensable for canonical IFNB-inducing stimuli. This manuscript addresses the question of how MORC3 preferentially regulates the IFNB locus, and how this cis-regulatory element promotes such robust IFNB induction upon loss of MORC3.
The authors begin by characterizing MORC3 binding patterns by performing ChIP-seq. Interestingly, while MORC3 was found to bind to many sites (including H3K4me3 negative sites), loss of MORC3 did not lead to broad de-repression of genes (Fig. S1C), suggesting that binding of MORC3 is not sufficient for repression. ATAC-seq analysis comparison between WT and MORC3-/- BLaER1 monocytes demonstrated that sites of active repression by MORC3 were strongly enriched for tandem repeats. One particular tandem repeat site was identified within the previously defined MRE, and deletion of these tandem repeats ameliorated IFNB induction upon loss of MORC3, suggesting their requirement for MORC3-mediated suppression. Subsequently, using motif analysis, the authors determine that these tandem repeats consist of numerous SPI transcription factor binding motifs, which are required for both repression (MORC3-sufficient) and enhancer activity (upon loss of MORC3). It is important to note that the number of SPI motifs positively correlates with repression. PU.1, an SPI member with high expression in monocytes, was hypothesized to be the TF responsible for MORC3-mediated IFNB transcriptional control. Indeed, inducible deletion of PU.1 extinguished IFNB induction upon loss of MORC3, while having no effect on IFNB induction after STING activation, confirming its specific role in the MORC3-mediated pathway. Additionally, deletion of PU.1 decreased MORC3 binding to the MRE (via ChIP-qPCR), suggesting a mechanism wherein PU.1 binding to the MRE facilitates recruitment of MORC3 for negative regulation of IFNB at steady state, and loss of MORC3 enables PU.1-mediated transcription of IFNB.
In summation, this manuscript defines the mechanisms through which the previously described MRE facilitates rapid IFNB induction upon loss of MORC3. The authors nicely demonstrate, using a variety of molecular tools, the enrichment of SPI motif-containing tandem repeats at the IFNB-MRE that is critical for MORC3-mediated repression and that PU.1 is a critical TF for both repression and enhancement upon loss of MORC3. The study however has important gaps that should be addressed.

Major issues:
The biggens concern I have is that experiments are primarily done using the human BLaER1 monocytes and in order to make sure that these are not cell line specific findings, it is critical for the authors to knock down MORC3 in primary human monocytes and examine induction of IFNb and ISGs. Additionally, it is critical for the authors to examine other inflammatory cytokines that are not type I IFNs especially in the context of PU.1 knockdown. What is the status of Nf-kB dependent genes such as TNF, IL-1b and IL-6 when PU.1 is not present and also how do these cells respond to STING agonists and TLR agonists. In the absence of this specificity, it would be hard to conclude that in the absence of MORC3, there is specific induction of IFNb by PU.1.
Is the IFNB-MRE the only MORC3-bound site with Tandem Repeats/SPI motifs?
What is required for MORC3-binding to genomic regions? Is it Tandem repeats, is it H3K4me3?
The authors state that "MORC3 represses tandem repeats enriched with ETS, AP-1, or SP/KLF motifs, likely due to the high density of these motifs" How does this fit with lack of broad gene upregulation seen in Fig. S1C (i.e., shouldn't loss of MORC3 lead to upregulation of additional genes containing these motifs?

Minor Issues:
- MORC3 binds to non-Tandem repeat promoter regions affecting chromatin accessibility, but loss of MORC3 does not lead to de-repression of adjacent genes. In these settings, what is MORC3 doing upon binding if not repressing? (Fig. S1C-D).
- Authors state "MORC3-mediated silencing can spread to adjacent active genetic elements" (Fig. 2F) but there is no evidence of this in their initial analysis of MORC3 bound promoters (Fig. S1C).

- Fig. 3D/E shows that the 35 SPI motifs are critical for MORC3 mediated suppression but would have been nice to show this IFNB transcriptional levels as opposed to luciferase activity (if possible to do?) Same with Fig. 4A
- Are SPI transcription factors bound to IFNB-MRE at steady state via ChIP seq?
o CoIP experiments would be good to examine MORC3 binding to PU.1
- Important to demonstrate that loss of SPI motifs did not affect canonical IFNB-inducing stimuli (IRF3/7 dependent pathway)
- Authors state no allele in human data set had less than 7 repeats and reference Fig 4A/S4A but that is not that data)

** As a service to authors, EMBO Press provides authors with the possibility to transfer a manuscript that one journal cannot offer to publish to another EMBO publication or the open access journal Life Science Alliance launched in partnership between EMBO Press, Rockefeller University Press and Cold Spring Harbor Laboratory Press. The full manuscript and if applicable, reviewers' reports, are automatically sent to the receiving journal to allow for fast handling and a prompt decision on your manuscript. For more details of this service, and to transfer your manuscript please click on Link Not Available. **

Please do not share this URL as it will give anyone who clicks it access to your account.

We thank the reviewers and the editor for their constructive comments. Addressing their comments has substantially improved the manuscript. Our point-by-point responses are numbered and indicated in blue.

These are the major new experiments and conclusions:

1. We validated our central findings in primary cells by confirming that MORC3 antagonizes PU.1 binding at the IFNB1-MRE and that PU.1 drives *IFNB1* induction specifically upon loss of MORC3 in mouse macrophages (Fig. 7).
2. We identified the molecular mechanism underlying MORC3-mediated silencing at the IFNB1-MRE. MORC3 limits chromatin accessibility by recruiting the histone chaperone DAXX and enabling H3.3 incorporation (Fig. 3).

Minor new experiments/conclusions:

1. Because repetitive elements such as tandem repeats pose challenges for short-read sequencing analyses, we validated our computational mapping strategy using the state-of-the-art approach danbing-tk specifically designed to assign short reads to tandem repeats (Fig. S2).
2. To explain why not all motif clusters function as MORC3-repressed enhancers, we focused on SPI motifs and identified two limiting factors. First, ~95% of PU.1 motif clusters are inaccessible and unbound by PU.1 even in the absence of MORC3 (Fig. S9I). Second, ectopic PU.1 expression is sufficient to activate IFNB1-MRE enhancer activity in PU.1^{low} cells (Fig. S8D-G). Together, these results indicate that redundant chromatin silencing pathways and transcription factor availability can limit gene regulatory activities of motif clusters, although additional determinants likely contribute.

Further minor experimental additions, controls, and textual revisions have been incorporated. All changes are highlighted in yellow in the manuscript and tabulated at the end of the P2P.

Referee #1:

The manuscript by Krumwiede et al. describes new research findings in the field of antiviral immunity. The authors investigated how the human immune restriction factor MORC3 suppresses the expression of type 1 IFN by binding to a distant regulatory cis-element. They found that this element, referred to as MRE, consists of repetitive DNA sequences that contain a unique arrangement of multiple consensus binding sites for SPI transcription factors. They further report that these sites are bound by PU.1, an SPI transcription factor highly expressed in monocytes, and that PU.1 recruits MORC3 to form a repressor complex at the MRE.
Overall, this is an interesting study on an important topic. However, in my opinion, the study in its current form has some shortcomings that dampen my enthusiasm. The strongest criticism is that the conclusions are drawn almost entirely from analyses in a single cell line in vitro. Moreover, this cell line (BLaER1) is somewhat artificial, as it originates from a B-leukemia line (with an oncogenic Myc fusion) that was reprogrammed into a monocyte-like phenotype through ectopic C/EBP expression. As

such, these cells are (epigenetically) distant from naturally developed monocytes. Therefore, in my opinion, it remains unclear to what extent the reported results actually reflect a physiological situation or physiological mechanism. The results would be more meaningful if the authors could verify their findings (at least the most important ones) in normal (non-cancerous) monocytes, preferably also in vivo.

R1: We fully agree with the reviewer that validation in primary cells is essential. We have now performed a series of experiments in primary mouse bone-marrow–derived macrophages (BMMs), which confirm the central conclusions of our study (new data in Fig. 7). To avoid secondary effects of autocrine Ifn signaling after Morc3 deletion, all experiments were carried out in *Ifnar1*$^{-/-}$ BMMs.

First, CRISPR-Cas9 RNP-mediated deletion of *Morc3* led to robust induction of *Ifnb1* and another gene adjacent to the mouse IFNB1-MRE (*Hacd4*), demonstrating that the MORC3–MRE regulatory axis is conserved in primary macrophages (Fig. 7A). Second, while ChIP–qPCR analysis only detected weak PU.1 binding to the endogenous mouse IFNB1-MRE at steady state, loss of Morc3 robustly increased PU.1 occupancy, consistent with MORC3 limiting PU.1 binding to the IFNB1-MRE (Fig. 7B).

Finally, to assess the functional requirement for PU.1, we used Cas9-RNP editing to delete *Spi1* (encoding PU.1) in BMMs. Because PU.1 is essential for macrophage identity, prolonged depletion (3 days) resulted in loss of myeloid features (Fig. S10A, B). However, analysis at day 2 (when PU.1 protein was efficiently depleted while macrophage identity remained largly intact; Fig. S10B, C) allowed us to perform a clean loss-of-function experiment. These data show that PU.1 is required for *Ifnb1* induction upon loss of MORC3. Likewise, the induction of MRE adjacent gene *Hacd4* upon MORC3 depletion depended on PU.1 (Fig. 7C, D). Importantly, PU.1 deletion did not impair macrophage responses to the STING agonist diABZI (Fig. 7C), confirming that PU.1 is specifically involved in IFN responses only upon loss of MORC3.

In summary, we have validated all major findings from the BLaER1 model in primary macrophages, confirming the physiological relevance of the MORC3–PU.1–MRE regulatory mechanism.

Furthermore:
Fig. 1: What was used as background control cells in ChIP-seq experiments? Optimally, BLaER1 cells treated with 5Ph-IAA carrying the empty degron construct (= without MORC3 cDNA) would be used.

R2: Input DNA was used as background control. This is commonly done in ChIPseq experiments. In response to the reviewer's question, we have now performed anti-V5 ChIPseq in 5Ph-IAA treated *MORC3*$^{AIDV5/AIDV5}$ cells, in which MORC3 is degraded = no-epitope control. This data shows loss of signal at all MORC3 peaks, confirming the degron approach and the specificity of the ChIP (Fig. R1).

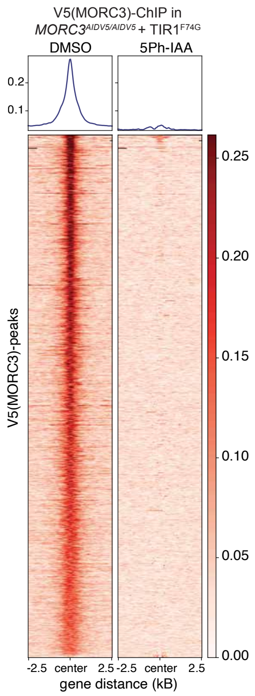

Figure R1: Specificity of anti-V5 (MORC3) ChIP
Read-density heatmap showing the normalized coverage of anti-V5 (MORC3) ChIP-seq signal centered around MORC3-V5 peaks from IFNAR1$^{-/-}$ IFNAR2$^{-/-}$ MORC3$^{AIDV5/AIDV5}$ TIR1^{F74G} BLaER1 monocytes that were treated with DMSO or 5Ph-IAA to degrade MORC3.

Fig. 2: Why were IFNAR1/2-/- cells used?

R3: Experiments were performed in BLaER1 IFNAR1/2 knockout or STAT1/2 knockout cells to prevent autocrine interferon-stimulated gene (ISG) induction following loss of MORC3. Similarly, experiments in primary bone-marrow–derived macrophages were conducted in *Ifnar1*$^{-/-}$ cells.

Deletion of both STAT1 and STAT2 was required because loss of STAT1 alone was insufficient to fully abrogate IFN-mediated ISG induction, likely due to compensatory signaling via STAT2 homodimers. IFNAR1 and IFNAR2 were both deleted in BLaER1 cells as a precaution, based on reports suggesting that IFNAR2 can signal independently of IFNAR1 (PMID: 10574956; 36118237). We note that it remains unclear whether IFNAR1 deletion alone is sufficient to fully block IFN signaling in BLaER1 cells. In contrast, in mice Ifnar1 deletion is sufficient to abrogate IFN-mediated ISG induction.

Moreover, I assume that "sgMORC3" means that the authors deleted MORC3 using CRISPR/Cas? If so, they should provide Western blotting data to confirm the absence of the MORC3 protein.

R4: The new Fig. S3B confirms loss of MORC3 protein in cells expressing anti-MORC3 sgRNAs.

Furthermore, were the constructs used in Fig. 2E (and those used in similar experiments, e.g., in Fig. 2D) sequence verified?

R5: Yes, all of the reporter constructs used in this study have been validated by whole plasmid sequencing using long read sequencing technology (Plasmidsaurus). Primary data for this can be provided. The methods section was updated to include this.

Fig. S2C and related: Did the authors normalize the copy numbers of the genome-integrated luciferase construct in each experiment using qPCR? Would it not be much easier to insert a genomic isolator sequence between the luciferase cassettes to protect the firefly luciferase from the MRE effect?

R6: To study the effect of MORC3 on luciferase reporters, we first integrated the reporter construct and then transduced with ctrl or MORC3 sgRNA-expressing lentivirus. This ensures that reporter integration levels are identical between conditions when comparing the effect of MORC3 deletion. When comparing different reporter constructs, we include a control reporter of the same length to minimize variation in integration efficiency. To fully exclude residual effects of integration efficiency, we quantified reporter integration by genomic DNA qPCR (Fig. S3E,F). Previously, this only included the Constitutive Luciferase reporter, but we have now added the Minimal Luciferase reporter. All effects remain in the integration-normalized data, confirming that the results cannot be explained by differences in integration. Based on these results, we conclude that differences in integration efficiency do not account for the observed effects, and therefore we did not perform this normalization in subsequent experiments.

The reason we did not use an insulator sequence is that the spread of MORC3-mediated repression to the constitutive luciferase reporter at steady state provided a useful additional readout of repression.

Fig. 2D: Deleting 35 SPI sites may generate a sequence forming an aberrant spatial structure that is different from the original, non-mutated sequence stretch. It would be better to replace base pairs with ones that specifically abolish ETS/SPI1 binding (as for example doi.org/10.1074/jbc.274.8.4939).

R7: We agree with the reviewer that mutating or deleting TF binding sites carries the inherent risk of introducing unintended sequence features, such as novel TF motifs. For this reason, our original SPI motif mutation was designed to not introduce motifs of TFs that are highly expressed in monocytes.

We have now performed the suggested experiment and replaced the GG dinucleotide in the central PU.1 GGAA core motif with the nonbinding variant CA described in the referenced study. Surprisingly, this engineered reporter was active in presence of MORC3 (Fig. R2A). TOMTOM analysis indicated that mutation generated novel binding sites for several transcription factors, including 7 new binding sites for THAP1 (Fig. R2B). Importantly, these motifs are absent from the original MRE sequence and were introduced by the mutation intended to disrupt PU.1 binding. THAP1 is expressed in BLaER1 monocytes and could activate the reporter in presence of MORC3. It seems that MORC3 does not repress the THAP1 motif cluster, which highlights the specificity

of MORC3. In light of these inherent complications of dissecting the regulatory logic of enhancer activties, we hope that the reviewer agrees with our conclusion that mutating the PU.1 binding motifs without creating novel binding sites of different TFs completely abolishes the activities of MRE (Fig. 4D, E).

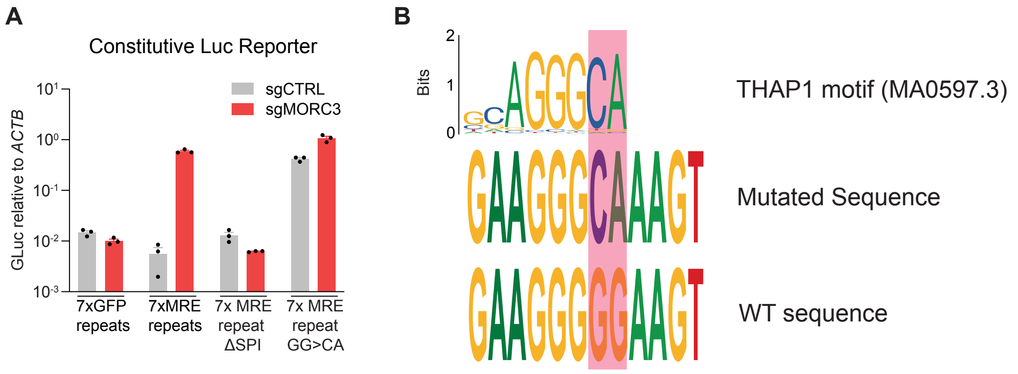

Figure R2: The GG>CA mutation reporter is active in presence of MORC3
A) Indicated Luciferase reporter constructs were integrated into the genome of Cas9 IFNAR1–/– IFNAR2–/– IFNB1-MRE–/– BLaER1 cells. Minimal Promoter Gaussia Luciferase expression upon lentiviral sgRNA delivery is depicted. Replacement of all SPI motifs with random nucleotides abolishes enhancer activity. In contrast, the GG>CA mutation in all SPI motifs generates a constitutively active reporter that is no longer repressed by MORC3.
B) The JASPAR transcription factor motif database was searched for motifs introduced by the GG→CA mutation. A THAP1 binding motif that is created by the mutation but absent in the WT sequence is depicted.

Fig. 4A: Different numbers of complete MRE repeat units were used here. How can the authors conclude from this experiment that the number of SPI1 motifs, but not other sequences in the MRE repeats, determine the specified enhancer/repressor activities?

R8: We agree with the reviewer that this conclusion can not be drawn from the experiment in Fig. 5A (previously Fig. 4A) alone. We have thus rephrased the text: “In conclusion, repeat unit/motif accumulation dictates both repressive and enhancer functions of the IFNB1-MRE. High repeat unit numbers giving rise to high motif numbers allow repression during steady state and synergistic activation upon MORC3 deletion while few repeat units/motifs can only mediate weak enhancer activation.”

However, we are still confident in this conclusion, based on the cumulative evidence from three experiments: In Fig. 4D, E (previously 3D, E) we show that the SPI motifs are required for repressed enhancer activity. In Fig. 5A, S6A (previously 4A, S4A) we show that the repressed enhancer activity correlates with MRE repeat number (including SPI motif numbers) and in Fig. 5B, S6B we show that SPI motifs are sufficient to create a repressed enhancer. To further support this conclusion and to directly test if modulation of the number of SPI motifs correlates with repressed enhancer activity, we have performed an additonal experiment mutating only 2 of the 5 SPI motifs per MRE repeat (Fig. R3). This leads to an intermediate induction of reporter gene expression from the minimal promotor as well as intermediate baseline repression of the constitutive reporter suggesting that SPI motif number correlates with repressed enhancer activity. Together with our observations that the SPI motifs are necesseary and sufficient for repressed enhancer activities , we conclude that other sequences in

the MRE repeats do not have major roles in its activity.

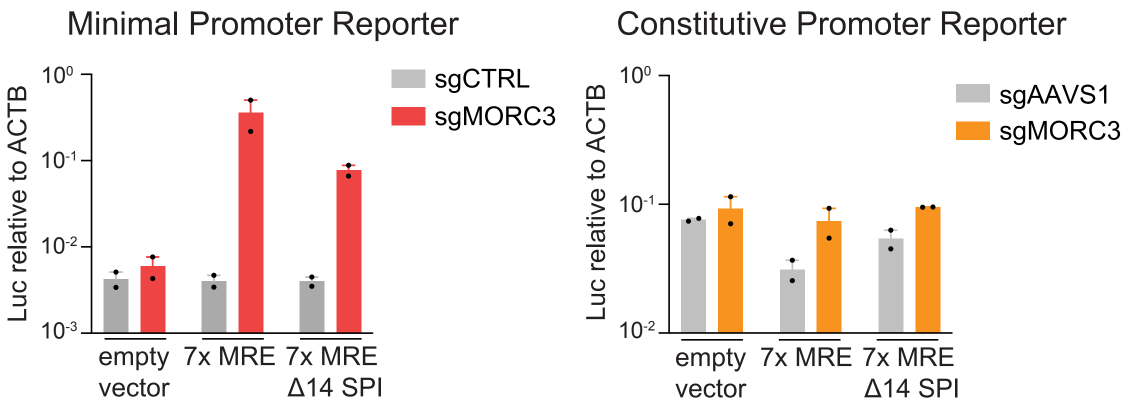

Figure R3: SPI motif numbers correlate with IFNB1-MRE activities
14 SPI motifs in the 7 x consensus IFNB1-MRE (2 per repeat) were scrambled and the construct integrated as Luciferase reporter into the genome of Cas9 IFNAR1$^{-/-}$ IFNAR2$^{-/-}$ IFNB1-MRE$^{-/-}$ BLaER1 cells. Minimal Promoter Gaussia Luciferase or Constitutive Promoter Firefly Luciferase gene expression upon lentiviral sgRNA delivery is depicted.

Fig. 4B: I cannot see any repressive effect mediated by the SPI1 motifs (compare the two gray bars). Therefore, I cannot agree with the authors' conclusion that "large SPI motif clusters are sufficient for MORC3-mediated repression" (lines 216/7).

R9: We thank the reviewer for this comment. There are two reasons why repression by the SPI motifs alone is not apparent in the comparison of the two gray bars in Fig. 5B (old Fig. 4B). First, the non-repetitive random sequence used as a control has very low baseline enhancer activity. As a result, SPI motifs cannot further reduce activity below this minimal background level. Second, as we note in the manuscript, the activity of the “sufficiency” reporter containing 35 SPI motifs is lower than that of the full MRE reporter. This likely reflects additional sequence features, such as motif spacing or flanking context, that contribute to maximal repression.

Importantly, MORC3-dependent repression of a SPI motif cluster is clearly evident at the constitutive promoter in the luciferase assay (Fig. S6B) and most convincingly in the inducible minimal promoter luciferase assay (comparing grey and red bar of 35x PU.1 motif in Fig. 5B). Based on these considerations, we have revised the text to more precisely state that “SPI motif clusters are sufficient to create a MORC3-repressed enhancer.”

Fig. 4D: Does the number of MRE repeats and/or the number of SPI motifs within the MRE correlate with differences in IFNB1 expression in the human population?

R10: This is a very exciting question and future research direction. The presented data is derived from the 1000genome project, which, unfortunately, is anonymized and does not include IFNB1 levels. To answer this question, we would need to determine the

IFNB1 expression levels and sequence IFNB-1MRE locus in the human population, which is not feasible in the scope of the current manuscript.

Fig. 5/Chapter titled "MORC3 suppressed motif clusters of multiple transcription factors." This chapter contains, as I can see, no experimentally verified functional data. All conclusions are based solely on in silico predictions and correlative data. Therefore, the statements are far too strong. The authors should provide experimental validation or tone down their conclusions.

R11: We thank the reviewer for this comment. This chapter applies AI/ML approaches (BPNet) to predict which transcription factor motif clusters may contribute to MORC3's function in limiting chromatin accessibility. The changes in chromatin accessibility itself are experimentally observed using ATAC-seq (Fig. 1E). We have revised the text to clearly distinguish between these experimentally measured effects and the computational predictions (geard at explaining these effects).

Specifically, we now state that MORC3-restricted tandem repeats often contain dense clusters of motifs for several transcription factors, and that BPNet predicts these clusters to be drivers of compaction. We have softened the language to emphasize that these predictions are correlative for the genome-wide analysis. Importantly, we validate these predictions at one tandem repeat, the IFNB1-MRE, where we combine reporter assays, motif perturbations, and PU.1 ChIP-seq to demonstrate that the predicted PU.1 motif clusters are indeed required for enhancer activity upon MORC3 loss. In addition, the BPNet marginalization analysis indicating cluster-mediated repression (now Fig. S7B) is experimentally validated in Fig. 5A.

Finally, we performed PU.1 ChIP-seq in the presence and absence of MORC3, showing that PU.1 recruits MORC3 specifically to tandem repeats containing PU.1 motif clusters, but not to motif clusters of other transcription factors (Fig. 6G). This establishes an experimental correlation between PU.1 motif clusters and the proximal step of MORC3 recruitment within the MORC3–DAXX–H3.3 silencing axis.

Fig. 6: The authors should measure the expression status of MORC3 in PU.1-downregulated cells.

R12: This is shown in Fig. 6C (old Fig. 6B).

In addition, they should also check publicly available ChIP-seq data for PU.1 binding to the MRE in normal macrophages/monocytes.

R13: We thank the reviewer for this exellent suggestion. Indeed, PU.1 is binding to the IFNB1-MRE in primary human macrophages/monocytes. This data is shown in the new Fig. S8A.

Minor:
The authors claim that MORC3 tagging resulted in "mild" hypomorphic activity, leading to "slight" derepression of IFN in the absence of the induction compound. However, as I

see in Fig. S1A, this derepression is >10-fold. I do not believe this is "minor" and as such suggest (although it is not critical to the overall conclusions) that the authors describe this effect more realistically.

R14: As noted by the reviewer, MORC3 tagging alone results in approximately a 10-fold increase in *IFNB1* expression. However, acute degradation of MORC3 induces an additional ~1,000-fold increase. We have revised the text to reflect this more accurately: "Tagging induced a 10-fold de-repression of IFN in absence of 5Ph-IAA, suggesting a hypomorphic activity due to the tag (Fig. S1A). However, rapid MORC3 depletion upon 5Ph-IAA treatment (Fig. 1B) induced 1000-fold expression of *IFNB1* (Fig. 1C), showing that $MORC3^{AID}$ BLaER1 monocytes provide a robust model for investigating MORC3's anti-inflammatory function."

Referee #2:

In "MORC3 represses tandem repeat enhancers to regulate interferon", Krumwiede et al. investigated the mechanism for the MORC3-mediated de-repression of Ifnb1 gene transcription. The authors systematically defined the "IFNB1-MRE" as a region within an intron of the gene Focad that is characterized by several tandem repeats containing dozens of interspersed SPI motifs. The SPI motifs are required and sufficient to recruit MORC3 to the IFNB1-MRE. Through the clever use of degrons linked to MORC3 and the transcription factor PU.1 (knockins and overexpression, respectively), the authors demonstrated that binding of MORC3 to the IFNB1-MRE represses Ifnb1 transcription by presumably preventing accessibility of SPI motifs to PU.1. While the mechanism of repression of IFNB1-MRE by MORC3 is generally well-defined by the authors, many important questions remain, especially with respect to the generality of the MORC3-mediated de-repression of gene expression.

Major Comment 1. The authors make two interconnected claims that are inadequately substantiated by the present data:
(1) "motif clusters are sufficient for MORC3-mediated repression and enhancer activity upon MORC3 loss" (line 26 and others).
(2) a large set of genes are negatively regulated (i.e., repressed) by MORC3 via tandem repeats (lines 104-106 and others "86% of the strongly MORC3-repressed loci...were tandem repeats").
Both of these claims presume that MORC3 broadly represses gene transcription, and MORC3 repression occurs at bound sites containing tandem repeats. However, according to their own gene expression analysis and a previous report in IFN signaling-null cells (Gaidt et al., 2021), very few genes display transcriptional changes upon loss of MORC3 (Fig. S1C), even though MORC3 is found bound to DNA at several locations in the genome (Line 91, >6000 MORC3 ChIP-seq peaks for MORC3). Furthermore, while the authors assert that there are 7 strongly MORC3-repressed loci and 147 total repressed sites (lines 104-105), the referenced data to corroborate that statement is ATACseq (Fig. 1E, F). ATACseq measures chromatin openness, not direct transcriptional activities per se. Since MORC3 is found at promoters with active transcription (i.e., H3K4me3, Li et al., 2016) (Fig. 1D), MORC3 binding to DNA does not

automatically mean a repressive function (which is also clear through their transcriptional analysis). The authors concede that MORC3 binding to DNA, in general, or specifically to tandem repeats does not automatically equate to transcriptional repression (as briefly commented on in lines 101-102 and lines 276-278). Yet throughout this study, MORC3 is framed to have a repressive mechanism that is much more substantial/universal than is believable with the current data as presented.

R15: We agree with the reviewer that MORC3 does not broadly repress gene transcription and did not intend to suggest otherwise. Our data show that MORC3 acts as a highly selective chromatin regulator: it modestly reduces accessibility at many bound loci (new Fig. S1D,E), while strongly restricting accessibility at a small subset of tandem repeats (Fig. 1F). We have revised the text accordingly.

We acknowledge that our use of the term “repression” may have been ambiguous, as we used it both in the context of chromatin accessibility (ATAC-seq) and transcriptional output (RNA-seq). To avoid confusion, we have revised the text to clearly distinguish these concepts. We now use “limiting/restricting chromatin accessibility” when referring to MORC3-mediated decreases in accessibility, and reserve “repression” for effects on enhancer activity or gene expression.

Importantly, only two MORC3-restricted tandem repeats in monocytes function as enhancers that activate adjacent genes upon MORC3 loss. In these specific cases, we maintain that MORC3 represses enhancer activity. This is supported by reporter assays showing that PU.1 motif clusters are sufficient to recreate a MORC3-repressed enhancer (Fig. 5A,B; Fig. S6A,B).

To explain why most motif clusters do not acquire enhancer activity upon MORC3 loss, we focused on SPI motif clusters and identified two limiting factors. First, ~95% of SPI motif clusters are inaccessible and not bound by PU.1 even in the absence of MORC3, indicating MORC3-independent heterochromatinization (Fig. S9I). Second, transcription factor abundance can be limiting: ectopic PU.1 expression is sufficient to activate IFNB1-MRE-regulated genes in PU.1-low cells specifically upon MORC3 loss (Fig. S9D-G). Together, these results indicate that enhancer activation at motif clusters is constrained by redundant chromatin silencing pathways and transcription factor availability. This high specificity is biologically plausible, as viral antagonism of MORC3 triggers a focused IFNB1-centered antiviral response rather than widespread transcriptional activation.

To address the question of actual MORC3 repressive abilities in the context of tandem repeats (beyond the repression of Ifnb1, which is not a novel finding), the authors examined hypothetical/synthetic in silico gene repression by MORC3 (Fig. 5A). However, this data is not convincing. The only in cellulo data provided involves somewhat weak transcriptional changes upon loss of MORC3 for two "strongly MORC3-repressed loci" (Fig. S5C). To help generalize the MORC3 repressive capabilities experimentally, the authors should more deeply examine the evolutionary implications. For example, the authors could use their Minimal Luc reporter with the MRE and MORC3s from different species. A murine monocyte model could also be explored.

R16: We agree with the reviewer that MORC3 is not a broadly acting transcriptional repressor. Instead, our data reveal a selective and previously unknown function: MORC3 reduces chromatin accessibility at a small subset of tandem repeats characterized by dense homotypic motif clusters. This regulation of chromatin accessibility at tandem repeats is not a prediction but an experimentally measured phenomenon, based on the combined analysis of ATAC-seq and MORC3 ChIP-seq (Fig. 1E–F). To our knowledge, such targeted effect of tandem repeats by MORC3 has not been described previously and represents a central conceptual advance of our study. Importantly, we further show that one of these MORC3-regulated tandem repeats functions as a strong enhancer of *IFNB1* upon MORC3 loss. This provides a mechanistic explanation for the potent induction of *IFNB1* observed when MORC3 is inactivated—a phenomenon documented previously but not mechanistically understood.

We also wish to clarify that the in silico component of our study pertains only to predicting which TF motif clusters might drive MORC3-dependent decreased chromatin accessibility. The underlying accessibility data itself is experimentally measured, not predicted. Moreover, even for the motif-based predictions, we perform extensive functional validation using the IFNB1-MRE. Through reporter assays, mutational analyses, and new PU.1 ChIP-seq data, we show that PU.1 motif clusters are indeed sufficient to confer MORC3-mediated silencing and PU.1-dependent enhancer activity.

Finally, addressing the reviewer's suggestion, we validated the main findings in primary mouse bone-marrow–derived macrophages (see R1), demonstrating conservation of the MORC3–PU.1–MRE–IFNB1 axis in primary immune cells and reinforcing the physiological relevance of our conclusions.

In addition, the authors could examine sufficiency by overexpressing PU.1 in other MORC3-deficient cell types that do not otherwise have MORC3-dependent repression of Ifnb1 (see line 28-287).

R17: We thank the reviewer for this excellent suggestion. We have now performed these experiments. Using two naturally PU.1-deficient cell lines (HT-1080 and HCT116), we tested whether PU.1 overexpression is sufficient to activate IFNB1-MRE–regulated genes. In both cell types, PU.1 overexpression induced expression of MRE-activated genes (*IFNB1* and *HACD4*), but only when MORC3 was absent (Fig. S9D-G). These results support the conclusion that PU.1 drives transcription of IFNB1-MRE-regulated genes once MORC3 is removed.

Major Comment 2. Mode of MORC3-repression of PU.1-mediated Ifnb1 transcription is not explained. MORC3 repression of the IFNB1-MRE could occur in a variety of ways to ultimately reduce accessibility of PU.1 to chromatin. What is happening to the IFNB1-MRE by MORC3 that prevents PU.1 accessibility? How can PU.1 be involved in establishing H3K27ac histone marks at the IFNB1-MRE in the presence of MORC3 if MORC3 also limits PU.1 accessibility? These questions need to be answered in order to move this study beyond its current descriptive nature.

R18: We thank the reviewer for highlighting this important mechanistic question. We have now identified how MORC3 represses the IFNB1-MRE, and we have incorporated these new data into the manuscript. Our experiments show that MORC3 recruits the histone H3.3 chaperone DAXX to the IFNB1-MRE (Fig. 3). MORC3 is required for H3.3 deposition at this site, and genetic deletion of *DAXX* leads to MRE-dependent activation of *IFNB1*. These findings are consistent with the MORC3–DAXX–H3.3 pathway previously implicated in silencing endogenous retroviruses in mouse ESCs (PMID: 34650047).

To clarify the role of PU.1, we performed an additional PU.1 ChIP-seq experiment. This analysis shows that PU.1 binds weakly to the IFNB1-MRE even in the presence of MORC3 (Fig. 6G), which explains how PU.1 can contribute to establishing H3K27ac at the MRE under steady-state conditions (Fig. S9C). In the absence of MORC3, PU.1 occupancy is substantially increased.

Together, these data provide a mechanistic framework in which MORC3 maintains the IFNB1-MRE in a compacted chromatin state through DAXX-dependent H3.3 deposition, thereby limiting, but not fully abolishing, PU.1 binding.

Minor Comments.

1. Line 57. "MORC3...inhibits the production of the type I interferon IFN-b" is slightly misleading as MORC3 actually inhibits an enhancer element that can drive Ifnb1 transcription independently of PRR activation. The statement, as is, implies that any pathway to Ifnb1 or IFN-b signaling, itself, is repressed by MORC3.

R19: We agree. The section has been rephrased to state: “MORC3 … inhibits a noncanonical pathway of type I interferon (IFN-b) induction”

2. Line 102. Based on the presented data, MORC3 is not required for repression (the authors say sufficiency here).

R20: We thank the reviewer for this comment. In our view, the distinction between “required” and “sufficient” depends on whether the target locus is actually repressed. If a locus is repressed and remains repressed after MORC3 deletion, MORC3 is not required for that repression. Conversely, if a locus is not repressed despite MORC3 binding, then MORC3 is not sufficient to mediate repression at that site.

The sentence in question refers to MORC3-bound genes that are expressed at varying, and in some cases very high, levels. We therefore consider it more likely that these genes are not repressed, and accordingly, MORC3 is not sufficient to repress them. For this reason, we prefer to retain the original logic. We have revised the wording for clarity as follows: “Transcriptomic analysis of *MORC3*$^{-/-}$ monocytes showed no broad de-repression of genes with MORC3-bound promoters (Fig. S1C) indicating that MORC3 binding is not sufficient to establish repression at promoters.”

3. Line 330-338. For clarity, I suggest revising to "...MORC3-mediated IFN repression in two ways. First, MORC3 repression requires... ...Second, it is likely that not all TFs..."

R21: Rephrased as proposed.

4. Fig 2A. The Human Chr9 schematic could be reworked to be more clear. For example, nucleotide distance to Ifnb1 should be noted.

R22: We have added this info to the figure.

5. Multiple figures (3D, 3E, 6C, 6D, S6F, etc.) have an "ND" or "not detected" notation on the graph over top of sometimes quite large bars. Are these really values that are not detected? If they are truly not detected, then it would be clearer to have no bars shown on the graph and just ND over a blank spot (and the limit of detection can be noted somewhere else, if need be).

R23: In these plots, each dot represents an independent experiment. “ND” was used to indicate that in one or two (but not all three) experiments, the signal fell below the limit of detection. This is a common occurrence in unstimulated conditions where immune or reporter genes are expressed at very low levels but also rarely observed in other contexts.

To avoid confusion, we have removed the “ND” labels from the plots and instead clarify this point in the figure legends.

Referee #3:

In this manuscript, the authors explore the mechanisms through which MORC3 regulates IFNB transcription through negative repression. The senior author has previously described a so-called MORC3-regulated element (MRE) that is situated adjacent to the IFNB locus, and that upon inhibition of MORC3 (by viral virulence factors such as ICP0), IFNB transcription is rapidly upregulated. Interestingly, this transcription is independent of canonical IFN-inducing transcription factors (IRF3/7), while MORC3 is dispensable for canonical IFNB-inducing stimuli. This manuscript addresses the question of how MORC3 preferentially regulates the IFNB locus, and how this cis-regulatory element promotes such robust IFNB induction upon loss of MORC3. The authors begin by characterizing MORC3 binding patterns by performing ChIP-seq. Interestingly, while MORC3 was found to bind to many sites (including H3K4me3 negative sites), loss of MORC3 did not lead to broad de-repression of genes (Fig. S1C), suggesting that binding of MORC3 is not sufficient for repression. ATAC-seq analysis comparison between WT and MORC3-/- BLaER1 monocytes demonstrated that sites of active repression by MORC3 were strongly enriched for tandem repeats. One particular tandem repeat site was identified within the previously defined MRE, and deletion of these tandem repeats ameliorated IFNB induction upon loss of MORC3, suggesting their requirement for MORC3-mediated suppression. Subsequently, using motif analysis, the authors determine that these tandem repeats consist of numerous SPI transcription factor binding motifs, which are required for both repression (MORC3-sufficient) and enhancer activity (upon loss of MORC3). It is important to note that the number of SPI motifs positively correlates with repression. PU.1, an SPI member with

high expression in monocytes, was hypothesized to be the TF responsible for MORC3-mediated IFNB transcriptional control. Indeed, inducible deletion of PU.1 extinguished IFNB induction upon loss of MORC3, while having no effect on IFNB induction after STING activation, confirming its specific role in the MORC3-mediated pathway. Additionally, deletion of PU.1 decreased MORC3 binding to the MRE (via ChIP-qPCR), suggesting a mechanism wherein PU.1 binding to the MRE facilitates recruitment of MORC3 for negative regulation of IFNB at steady state, and loss of MORC3 enables PU.1-mediated transcription of IFNB.
In summation, this manuscript defines the mechanisms through which the previously described MRE facilitates rapid IFNB induction upon loss of MORC3. The authors nicely demonstrate, using a variety of molecular tools, the enrichment of SPI motif-containing tandem repeats at the IFNB-MRE that is critical for MORC3-mediated repression and that PU.1 is a critical TF for both repression and enhancement upon loss of MORC3. The study however has important gaps that should be addressed.

Major issues:
The biggens concern I have is that experiments are primarily done using the human BLaER1 monocytes and in order to make sure that these are not cell line specific findings, it is critical for the authors to knock down MORC3 in primary human monocytes and examine induction of IFNb and ISGs.

R24: We thank the reviewer for this important point. We previously demonstrated that Cas9:RNP-mediated deletion of *MORC3* in primary human monocyte-derived macrophages induces *IFNB1* and ISGs (Gaidt et al., 2021). To address the reviewer's concern in the context of the present study, we have now validated the main conclusions of our manuscript in primary mouse macrophages.

We selected mouse macrophages for these mechanistic studies because we had access to *Ifnar1*$^{-/-}$ mice. This allowed us to efficiently prevent secondary ISG induction during MORC3 perturbation, which is essential for interpreting the chromatin- and enhancer-focused experiments performed here. We summarize the results from these new experiments here (detailed response in R1, new Fig. 7): MORC3 deletion in primary bone-marrow–derived macrophages activates *Ifnb1* and MRE-adjacent genes, MORC3 antagonizes PU.1 binding to the mouse IFNB1-MRE, and PU.1 is required for MORC3-dependent control of *Ifnb1* expression. These findings confirm the central conclusions of our study in a primary, non-cell line system.

Additionally, it is critical for the authors to examine other inflammatory cytokines that are not type I IFNs especially in the context of PU.1 knockdown. What is the status of Nf-kB dependent genes such as TNF, IL-1b and IL-6 when PU.1 is not present and also how do these cells respond to STING agonists and TLR agonists. In the absence of this specificity, it would be hard to conclude that in the absence of MORC3, there is specific induction of IFNb by PU.1.

R25: We thank the reviewer for these valid suggestions. We have now performed the requested experiments. As shown in Fig. 6D, PU.1-depleted BLaER1 monocytes

respond normally to the STING agonist diABZI. In addition, TLR responses in PU.1-depleted BLaER1 monocytes are intact (Fig. S9B), including induction of the NF-κB–responsive genes TNF, IL1B, and IL6. Finally, PU.1-deleted primary mouse macrophages also mount a normal response to STING stimulation (Fig. 7C).

Together, these results demonstrate that PU.1 is not required for IRF3/7-dependent canonical IFN induction or for TLR-mediated NF-κB activation, and they support the conclusion that PU.1 specifically mediates the MRE-dependent pathway to IFNB1.

Is the IFNB-MRE the only MORC3-bound site with Tandem Repeats/SPI motifs?

R26: No. As shown in Fig. 1F, several tandem repeats are bound by MORC3, which limits chromatin accessibility. Some of these repeats harbor SPI motif clusters, whereas others contain motif clusters for different transcription factors (Fig. 6G).

What is required for MORC3-binding to genomic regions? Is it Tandem repeats, is it H3K4me3?

R27: We thank the reviewer for this question. To determine what drives MORC3 binding to tandem repeats, we performed a new MORC3 ChIP-seq experiment in absence and presence of PU.1. This analysis showed that PU.1 recruits MORC3 specifically to tandem repeats containing dense PU.1 motif clusters, but not to tandem repeats enriched for KLF/SP or AP-1 motifs (Fig. 6G). In contrast, PU.1 depletion did not reduce MORC3 binding at sites with few PU.1 motifs, such as promoters. Together, these data indicate that, in the context of tandem repeats, MORC3 recruitment is mediated by transcription factors and correlates with the presence of motif clusters. Our future work will aim to elucidate the molecular mechanisms by which transcription factors recruit MORC3 to motif clusters and to determine whether this process is linked to H3K4me3.

The authors state that "MORC3 represses tandem repeats enriched with ETS, AP-1, or SP/KLF motifs, likely due to the high density of these motifs" How does this fit with lack of broad gene upregulation seen in Fig. S1C (i.e., shouldn't loss of MORC3 lead to upregulation of additional genes containing these motifs?

R28: This is an important question. Our data indicate that MORC3 restricts chromatin accessibility strongly at motif clusters, consistent with our finding that PU.1 recruits MORC3 selectively to clustered, but not isolated, transcription factor binding sites. However, this does not explain why most motif clusters do not function as enhancers of neighboring genes upon MORC3 loss.

To address this, we focused on PU.1 motif clusters and identified two additional limiting factors. First, genome-wide analysis revealed that the majority (~95%) of PU.1 motif clusters are not bound by PU.1, even in the absence of MORC3, and they lack chromatin accessibility as determined by ATAC-seq (Fig. S9I). This indicates that other heterochromatinization mechanisms act at these sites independently of MORC3. Second, we tested whether transcription factor availability is limiting. In cell types with

low or absent PU.1 expression, the PU.1 motif cluster at the IFNB1-MRE fails to exhibit enhancer activity; however, ectopic PU.1 expression is sufficient to activate IFNB1-MRE–regulated genes (Fig. S9D-G). This demonstrates that transcription factor levels can be a critical determinant of enhancer activation. We speculate that this could also be the case for other motif clusters. Together, these results indicate that enhancer activation of motif clusters is constrained by redundant chromatin silencing pathways and by transcription factor availability. Additional factors, such as promoter–enhancer compatibility or three-dimensional genome organization, are also likely to contribute but were not directly addressed here. Biologically, this high degree of specificity is plausible: during DNA virus infection, MORC3 is frequently antagonized, and its loss triggers a focused antiviral response centered on *IFNB1* rather than a broad transcriptional program, which could be detrimental. In summary, MORC3 restricts accessibilty only a limited subset of tandem repeats with motif clusters, and among these, only a very small fraction acquire enhancer activity upon MORC3 loss, accounting for the minimal global transcriptional changes observed.

Minor Issues:
- MORC3 binds to non-Tandem repeat promoter regions affecting chromatin accessibility, but loss of MORC3 does not lead to de-repression of adjacent genes. In these settings, what is MORC3 doing upon binding if not repressing? (Fig. S1C-D).

R29: We thank the reviewer for this keen observation. Our new cumulative analyses show that MORC3 binding is associated with a broad but very subtle reduction in chromatin accessibility that is detectable across many MORC3-bound sites, including promoters (new analyses in Fig. S1E, F). While this effect is difficult to discern at individual loci, it becomes apparent when MORC3-bound regions are analyzed collectively, indicating that MORC3 modestly influences chromatin structure wherever it binds, largely independent of genomic context.

We have now identified the genetic mechanism underlying strong MORC3-mediated compaction at the IFNB1-MRE: MORC3 recruits the histone chaperone DAXX and is required for H3.3 incorporation at this tandem repeat (new Fig. 3). We speculate that a related MORC3–DAXX–H3.3 pathway also operates at promoters. However, in contrast to tandem repeats, H3.3 incorporation at promoters is widely associated with active transcription rather than repression (Ahmad & Henikoff, 2002; Mito et al., 2005). Thus, MORC3-mediated H3.3 deposition at promoters would be expected to subtly alter chromatin accessibility without repressing transcription. Notably, H3.3 deposition at active promoters is classically attributed to the HIRA chaperone complex, and our future work will investigate how MORC3–DAXX–H3.3 and HIRA–H3.3 pathways differentially contribute to promoter chromatin organization and how H3 tail post-translational modifications contribute to these effects.

In summary, MORC3 mildly modulates chromatin accessibility at promoters but does not function as a transcriptional repressor at these sites, consistent with the established role of H3.3 at active genes.

- Authors state "MORC3-mediated silencing can spread to adjacent active genetic

elements" (Fig. 2F) but there is no evidence of this in their initial analysis of MORC3 bound promoters (Fig. S1C).

R30: We agree with the reviewer. The original statement was based solely on the reporter construct and should not be generalized to endogenous genomic loci. We have revised the text to clarify that in this experimental context, “MORC3-mediated repression can spread to the adjacent active reporter gene.”

In the genome, MREs are located far from their target genes (e.g., the IFNB1-MRE lies ~100 kb downstream of *IFNB1*), and we do not observe spreading of silencing: genes adjacent to the endogenous IFNB1-MRE are not upregulated upon MRE deletion. In contrast, in the reporter assay, the constitutive luciferase promoter is positioned only 116 bp from the inserted MRE, and MORC3-mediated repression extends into this immediately adjacent reporter (Fig. 2F).

- Fig. 3D/E shows that the 35 SPI motifs are critical for MORC3 mediated suppression but would have been nice to show this IFNB transcriptional levels as opposed to luciferase activity (if possible to do?) Same with Fig. 4A

R31: We appreciate the reviewer’s suggestion and agree that measuring *IFNB1* transcription directly at the endogenous locus would be highly informative. However, this is technically very challenging. The repetitive sequence of the IFNB1-MRE prevents specific genome engineering, becauses guide RNAs designed against one repeat unit inevitably cut all others, likely impacting locus stability. SPI motifs within the repeat therefore cannot be selectively edited. In addition, large genomic edits are extremely inefficient in BLaER1 monocytes, particularly those requiring homology-directed repair. For example, generating a deletion of the IFNB1-MRE required screening approximately 400 monoclonal cell lines.

Importantly, our key conclusions regarding the role of PU.1 are independently supported by experiments at the endogenous IFNB1 locus, including PU.1 loss- and gain-of-function studies. We therefore believe that the reporter-based analyses provide a robust and appropriate strategy for motif-level dissection, given the substantial technical challenges inherent to manipulating repetitive genomic elements.

- Are SPI transcription factors bound to IFNB-MRE at steady state via ChIP seq?
o CoIP experiments would be good to examine MORC3 binding to PU.1

R32: Yes. As shown in Fig. 6A, PU.1 is bound to the IFNB1-MRE at steady state, and its occupancy increases upon loss of MORC3.

We also performed co-immunoprecipitation experiments using both anti-V5-MORC3 and anti-PU.1 antibodies in BLaER1 monocytes (endogenous expression of targets). These experiments did not detect direct MORC3–PU.1 interactions. Given that MORC3 limits accessibility of tandem repeats containing motif clusters for several different transcription factors, we consider it unlikely that MORC3 directly binds to each of these

TFs. As discussed in R29 the molecular mechanism by which PU.1 (and hypothetically other TFs) recruit MORC3 specifically to motif clusters is our future research aim.

- Important to demonstrate that loss of SPI motifs did not affect canonical IFNB-inducing stimuli (IRF3/7 dependent pathway)

R33: We agree with the reviewer. We have tested whether loss of IFNB1-MRE containing the SPI motifs affects canonical IRF3/7-dependent *IFNB1* induction, and the response remains intact. This is shown in Fig. S3C.

- Authors state no allele in human data set had less than 7 repeats and reference Fig 4A/S4A but that is not that data)

R34: We thank the reviewer for pointing out this ambiguity. We have corrected the text to read: “no allele in the 1000 genomes dataset had less than 7 repeats (Fig. 5D) which is sufficient for MORC3-mediated repression (Fig. 5A, S6A)”

Table of changes:

Figure	Description
Fig. 1	New replicates in anti-V5 (MORC3) ChIP allowed detection of more MORC3 peaks (ca 8000 now). Data was reanalysed accordingly.
Fig. S1	New cumulative analysis shows that MORC3 mildly restricts chromatin accessibility at bound sites. New data showing that significant decompaction of specific ERV families is was not observed.
Fig. S2	New data validating our BWA based read-mapping strategy to VNTRs by comparing it to the specialized computational method dangbing-tk.
Fig. 2	No change
Fig. S3	Previously Fig. S2<br>New data confirming the loss of MORC3 protein after lentiviral delivery of sgRNAs into Cas9 -expressing BlaER1 cells.<br>New data showing deletion of the IFNB1-MRE tandem repeat does not change responses to a STING agonist.<br>New Luciferase reporter data normalized to genomic integration.
**Fig. 3**<br>**Fig. S4**	**New data discovering the genetic mechanism of IFNB1-MRE repression: MORC3 recruits DAXX to incorporate H3.3.**
Fig. 4	Previously Fig. 3
Fig. S5	Previously Fig. S3
Fig. 5	Previously Fig. 4
Fig. S6	Previously Fig. S4
Fig. S7	Previously Fig. 5 and S5.<br>We reran the attribution analysis with more sensitive settings. This identified a few more tandem repeats with motif clusters.
Fig. 6	New PU.1 ChIP-seq data in presence or absence of MORC3 shows that PU.1 recruits MORC3 to tandem repeats with SPI motif clusters

	but not to PU.1 bound promotors or tandem repeats with other motif clusters.
Fig. S8	New data that PU.1 binds to the IFNB1-MRE in human primary myeloid cells.<br>New PU.1 ChIP-Seq data showing that PU.1 occupancy across the genome is generally not affected by loss of MORC3.
Fig. S9	Previously Fig. S6.<br>New data showing that TLR-responses are not impaired upon PU.1 depletion.<br>New data showing that PU.1 overexpression in PU.1^{low} cell types leads to mild IFNB1-MRE-regulated gene activation specifically upon MORC3 depletion.<br>New V5 (MORC3) ChIP-Seq in absence of PU.1 shows that binding of MORC3 is not disrupted genome wide by loss of PU.1<br>New data showing that large SPI motif clusters are rare in the human genome and mostly not accessible and not bound by PU.1.
**Fig. 7**<br>**Fig. S10**	**New data validating our main model in primary mouse macrophages.**

Dear Dr. Gaidt,

Thank you for submitting a revised version of your manuscript. Your study has now been seen by all original referees, who find that their previous concerns have been addressed and now recommend publication of the manuscript pending several minor textual revisions and clarifications which I would ask you to address in a revised manuscript. In addition, there remain only a few mainly editorial points that have to be addressed before I can extend formal acceptance of the manuscript:

- Please rename the "Data and materials availability" section to Data Availability and place it before the Acknowledgments section

- Please rename the "Conflict of Interest" section into "Disclosure and Competing Interests Statement", in accordance with our updated Guide to Authors (https://link.springer.com/partners/embo-press/editorial-policies#Competing%20interest%20disclosures)

- AUTHORS: name discrepancy - Karina Schindler-Schumitsch in the manuscript vs. Karina Schindler in the system

- AFFILIATIONS: InstaDeep and Tempest Therapeutics seem to be companies; employment in a biotech company should be stated in DCIS

- As we are switching from a free-text author contribution statement towards a more formal statement based on Contributor Role Taxonomy (CRediT) terms, please remove the present Author Contribution section and instead specify each author's contribution(s) directly in the Author Information page of our submission system during upload of the final manuscript. See https://casrai.org/credit/ for more information.

- Please provide either a "Yes" or a "Not Applicable" answer to each one of the questions in your Author Checklist (https://media.springernature.com/original/springer-cms/rest/v1/content/27825796/data/v1). In the last column of this checklist, only the sections of the manuscript where the relevant information can be found should be listed (the information per se should be included in the main manuscript file).

- Please remove "and Funding" from the Acknowledgments section heading in the manuscript

- DATASET EV LEGENDS: 3 suppl. tables uploaded as datasets, but the nomenclature is wrong, should be Dataset EV1-Dataset EV3 and this should be corrected in all places (source file names, titles in the system, individual Excel files, figure legends, callouts in the manuscript); the legends should be removed from the manuscript and each should be provided in a separate sheet/tab in the Excel

- APPENDIX FILE WITH ToC: not provided, but perhaps some of the suppl. figures can be combined into an Appendix PDF (Appendix Figure S1, etc.)

- Please provide suggestions for a short 'blurb' text prefacing and summing up the conceptual aspect of the study in two sentences (max. 250 characters), followed by 3-5 one-sentence 'bullet points' with brief factual statements of key results of the paper; they will form the basis of an editor-written 'Synopsis' accompanying the online version of the article. Please also provide an altered synopsis image, making sure that the aspect ratio conforms to our website's format - it should be exactly 550 pixels wide and between 300-600 pixels high.

- Please provide the Reagent and Tools Table. For more information, please check
https://media.springernature.com/original/springer-cms/rest/v1/content/27825802/data/v1
Maybe some of Table S3 can be part of the Reagents table

- Please provide SOURCE DATA. The source data request with more details will be sent in a separate e-mail shortly

- Supplementary Materials section with the table legends should be removed from the manuscript

- The manuscript sections should be in the following order: Title page - Abstract & Keywords - Introduction - Results - Discussion - Methods - Data Availability - Acknowledgments - Disclosure Statement & Competing Interests - References - Figure Legends - (Main Tables with legends if applicable) - Expanded View Figure Legends.

- Supplementary figures need to be updated to Figure EV1, etc. in all places (source file names, titles in the system, individual figure files, figure legends, callouts in the manuscript)

- Main and EV figure legends should not be mixed - we need main figure legends listed and then EV figure legends should follow

- The resolution of the submitted figures is too low for blot and images. Specifically, Figures 1B, 6C, Appendix S3B, S4A & B, S9 D & F, S10 C. This reduction in resolution is commonly caused by converting original 16-bit TIFF files to RGB format for publication. While this is not inherently problematic, it can raise concerns about image integrity for critical readers. To avoid any misunderstanding and to meet EMBO Press standards, we kindly ask that you:

* Resubmit the above highlighted figures at the captured original data resolution.
* Please supply the blot source data files at the original captured resolution.

- Please note that the specific URL for GSE301805 dataset is not provided in the data availability statement.
- Comments to figures and figure legends:

1. Please note that the box plots need to be defined in terms of minima, maxima, center, bounds of box and whiskers, and percentile in the legends of figures 6F

2. Please note that information related to n is missing in the legends of figures 1E

3. Please note that n=2 in figures 6A, E

4. Please note that the exact p values are not provided in the legends of figures 1C, 2B, D, E, F; 3A, C; 4D, E; 5A, B; 6D, 7A-D.

5. Please indicate the statistical test used for data analysis in the legend of figure 1E

Please let me know if you have any questions regarding any of these points. You can use the link below to upload the revised files.
Thank you again for giving us the chance to consider your manuscript for The EMBO Journal. I look forward to receiving the final version.
With best regards,
Cornelius Schneider

____
Cornelius Schneider, PhD
Editor | The EMBO Journal
c.schneider@embojournal.org

Please refer to our figure preparation guideline in order to ensure proper formatting and readability in print as well as on screen:

https://link.springer.com/journal/44318/submission-guidelines#cms-Figure-and-data-presentation

Use the link below to submit your revision:

https://emboj.msubmit.net/cgi-bin/main.plex

Referee #1:

The authors have done a superb job addressing my critique points. In particular, the verification of their findings in primary macrophages was important and substantially strengthened the manuscript. The only remaining concern relates to the Cas9/CRISPR studies, which appear to have been conducted using only a single sgRNA, or a single sgRNA pair, per targeted locus, raising the possibility of off-target effects. If multiple independent sgRNAs were used for each locus, the authors should state this more explicitly, as doing so would further enhance the reliability of their conclusions.

Referee #2:

In this revised manuscript, the authors have addressed all concerns.

Referee #3:

In this manuscript, the authors explore the mechanisms through which MORC3 regulates IFNB transcription through negative repression. The authors have done a commendable job addressing the concerns raised during the review of the previous version of this manuscript. Overall this is an important manuscript that makes significant contributions to understanding the role of MORC3 in repression of IFNb.

a. The authors have previously demonstrated that knock-down of MORC3 in primary human monocyte-derived macrophages induces IFNB1 and ISGs (Gaidt et al., 2021). However, to satisfy this reviewer's comment regarding primary cells, the authors used primary bone marrow-derived macrophages (BMMs) from Ifnar1-/- mice, to prevent secondary IFN effects. Using Cas9:RNP knock-down in Ifnar1-/- BMMs they demonstrate that loss of MORC3 drives induction of Ifnb1. ChIP-qPCR also revealed that loss of MORC3 lead to enhanced binding of PU.1 to the MRE, but had no effect on other PU.1 binding sites (i.e., NLRP3, SPI1p). In line with data using BlaER1 cell line, loss of MORC3 and SPI1 together abrogates the induction of Ifnb1 transcription, but loss of SPI1 has no effect on STING-induced Ifnb1 transcriptional upregulation. These data, along with previously published data in primary human monocyte-derived macrophages satisfy the point.

b. The authors have now performed the requested experiments to look at additional pro-inflammatory cytokines. Figure 6D demonstrates normal Ifnb1 induction in response to STING activation in PU.1-depleted BlaER1 monocytes. Figure 7C demonstrates normal Ifnb1 induction in response to STING activation in PU.1-depleted murine BMMs. Figure S9B demonstrates normal TLR responses in PU.1 depleted BlaER1 monocytes.

c. New cumulative data suggest that MORC3 binding is associated with a broad but very subtle reduction in chromatin accessibility (Fig. S1E and S1F). Additionally, the authors have now identified the genetic mechanism underlying strong MORC3-mediated compaction at the IFNB1-MRE.

d. New Figure 3 demonstrates that MORC3 recruits DAXX, which incorporates H3.3 at the INFB-MRE tandem repeat, leading to suppression. Data demonstrated that knock down of DAXX phenocopies loss of MORC3, resulting in increased transcription of Ifnb1.

e. Figure 6A suggests SPI TFs are bound at steady state and residence is increased upon loss of MORC3

f. The authors performed CoIP experiments in BLaER1 monocytes and did not detect direct PU.1-MORC3 interactions

g. The authors nicely demonstrate in Fig. S3C that upon broad deletion or targeted deletion of certain repeats, Ifnb1 transcription in response to STING activation is conserved

h. Finally, the authors have revised the text to correct ambiguity of tandem repeats

Minor comments below should be addressed

o Abstract line 27 states 35 PU.1 binding sites, intro line 68 states 45 PU.1 binding sites
o Results line 391 states CD11b and F4/80 expression is depicted in S9A/B, but this data is in S10A/B (same with line 392 S10C not S9C)

We thank the reviewers and the editors for their comments and suggestions. We have implemented all of them. Detailed responses below.

Referee #1 (Report for Author)

The authors have done a superb job addressing my critique points. In particular, the verification of their findings in primary macrophages was important and substantially strengthened the manuscript. The only remaining concern relates to the Cas9/CRISPR studies, which appear to have been conducted using only a single sgRNA, or a single sgRNA pair, per targeted locus, raising the possibility of off-target effects. If multiple independent sgRNAs were used for each locus, the authors should state this more explicitly, as doing so would further enhance the reliability of their conclusions.

This is now mentioned in the manuscript.

Referee #2 (Report for Author)

In this revised manuscript, the authors have addressed all concerns.

Referee #3 (Report for Author)

In this manuscript, the authors explore the mechanisms through which MORC3 regulates IFNB transcription through negative repression. The authors have done a commendable job addressing the concerns raised during the review of the previous version of this manuscript. Overall this is an important manuscript that makes significant contributions to understanding the role of MORC3 in repression of IFNb.

a. The authors have previously demonstrated that knock-down of MORC3 in primary human monocyte-derived macrophages induces IFNB1 and ISGs (Gaidt et al., 2021). However, to satisfy this reviewer's comment regarding primary cells, the authors used primary bone marrow-derived macrophages (BMMs) from Ifnar1-/- mice, to prevent secondary IFN effects. Using Cas9:RNP knock-down in Ifnar1-/- BMMs they demonstrate that loss of MORC3 drives induction of Ifnb1. ChIP-qPCR also revealed that loss of MORC3 lead to enhanced binding of PU.1 to the MRE, but had no effect on other PU.1 binding sites (i.e., NLRP3, SPI1p). In line with data using BlaER1 cell line, loss of MORC3 and SPI1 together abrogates the induction of Ifnb1 transcription, but loss of SPI1 has no effect on STING-induced Ifnb1 transcriptional upregulation. These data, along with previously published data in primary human monocyte-derived macrophages satisfy the point.

b. The authors have now performed the requested experiments to look at additional pro-inflammatory cytokines. Figure 6D demonstrates normal Ifnb1 induction in response to STING activation in PU.1-depleted BlaER1 monocytes. Figure 7C demonstrates normal Ifnb1 induction in response to STING activation in PU.1-depleted murine BMMs. Figure S9B demonstrates normal TLR responses in PU.1 depleted BlaER1 monocytes.

c. New cumulative data suggest that MORC3 binding is associated with a broad but very subtle reduction in chromatin accessibility (Fig. S1E and S1F). Additionally, the

authors have now identified the genetic mechanism underlying strong MORC3-mediated compaction at the IFNB1-MRE.

d. New Figure 3 demonstrates that MORC3 recruits DAXX, which incorporates H3.3 at the INFB-MRE tandem repeat, leading to suppression. Data demonstrated that knock down of DAXX phenocopies loss of MORC3, resulting in increased transcription of Ifnb1.

e. Figure 6A suggests SPI TFs are bound at steady state and residence is increased upon loss of MORC3

f. The authors performed CoIP experiments in BLaER1 monocytes and did not detect direct PU.1-MORC3 interactions

g. The authors nicely demonstrate in Fig. S3C that upon broad deletion or targeted deletion of certain repeats, Ifnb1 transcription in response to STING activation is conserved

h. Finally, the authors have revised the text to correct ambiguity of tandem repeats

Minor comments below should be addressed

o Abstract line 27 states 35 PU.1 binding sites, intro line 68 states 45 PU.1 binding sites
o Results line 391 states CD11b and F4/80 expression is depicted in S9A/B, but this data is in S10A/B (same with line 392 S10C not S9C)

This is changed in the manuscript.

- Please rename the "Data and materials availability" section to Data Availability and place it before the Acknowledgments section

This is changed in the manuscript.

- Please rename the "Conflict of Interest" section into "Disclosure and Competing Interests Statement", in accordance with our updated Guide to Authors (https://link.springer.com/partners/embo-press/editorial-policies#Competing%20interest%20disclosures)

This is changed in the manuscript.

- AUTHORS: name discrepancy - Karina Schindler-Schumitsch in the manuscript vs. Karina Schindler in the system

Yes, this should be changed in the online system. It's the same person, but the record in the online system is not up to date. I talked with Karina and she said that she doesn't remember to have an account with EMBO to change this and I don't have the permission to do this. Are you able to do this in the system? Many thanks!

- AFFILIATIONS: InstaDeep and Tempest Therapeutics seem to be companies; employment in a biotech company should be stated in DCIS

DCIS was updated to state: “Dara L Burdette was a stockholder and employee at Tempest Therapeutics. Moritz M Gaidt is inventor of a patent related to this work. Work in the Gaidt laboratory was supported by Boehringer Ingelheim and Tempest Therapeutics.” Bernardo P de Almeida contributed to this manuscript as a PhD student at the IMP and currently works for InstaDeep. His current work is not connected to this mansucript. Does his current affiliation represent a conflict of interest that should be disclosed in the DCIS?

- As we are switching from a free-text author contribution statement towards a more formal statement based on Contributor Role Taxonomy (CRediT) terms, please remove the present Author Contribution section and instead specify each author's contribution(s) directly in the Author Information page of our submission system during upload of the final manuscript. See https://casrai.org/credit/ for more information.

This is changed in the manuscript.

- Please provide either a "Yes" or a "Not Applicable" answer to each one of the questions in your Author Checklist (https://media.springernature.com/original/springer-cms/rest/v1/content/27825796/data/v1). In the last column of this checklist, only the sections of the manuscript where the relevant information can be found should be listed (the information per se should be included in the main manuscript file).

Author checklist is provided with this submission.

- Please remove "and Funding" from the Acknowledgments section heading in the manuscript

This is changed in the manuscript.

- DATASET EV LEGENDS: 3 suppl. tables uploaded as datasets, but the nomenclature is wrong, should be Dataset EV1-Dataset EV3 and this should be corrected in all places (source file names, titles in the system, individual Excel files, figure legends, callouts in the manuscript); the legends should be removed from the manuscript and each should be provided in a separate sheet/tab in the Excel

This is changed in the manuscript.

- APPENDIX FILE WITH ToC: not provided, but perhaps some of the suppl. figures can be combined into an Appendix PDF (Appendix Figure S1, etc.)

We have kept the original Figures as EV1-10. I hope this is fine.

- Please provide suggestions for a short 'blurb' text prefacing and summing up the conceptual aspect of the study in two sentences (max. 250 characters), followed by

3-5 one-sentence 'bullet points' with brief factual statements of key results of the paper; they will form the basis of an editor-written 'Synopsis' accompanying the online version of the article. Please also provide an altered synopsis image, making sure that the aspect ratio conforms to our website's format - it should be exactly 550 pixels wide and between 300-600 pixels high.

Blurb and synopsis image are provided with this submission.

- Please provide the Reagent and Tools Table. For more information, please check https://media.springernature.com/original/springer-cms/rest/v1/content/27825802/data/v1
Maybe some of Table S3 can be part of the Reagents table

Reagent and tools table is provided with this submission.

- Please provide SOURCE DATA. The source data request with more details will be sent in a separate e-mail shortly

Source data is provided with this submission.

- Supplementary Materials section with the table legends should be removed from the manuscript

This is changed in the manuscript.

- The manuscript sections should be in the following order: Title page - Abstract & Keywords - Introduction - Results - Discussion - Methods - Data Availability - Acknowledgments - Disclosure Statement & Competing Interests - References - Figure Legends - (Main Tables with legends if applicable) - Expanded View Figure Legends.

This is changed in the manuscript.

- Supplementary figures need to be updated to Figure EV1, etc. in all places (source file names, titles in the system, individual figure files, figure legends, callouts in the manuscript)

This is changed in the manuscript.

- Main and EV figure legends should not be mixed - we need main figure legends listed and then EV figure legends should follow

This is changed in the manuscript.

- The resolution of the submitted figures is too low for blot and images. Specifically, Figures 1B, 6C, Appendix S3B, S4A & B, S9 D & F, S10 C. This reduction in resolution is commonly caused by converting original 16-bit TIFF files to RGB format for publication.
While this is not inherently problematic, it can raise concerns about image integrity for critical readers. To avoid any misunderstanding and to meet EMBO Press

standards, we kindly ask that you:

* Resubmit the above highlighted figures at the captured original data resolution.
* Please supply the blot source data files at the original captured resolution.

All figures are now uploaded at high resolution. Raw blot images are part of the source data.

- Please note that the specific URL for GSE301805 dataset is not provided in the data availability statement.

This is changed in the manuscript.

- Comments to figures and figure legends:

1. Please note that the box plots need to be defined in terms of minima, maxima, center, bounds of box and whiskers, and percentile in the legends of figures 6F

This info has been added.

2. Please note that information related to n is missing in the legends of figures 1E

This info has been added.

3. Please note that n=2 in figures 6A, E

Yes, these are very labor-intense experiments and can encompass up to 5 x 10^8 cells per replicate and condition. In this light, we believe that our conclusions are justified by n = 2 independent experiments. This issue has also not been raised in the peer-review process.

4. Please note that the exact p values are not provided in the legends of figures 1C, 2B, D, E, F; 3A, C; 4D, E; 5A, B; 6D, 7A-D.

P values have now been added to all figure legends.

5. Please indicate the statistical test used for data analysis in the legend of figure 1E

This info has been added to all figure legends.

2nd Revision - Editorial Decision 7th Apr 2026

Dear Dr. Gaidt,

I am pleased to inform you that your manuscript has been accepted for publication in the EMBO Journal.

Your manuscript will be processed for publication by EMBO Press. It will be copy edited and you will receive page proofs prior to publication. Please note that you will be contacted by Springer Nature Author Services to complete licensing and payment information.

You may qualify for financial assistance for your publication charges - either via a Springer Nature fully open access agreement or an EMBO initiative. Check your eligibility: https://link.springer.com/journal/44318/how-to-publish-with-us

Should you be planning a Press Release on your article, please get in contact with embo_production@springernature.com as early as possible in order to coordinate publication and release dates.

If you have any questions, please do not hesitate to contact the Editorial Office. Thank you for your contribution to The EMBO Journal.

Yours sincerely,

Cornelius Schneider, PhD
Editor
The EMBO Journal
c.schneider@embojournal.org

*****
Please note that it is The EMBO Journal policy for the transcript of the editorial process (containing referee reports and your response letters) to be published as an online supplement to each paper. If you should prefer removal of any referee-only figures included in the point-by-point response(s), e.g. because they may still be used for future publication or because they have been reproduced from published work by others, please do let us know immediately via response email.
More information is available here: https://link.springer.com/partners/embo-press/editorial-policies#Peer%20review

** Click here to be directed to your login page: https://emboj.msubmit.net

EMBO Press Author Checklist

Corresponding Author Name: Moritz Gaidt
Journal Submitted to: The EMBO Journal
Manuscript Number: EMBOJ-2025-121873R

USEFUL LINKS FOR COMPLETING THIS FORM
The EMBO Journal - Author Guidelines
EMBO Reports - Author Guidelines
Molecular Systems Biology - Author Guidelines
EMBO Molecular Medicine - Author Guidelines

Reporting Checklist for Life Science Articles (updated January
This checklist is adapted from Materials Design Analysis Reporting (MDAR) Checklist for Authors. MDAR establishes a minimum set of requirements in transparent reporting in the life sciences (see Statement of Task: 10.31222/osf.io/9sm4x). Please follow the journal's guidelines in preparing your
Please note that a copy of this checklist will be published alongside your article.

Abridged guidelines for figures
1. Data
The data shown in figures should satisfy the following conditions:
- ➞ the data were obtained and processed according to the field's best practice and are presented to reflect the results of the experiments in an accurate and unbiased manner.
- ➞ ideally, figure panels should include only measurements that are directly comparable to each other and obtained with the same assay.
- ➞ plots include clearly labeled error bars for independent experiments and sample sizes. Unless justified, error bars should not be shown for technical
- ➞ if n<5, the individual data points from each experiment should be plotted. Any statistical test employed should be justified.
- ➞ Source Data should be included to report the data underlying figures according to the guidelines set out in the authorship guidelines on Data

2. Captions
Each figure caption should contain the following information, for each panel where they are relevant:
- ➞ a specification of the experimental system investigated (eg cell line, species name).
- ➞ the assay(s) and method(s) used to carry out the reported observations and measurements.
- ➞ an explicit mention of the biological and chemical entity(ies) that are being measured.
- ➞ an explicit mention of the biological and chemical entity(ies) that are altered/varied/perturbed in a controlled manner.
- ➞ the exact sample size (n) for each experimental group/condition, given as a number, not a range;
- ➞ a description of the sample collection allowing the reader to understand whether the samples represent technical or biological replicates (including how many animals, litters, cultures, etc.).
- ➞ a statement of how many times the experiment shown was independently replicated in the laboratory.
- ➞ definitions of statistical methods and measures:
  - common tests, such as t-test (please specify whether paired vs. unpaired), simple χ2 tests, Wilcoxon and Mann-Whitney tests, can be unambiguously identified by name only, but more complex techniques should be described in the methods section;
  - are tests one-sided or two-sided?
  - are there adjustments for multiple comparisons?
  - exact statistical test results, e.g., P values = x but not P values < x;
  - definition of 'center values' as median or average;
  - definition of error bars as s.d. or s.e.m.

Please complete ALL of the questions below.
Select "Not Applicable" only when the requested information is not relevant for your study.

Materials

**Newly Created Materials**	**Information included in the manuscript?**	**In which section is the information available?** (Reagents and Tools Table, Materials and Methods, Figures, Data Availability Section)
New materials and reagents need to be available; do any restrictions apply?	Yes	Data Availability Section

**Antibodies**	**Information included in the manuscript?**	**In which section is the information available?** (Reagents and Tools Table, Materials and Methods, Figures, Data Availability Section)
For **antibodies** provide the following information:<br>- Commercial antibodies: RRID (if possible) or supplier name, catalogue number and or/clone number<br>- Non-commercial: RRID or citation	Yes	Reagent and Tools Table

**DNA and RNA sequences**	**Information included in the manuscript?**	**In which section is the information available?** (Reagents and Tools Table, Materials and Methods, Figures, Data Availability Section)
**Short novel DNA or RNA including primers, probes**: provide the sequences.	Yes	Reagent and Tools Table, Dataset EV3

**Cell materials**	**Information included in the manuscript?**	**In which section is the information available?** (Reagents and Tools Table, Materials and Methods, Figures, Data Availability Section)
**Cell lines:** Provide species information, strain. Provide accession number in repository **OR** supplier name, catalog number, clone number, and/**OR** RRID.	Yes	Reagent and Tools Table
**Primary cultures:** Provide species, strain, sex of origin, genetic modification status.	Yes	Methods, Reagent and Tools Table
Report if the cell lines were recently **authenticated** (e.g., by STR profiling) and tested for mycoplasma contamination.	Yes	Methods

**Experimental animals**	**Information included in the manuscript?**	**In which section is the information available?** (Reagents and Tools Table, Materials and Methods, Figures, Data Availability Section)
**Laboratory animals or Model organisms:** Provide species, strain, sex, age, genetic modification status. Provide accession number in repository **OR** supplier name, catalog number, clone number, **OR** RRID.	Yes	Methods, Reagent and Tools Table
**Animal observed in or captured from the field:** Provide species, sex, and age where possible**.**	Not Applicable	
Please detail **housing and husbandry conditions**.	Yes	Methods

**Plants and microbes**	**Information included in the manuscript?**	**In which section is the information available?** (Reagents and Tools Table, Materials and Methods, Figures, Data Availability Section)
**Plants:** provide species and strain, ecotype and cultivar where relevant, unique accession number if available, and source (including location for collected wild specimens).	Not Applicable	
**Microbes:** provide species and strain, unique accession number if available, and source.	Not Applicable	

**Human research participants**	**Information included in the manuscript?**	**In which section is the information available?** (Reagents and Tools Table, Materials and Methods, Figures, Data Availability Section)
If collected and within the bounds of privacy constraints report on age, sex and gender or ethnicity for all study participants.	Not Applicable	

**Core facilities**	**Information included in the manuscript?**	**In which section is the information available?** (Reagents and Tools Table, Materials and Methods, Figures, Data Availability Section)
If your work benefited from core facilities, was their service mentioned in the acknowledgments section?	Yes	Acknowledgments

Design

Study protocol	Information included in the manuscript?	In which section is the information available? (Reagents and Tools Table, Materials and Methods, Figures, Data Availability Section)
If study protocol has been **pre-registered, provide DOI in the manuscript**. For clinical trials, provide the trial registration number **OR** cite DOI.	Not Applicable	
Report the **clinical trial registration number** (at ClinicalTrials.gov or equivalent), where applicable.	Not Applicable	

Laboratory protocol	Information included in the manuscript?	In which section is the information available? (Reagents and Tools Table, Materials and Methods, Figures, Data Availability Section)
Provide DOI OR other citation details if **external detailed step-by-step protocols** are available.	Not Applicable	

Experimental study design and statistics	Information included in the manuscript?	In which section is the information available? (Reagents and Tools Table, Materials and Methods, Figures, Data Availability Section)
Include a statement about **sample size** estimate even if no statistical methods were used.	Yes	Methods
Were any steps taken to minimize the effects of subjective bias when allocating animals/samples to treatment (e.g. **randomization procedure**)? If yes, have they been described?	Yes	Methods
Include a statement about **blinding** even if no blinding was done.	Yes	Methods
Describe **inclusion/exclusion criteria** if samples or animals were excluded from the analysis. Were the criteria pre-established?<br><br>If sample or data points were omitted from analysis, report if this was due to attrition or intentional exclusion and provide justification.	Yes	Methods
For every figure, are **statistical tests** justified as appropriate? Do the data meet the assumptions of the tests (e.g., normal distribution)? Describe any methods used to assess it. Is there an estimate of variation within each group of data? Is the variance similar between the groups that are being statistically compared?	Yes	Methods

Sample definition and in-laboratory replication	Information included in the manuscript?	In which section is the information available? (Reagents and Tools Table, Materials and Methods, Figures, Data Availability Section)
In the figure legends: state number of times the experiment was **replicated** in laboratory.	Yes	Figure Legends
In the figure legends: define whether data describe **technical or biological replicates**.	Yes	Figure Legends

Ethics

Ethics	Information included in the manuscript?	In which section is the information available? (Reagents and Tools Table, Materials and Methods, Figures, Data Availability Section)
Studies involving **human participants**: State details of **authority granting ethics approval** (IRB or equivalent committee(s), provide reference number for approval.	Not Applicable	
Studies involving **human participants**: Include a statement confirming that **informed consent** was obtained from all subjects and that the experiments conformed to the principles set out in the WMA Declaration of Helsinki and the Department of Health and Human Services Belmont Report.	Not Applicable	
Studies involving **human participants:** For publication of **patient photos**, include a statement confirming that consent to publish was obtained.	Not Applicable	
Studies involving experimental **animals**: State details of **authority granting ethics approval** (IRB or equivalent committee(s), provide reference number for approval. Include a statement of compliance with ethical regulations.	Yes	Methods
Studies involving **specimen and field samples:** State if relevant **permits** obtained, provide details of authority approving study; if none were required, explain why.	Not Applicable	

Dual Use Research of Concern (DURC)	Information included in the manuscript?	In which section is the information available? (Reagents and Tools Table, Materials and Methods, Figures, Data Availability Section)
Could your study fall under dual use research restrictions? Please check biosecurity documents and list of **select agents and toxins** (CDC): https://www.selectagents.gov/sat/list.htm	Not Applicable	
If you used a select agent, is the security level of the lab appropriate and reported in the manuscript?	Not Applicable	
If a study is subject to dual use research of concern regulations, is the name of the **authority granting approval and reference number** for the regulatory approval provided in the manuscript?	Not Applicable	

Reporting

The MDAR framework recommends adoption of discipline-specific guidelines, established and endorsed through community initiatives. Journals have their own policy about requiring specific guidelines and recommendations to complement MDAR.

Adherence to community standards	Information included in the manuscript?	In which section is the information available? (Reagents and Tools Table, Materials and Methods, Figures, Data Availability Section)
State if relevant guidelines or checklists (e.g., **ICMJE, MIBBI, ARRIVE, PRISMA**) have been followed or provided.	Not Applicable	
For **tumor marker prognostic studies,** we recommend that you follow the **REMARK** reporting guidelines (see link list at top right). See author guidelines, under 'Reporting Guidelines'. Please confirm you have followed these guidelines.	Not Applicable	
For **phase II and III randomized controlled trials**, please refer to the **CONSORT** flow diagram (see link list at top right) and submit the CONSORT checklist (see link list at top right) with your submission. See author guidelines, under 'Reporting Guidelines'. Please confirm you have submitted this list.	Not Applicable	

Data Availability

Data availability	Information included in the manuscript?	In which section is the information available? (Reagents and Tools Table, Materials and Methods, Figures, Data Availability Section)
Have **primary datasets** been deposited according to the journal's guidelines (see 'Data Deposition' section) and the respective accession numbers provided in the Data Availability Section?	Yes	Data Availability Section
Were **human clinical and genomic datasets** deposited in a public access-controlled repository in accordance to ethical obligations to the patients and to the applicable consent agreement?	Not Applicable	
Are **computational models** that are central and integral to a study available without restrictions in a machine-readable form? Were the relevant accession numbers or links provided?	Yes	Data Availability Section
If publicly available data were reused, provide the respective **data citations in the reference list**.	Yes	References, Methods
